# An early-life survival and reproductive trade-off shapes selection on body size

**DOI:** 10.1093/evlett/qraf029

**Published:** 2025-09-13

**Authors:** Maria João Janeiro, Jonathan M Henshaw, Josephine M Pemberton, Jill G Pilkington, Michael B Morrissey

**Affiliations:** School of Biology, University of St Andrews, St Andrews KY16 9TH, United Kingdom; Institute of Biology I, University of Freiburg, Hauptstraße 1, 79104 Freiburg i. Br., Germany; Institute of Ecology and Evolution, School of Biological Sciences, University of Edinburgh, Edinburgh EH9 3FL, United Kingdom; Institute of Ecology and Evolution, School of Biological Sciences, University of Edinburgh, Edinburgh EH9 3FL, United Kingdom; School of Biology, University of St Andrews, St Andrews KY16 9TH, United Kingdom

**Keywords:** body size, causality, early pregnancy, natural selection, path analysis, Soay sheep

## Abstract

Evolutionary trade-offs—opposing trait effects on total fitness via different fitness components—are likely to be widespread. Some key trade-offs are expected to be the result of chains of causation acting across an organism’s lifetime. For example, a trait imparting reproductive benefits early in life may trade off against reduced survival to attain later-life reproductive opportunities. Tools in evolutionary quantitative genetics have recently been developed to formally characterize selection acting through different causal pathways throughout the life cycle and, therefore, to formally characterize evolutionary trade-offs. We use these methods to investigate a trade-off between early life reproduction and survival and how that trade-off affects selection on body size in the Soay sheep population inhabiting St Kilda (Outer Hebrides, Scotland). We decompose and quantify the total effects of first-year female body mass on lifetime fitness, with particular attention to the effect of body mass on early-life reproduction and the potential survival cost of early-life reproduction. Our results establish that the total effect of body mass on lifetime fitness is positive, despite the strong negative contribution acting via early life reproduction. Moreover, we show that the magnitude of the selection on body mass acting through different causal paths highly depends on population density. At higher densities, the cost of early-life reproduction is higher, and therefore, it contributes a strong negative component to the total selection of body mass—i.e., at higher population density, selection on body mass is weaker than it is when the population density is smaller. By decomposing total selection and quantifying selection acting through different causal paths, we expose the underlying mechanics shaping body mass in Soay sheep female lambs, and we provide a meaningful contribution to the understanding of the evolution of body size in this population.

## Introduction

Trade-offs occur when traits have both positive and negative effects on fitness, acting through different components of fitness (Stearns, 1989). A classic trade-off is that between current reproductive investment and survival, current survival being a pre-requisite for future reproductive opportunities. While such trade-offs have been demonstrated in different kinds of observational and manipulative studies (Reznick, 1985), evidence supporting their occurrence is by no means ubiquitous (Santos & Nakagawa, 2012). Signatures of these trade-offs are even more rare in the data from long-term observational studies that are typically available to study how interrelations among traits influence the generation-to-generation mechanics of selection. Specifically, among traits that are presumed to positively affect fitness, negative correlations are scarce, particularly at the phenotypic level, but also at the genetic level (Chang et al., 2024; Kruuk et al., 2008). Correlated responses to selection are particularly difficult to detect in the wild when the traits involved are determined by an overall acquisition resource, which may be largely or entirely non-genetic (De Jong & Van Noordwijk, 1992; Reznick et al., 2000). In that case, at the phenotypic level, both traits may covary positively, despite the existence of an inherent trade-off (Morrissey et al., 2012b). A key limitation of typical evolutionary quantitative genetic approaches, which rely to a large extent on phenotypic and genetic covariances to reflect trade-offs (Walsh & Blows, 2009; Walsh & Lynch, 2018), is that they do not accommodate chains of causal effects of traits on fitness very well (Conner, 1996; Scheiner et al., 2002). The ultimate causal structure of trade-offs is thus at odds with the standard ways of using phenotypic correlations among traits to separate total selection (selection differentials, Endler, 1986) from direct effects of traits on fitness (selection gradients, Lande, 1979). While direct selection gradients provide entirely valid evolutionary predictions, they constitute a statistical, rather than biological definition of selection, and can, therefore, lead to trivialization of the mechanisms of selection. In particular, evolution of traits that cause fitness variation indirectly and traits that are incidentally correlated with selected traits are both treated as cases of evolution due to genetic correlations in microevolutionary studies based only on direct selection gradients (Conner, 1996; Scheiner et al., 2002).

**Figure 1.**
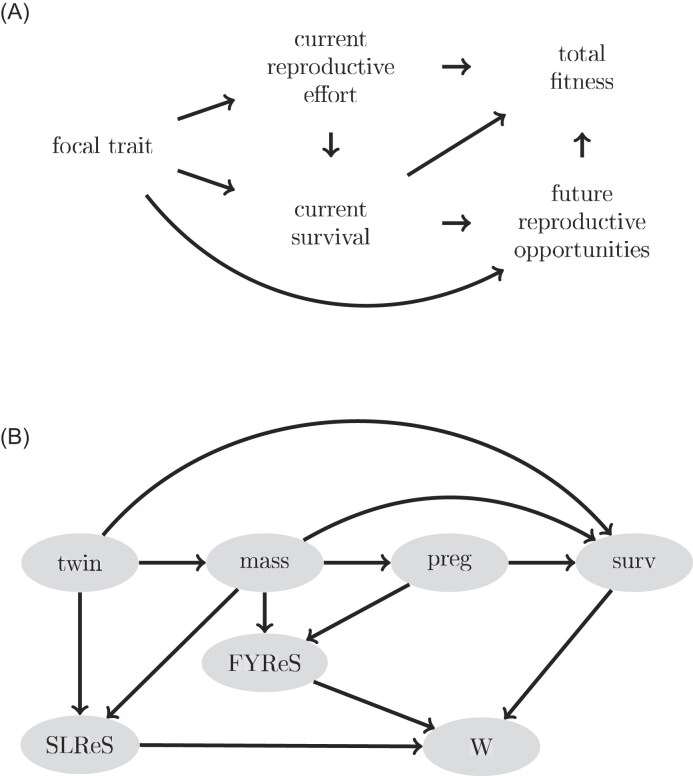
(A) Example of a diagram representing the total effect of a trait on fitness through different causal paths. The set of associations between traits depicted in this path diagram can be used to identify trade-offs between current reproductive effort and current survival, mediated by the focal trait, as described in the main text; (B) Path diagram representing the phenotypic landscape defined in Equation 7. The probability of being a twin, *twin*, has an effect on all the other traits, lamb body *mass* affects the probabilities of becoming pregnant as a lamb, *preg*, surviving the first annual cycle, *surv*, and also first-year and subsequent lifetime rearing success, *FYReS* and *SLReS*; and *preg* affects *surv*. Fitness, *W*, is defined as the sum of FYReS and SLReS on surviving females weighted by the probability of first-year survival.

There has recently been progress on formally incorporating causal effects among traits into the mechanics of predicting generation-to-generation evolutionary change. Morrissey (2014) showed how linear and additive effects of traits on one another, i.e., as represented by classical path analyses of selection (Conner, 1996; Scheiner et al., 2000) can be incorporated into an *extended selection gradient* that represents the total causal effects of traits on fitness, and how so-defined selection gradients relate to generation-to-generation evolutionary change. Morrissey (2015) shows how extended selection gradients can be characterized from causal relationships among traits that include additive, non-linear, and interactive (e.g., multiplicative) effects. Crucially, Henshaw et al. (2020) show how to decompose the extended selection gradient into contributions from different paths constituting a trait’s ultimate effect on fitness. Consequently, we now have the quantitative genetic tools to break down the total effect of traits on fitness for key trade-offs, such as between current reproduction and survival and future reproduction, including their inherent chains of causation. The full decomposition of the total selection of traits given by Henshaw et al. (2020) provides the opportunity to fully separate these direct and indirect chains of effects of traits on fitness that inherently underlie evolutionary trade-offs and to express those separate components in terms that relate directly to the generation-to-generation mechanics of adaptive evolution. A focal trait (body size, morphology, phenology) might, for example, influence a trait related to current reproductive effort (e.g., clutch size) and current survival directly, which may have immediate benefits to fitness (Figure 1A). Simultaneously, such effect on current survival would also affect future reproductive opportunities. In addition, this trait may be related to future reproductive performance, while simultaneously affecting survival via future reproductive opportunities through its effect on the current reproductive effort trait. Moreover, the direction and strength of selection on the focal trait acting via each path in Figure 1A is likely to change with changing environmental conditions (e.g., in a mild environment, selection might favor large body size and large clutches, but large clutches might be associated with lower current survival in harsh environments). Given how difficult it is to produce accurate evolutionary predictions in wild populations (Kruuk et al., 2014; Pujol et al., 2018), the ability to isolate and quantify the strength of selection acting through different paths and contributing to total selection is likely to be highly useful in complex systems. In addition to explicitly identifying and characterizing the potentially positive and negative pathways from a trait to total fitness that will manifest when a trade-off operates, such descriptions of the biological story behind selection can be generated across gradients of environmental variables, providing biologically motivated quantitative accounts of the environmental dependence of selection.

The theory developed in Morrissey (2015) and Henshaw et al. (2020) might prove valuable in characterizing a hypothesized evolutionary trade-off involving body mass in St Kilda Soay sheep (*Ovis aries*) and, therefore, improving our understanding of how selection is shaped in this population. A non-negligible percentage of females become pregnant as lambs (early pregnancy), i.e., during the rut that occurs when they are 5-6 months old, and those females are more likely to die during their first year of life (Clutton-Brock et al., 2004a). We hypothesize that these pregnancies are both (a) size-dependent and (b) detrimental to first-year survival, despite the fact that size is otherwise strongly positively associated with first-year survival and total reproductive output (Clutton-Brock et al., 1992, 1996; Milner et al., 2004). We, therefore, hypothesize that reproduction in the first year of life may be involved in a trade-off with first-year survival and future reproductive opportunities, which mediates total selection on body size. Population density is an important driver of vital rates and selection in this system (Clutton-Brock et al., 2004a; Hunter et al., 2018). Therefore, we further hypothesize that both total selection and contributions of different causal pathways from juvenile body size to lifetime fitness vary as a function of population density.

In this study, we first construct a series of statistical models of relationships among body mass (as a measure of a major component of body size), pregnancy, and survival in the first year of life, and subsequent fitness. We subsequently combine these models into a mathematical representation of the causal effects of first-year body mass on lifetime fitness (Figure 1B). We calculate these effects and decompose the component paths that characterize the hypothesized trade-off between early reproduction and first-year survival on total selection of lamb body mass. We apply all calculations of total selection and its decomposition across a range of population densities. Indeed, we provide clear evidence that first-year body mass has both positive and negative effects on fitness, the latter acting through early pregnancy. By characterizing and quantifying different contributions to selection on body mass and explaining how the strength of these contributions changes with the environment, our study provides an important contribution to the understanding of the evolution of size in female Soay sheep lambs.

## Methods and results

In this section, we first provide an overview of the study system, with particular reference to features relevant to the current analysis. We then describe the analysis and results of statistical models of key associations among traits and between traits and survival and reproduction. Finally, we describe how the individual models are combined into a selection analysis, accounting for the logical causal structure encoded by the set of statistical models, and which ultimately allows the decomposition of selection along causal pathways following Henshaw et al. (2020). Specifically, we use a path analysis involving the key traits needed to decompose the effect of lamb body mass on fitness via early pregnancy and otherwise (Figure 1B). The path diagram includes twin status, mass, early pregnancy, first-year survival, first-year rearing success, subsequent lifetime rearing success (SLReS), and absolute fitness, as defined in the following sections.

### Study system

#### Data collection

The population of Soay sheep inhabiting the Village Bay on St Kilda in the Outer Hebrides, Scotland, (57°49’N 08°34’W) has been the subject of an intensive, individual-based study since 1985 (Clutton-Brock & Pemberton, 2004b). In this population, the rut occurs in November, and most lambs are born between late March and early May. Although most females give birth to a single lamb, twinning rates vary between 2% and 23% (Clutton-Brock et al., 2004b). More than 95% of the individuals living in the study area have been marked with plastic ear tags shortly after birth to enable identification throughout their lifetimes. Maternal identity and twin status (singleton or twin) are ascertained from field observations. Regardless of whether a female’s lamb is captured (e.g., in cases of neonatal mortality), reproductive status is extensively recorded, allowing assessment of annual and lifetime fitness of females. Regular censuses and mortality searches provide death dates for the majority of the sheep. Each year in August a large portion of the study population is captured and phenotyped for multiple traits, including body mass. We focus on phenotypic and life history data on female lambs born between 1991 and 2015, corresponding to 3457 individuals, as there is no systematic record of pregnancy status from post-mortems of females that died during the winter before 1991. However, as measurements of lifetime fecundity only become available after individuals die, and mean and maximum longevity among females are 2.2 and 16.0 years, respectively, we only included cohorts up to 2006 when assessing lifetime reproduction (*n* = 550).

The pedigree of this population has been constructed through a combination of observational field data and molecular markers for maternal links and using molecular markers only for paternal links (Bérénos et al., 2014; Johnston et al., 2013). 315 polymorphic and unlinked SNP (Single Nucleotide Polymorphism) markers were used in molecular parentage assignments (for 4371 individuals) with 100% confidence in the R package MasterBayes (Hadfield et al., 2006). Polymorphic microsatellite markers were also used when SNP genotypes were not available for either the lamb or candidate fathers. In those cases, for a total of 222 lambs, 14–18 polymorphic microsatellite markers were used in assignments with confidence >95% in MasterBayes (Hadfield et al., 2006; Morrissey et al., 2012a). The resulting pedigree has a maximum depth of 10 generations and consists of 6,740 individuals, of which 6,336 are nonfounders (i.e. have one or two known parents). 74% of the females considered in our analysis were nonfounders, with 62%, 10%, and 2% with both parents, mother, and father of known identity, respectively.

#### Summary statistics of body mass and early pregnancy

**Figure 2.**
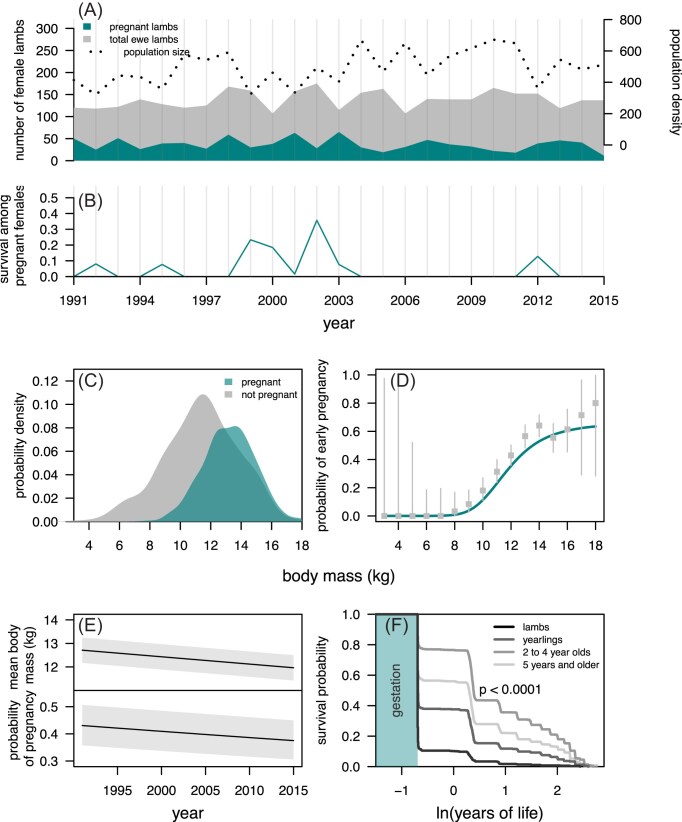
(A) Number of Soay sheep female lambs, female lambs pregnant, and population size (males and females) from 1991 to 2015 in the Village Bay study area; (B) first-year survival among pregnant females; (C) empirical distribution of body mass in pregnant and non-pregnant female lambs; (D) size-dependent probability of early pregnancy. The line in teal corresponds to the prediction of a binomial regression with logit link function, whereas the gray bars correspond to 95% confidence intervals on the binomial probability obtained from the raw data; (E) lamb body mass (upper panel) and probability of early pregnancy (lower panel) across time. The black lines correspond to predictions of a linear and a binomial model (logit link function), respectively, with year and population density (linear and quadratic terms) as fixed effects. The gray areas illustrate the variation in lamb body mass and probability of early pregnancy attributed to varying population density in 1 standard deviation from the mean; (F) non-parametric survival curves of Soay sheep according to mother’s age at conception. The *p*-value corresponds to a Peto & Peto test for differences among Kaplan–Meier curves.

Early pregnancy, here defined as a pregnancy resulting from conception during the first year of life, occurs at a rate of 37 per year in Soay sheep (with 914 records documented from 1991 to 2015), corresponding to yearly rates between 8% and 57% among female lambs. The proportion of lambs that become pregnant varies greatly among years, but the raw data suggest no strong temporal trend (Figure 2A). Body size is a key driver of these pregnancies, as the probability of pregnancy increases substantially with body mass (Figure 2C, D). Using body mass as our measure of body size, a relatively small female lamb, with a mass of 8–10 kg in August, is unlikely to become pregnant, whereas relatively large lambs, for example, with mass of 14 or more kg, are more likely than not to become pregnant (Figure 2D). It is well-documented that, on average, body mass in Soay sheep decreases in years with higher density (Clutton-Brock & Pemberton, 2004a; Ozgul et al., 2009). Conditional on population density, which has increased over time, there is a temporal trend of decreasing body mass in Soay sheep, including in lambs (Ozgul et al., 2009), and also in female lambs (Figure 2E, upper panel). This trend is concomitant with a decrease in the rate of early pregnancy over the years, when density is again accounted for (Figure 2E, lower panel). The negative trend in the probability of pregnancy over time might be explained by substantial costs and few benefits, including low survival (Figure 2B) and lower longevity in offspring born as a result of early pregnancy as compared to offspring born to older females (Figure 2F).

### Models of body mass, early pregnancy, survival, and fitness

In this section, we investigate associations among body mass, early pregnancy, survival, and parental success. The purpose of these models is to dissect all of the individual associations among key variables that contribute to overall selection on body mass and early pregnancy. Ultimately, these models parameterize the functions relating variables in a path analysis, allowing us to disentangle direct and indirect components of selection on body mass and early pregnancy.

We fitted statistical models with covariates that are included for two reasons: (1) we were interested in how the response variables vary as a function of these covariates, and (2) some covariates are included to account for their effect on the covariates of interest in the former group. Results and predictions are shown for average conditions of the variables in (2), either by mean-centering continuous variables or averaging our predictions over the probability associated with binary variables (particularly, twin status). Also, for clarity, simplified versions of model descriptions, mostly excluding the latter variables, are presented in the main text, but complete information is provided in Supporting Information SI.1. Finally, all interaction terms are omitted in equations shown in the main text but provided in Supporting Information SI.1.

We define parental success as the number of offspring successfully reared to November 1 of any year and distinguish between those offspring successfully reared as a result of early pregnancy (first-year rearing success, FYReS) and subsequently (subsequent lifetime rearing success). While these measures of reproductive fitness differ from previous definitions of breeding success (offspring produced over a lifetime) and reproductive success (offspring successfully raised up until the age of 1 year; Clutton-Brock (1988)), they delineate fitness associated with a female’s ability to gestate and provision a lamb from subsequent components of lamb survival, which may be less directly dependent on the mother.

#### Lamb body mass

A linear regression of the form


(1) \begin{eqnarray*}
m_{ijk} = \alpha + \alpha _t I_{t_{ijk}} + \pmb {\beta _d} f(d_{ijk},2) + \pmb {\beta } \pmb {X_{ijk}} + a_i + {u_c}_j + {u_m}_k + \epsilon _{ijk}
\end{eqnarray*}

was adopted to model body mass (*m*) of lamb *i*, born in year *j* to mother *k*. $\alpha$ and $\alpha _t$ are the intercept and contrast to the intercept for twin status ($I_t$), $\pmb {\beta _d}$ is a vector containing the linear (slope) and quadratic (curvature) terms associated to a second order polynomial function (*f*) on population density (*d*), and $\pmb {\beta }$ is a vector with the coefficients for the remaining fixed effects ($\pmb {X}$), including a second order polynomial function for maternal age and linear terms for birth and measurement dates (see Supporting Information SI.1). $a_i$ is the breeding value of lamb *i*, and ${u_c}_j$ and ${u_m}_k$ are random intercepts to characterize among-cohort and among-mother variation. Finally, $\epsilon _{ijk}$ is a residual term for individual *i*. Parameter estimates are provided in Supporting Information SI.1, Table SI.1.1.

#### Early pregnancy

The probability of early pregnancy (*p*) was estimated using a binomial regression with a logit link function,


(2) \begin{eqnarray*}
\ln \left(\frac{p_{ijk}}{1-p_{ijk}}\right) &=& \alpha + \alpha _t I_{t_{ijk}} + \pmb {\beta _m} f(m_{ijk},3) + \pmb {\beta _d} f(d_{ijk},2)\\
&&+\, \pmb {\beta } \pmb {X_{ijk}} + {u_c}_j + {u_m}_k + \epsilon _{ijk},
\end{eqnarray*}

where $\pmb {\beta _m}$ corresponds to a vector with coefficients for the linear, quadratic, and cubic terms for body mass, *m*. In this model, *X* includes an interaction term for body mass and population density, a second-order polynomial function for maternal age, and linear terms for birth and measurement dates. The remaining quantities have analogous meanings as in Equation 1. More generally, although corresponding to different quantities, we use the same symbols for equivalent coefficients in different models throughout the manuscript. Finally, as the overdispersion variance is unobservable for Bernoulli (binomial with a single trial) response variables, the variance in $\epsilon$ was set to one. This is standard practice using MCMCglmm (Hadfield, 2010), and when the unit over dispersion variance is appropriately marginalized, it does not affect inference (Morrissey et al., 2014). Parameter estimates are provided in Supporting Information SI.1, Table SI.1.2. Model (2) corroborates the hypothesis that early pregnancy is size-dependent, as suggested by the exploratory analyses in Figures 2C and D, with larger lambs being more likely to become pregnant.

#### First-year survival

We investigated whether early pregnancy has fitness costs by evaluating first-year survival in female lambs as a function of pregnancy and body mass. First-year survival was defined using the first day of May as the cut-off date to make sure that dying during labor was considered in the first annual cycle of the females (89% of births from early pregnancies occur before 1 May). Females with known first-year survival status and that survived until the rut in their first year of life, i.e., that had the opportunity to become pregnant in their first year of life, were included in the analysis (*n* = 947). A binomial regression with a logit link function of the form


(3) \begin{eqnarray*}
\ln \left(\frac{s_{ijk}}{1-s_{ijk}}\right) &=& \alpha + \alpha _t I_{t_{ijk}} + \alpha _p I_{p_{ijk}} + \beta _m m_{ijk} + \pmb {\beta _d} f(d_{ijk},2)\\
&& +\, \pmb {\beta } \pmb {X_{ijk}} + {u_c}_j + {u_m}_k + \epsilon _{ijk},
\end{eqnarray*}

was adopted to model the probability that lamb *i*, born in year *j* to mother *k* survives its first year of life. $\alpha _p$ is a contrast to the intercept for pregnancy status ($I_p$). Although not pictured in Equation 3, a pregnancy-specific contrast to the slope for mass was also estimated. Additionally, $\bf {X}$ also includes interaction terms between density and pregnancy, and density, pregnancy and body mass, a second-order polynomial function for maternal age, and birth and measurement dates (Table SI.1.3 in Supporting Information SI.1). As first-year survival, and survival in general, is highly dependent on cohort effects due to variability in winter conditions and food availability (Clutton-Brock et al., 2004a), we included cohorts as random effects (${u_c}_j$), as well as maternal environmental effects (${u_m}_k$). Variance in residuals ($\epsilon _{ijk}$) was set to one, as overdispersion is unobservable in Bernoulli mixed models.

**Figure 3.**
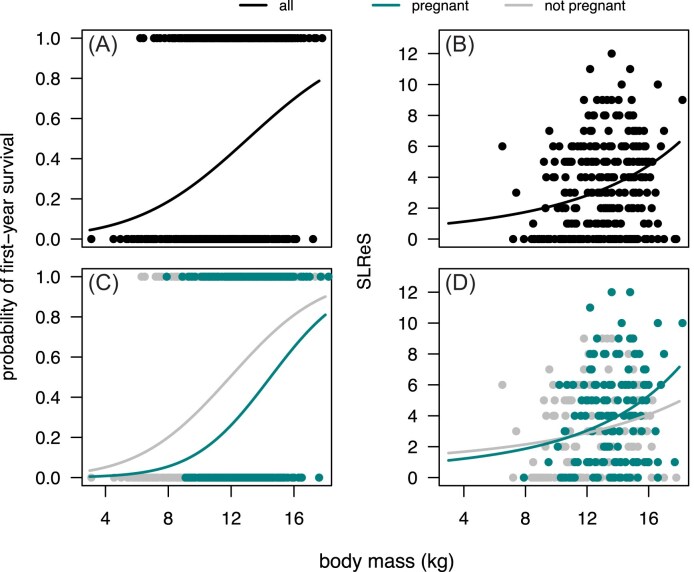
Probability of first-year survival (A, C) and subsequent lifetime rearing success, SLReS, (B, D) in Soay sheep females as a function of lamb body mass (A, B) and both lamb body mass and early pregnancy status (C, D). Dots correspond to observed data and curves to model predictions. Binomial regressions for first-year survival with logit link function were fitted to the data. SLReS was estimated in females that survived their first year of life using Poisson regressions with exponential link function. In all models, population density and maternal age were included as covariates (linear and quadratic terms were estimated for both), as well as birth and measurement dates (see Tables SI.1.3 and SI.1.5 in Supporting Information SI.1). Variance among cohorts and maternal identities was also estimated.

We first established that first-year survival is size-dependent by adopting a similar model to that in Equation 3, but excluding the pregnancy-specific contrasts—bigger females have higher chance of survival (*p*  $<$ .001, Figure 3A, Table SI.1.3a in Supporting Information SI.1). Adopting the full model in Equation 3 shows that for a given body mass, pregnant lambs are substantially less likely to survive their first annual cycle when compared to the females that do not get pregnant (*p*  $<$ .001, Figure 3C, Table SI.1.3b in Supporting Information SI.1). Lamb pregnancy has a substantial survival cost—the probability of survival for female lambs of average body mass that did not become pregnant is 50% (95% CrI 35%; 68%), an estimate that drops to 24% (95% CrI 11%; 38%) in pregnant lambs. Pregnant lambs of all sizes are more likely to die than non-pregnant female lambs of the same size (Figure 3C). It is interesting to notice that mean body mass is lower among *all* lambs that became pregnant (14.00, 95% CrI 13.65; 14.48) than it is in the sub-group of these lambs that survived at least a year (14.59, 95% CrI 14.11; 15.03), suggesting that if a female is to be large enough to become pregnant, then it is better to be as large as possible. Investigating the simultaneous effect of lamb body mass, early pregnancy, and population density on first-year survival exposes an important interaction effect between the former traits and the latter. Although large population density is, in general, associated with lower survival, it is evident that pregnant female lambs are more susceptible to variation in population density than lambs that do not become pregnant (Supporting Information SI.2, Figure SI.2.1a,b). First-year survival in pregnant females is very low (below 10%) across most possible values of population density, unless body mass is relatively very high, whereas such low yearly survival rates are much less likely in non-pregnant females.

#### First-year rearing success

Having established that pregnant female lambs are more likely to die when compared to same-sized non-pregnant female lambs, it is important to understand the fate of those pregnancies. From the 914 documented early pregnancies, only 97 resulted in offspring surviving until the winter (FYReS), and only 36, less than 5%, resulted in recruitment to the population, i.e., offspring that survived until their first April 1. We estimated the FYReS of female lamb *i* by fitting a binomial regression with a logit link,


(4) \begin{eqnarray*}
\ln \left(\frac{\mathrm{FYReS}_i}{1-\mathrm{FYReS}_i}\right) = \alpha + \beta _m m_i + \pmb {\beta _d} f(d_{ijk},2) + \pmb {\beta } \pmb {X_i} + \epsilon _{i},
\end{eqnarray*}

with $\bf {X}$, including birth and measurement dates, and the remaining terms taking an analogous meaning as in previous models. The variance in the model residuals, $\epsilon _i$, was set to one. Due to sample size limitations, we did not consider any random effects. This model was fitted for females that became pregnant and survived their first winter, as those who did not become pregnant and did not survive have a FYReS of zero.

Only 2.2% (95% CrI 0.57%; 6.17%) of the offspring born to females that became pregnant as average-sized lambs in average-density years survive until the winter. It is interesting to note that the slope for body mass is positive and very likely different from zero (Table SI.1.4 in Supporting Information SI.1), showing that larger female lambs are more likely to have surviving offspring, and suggesting that, as for first-year survival, if a female lamb is to become pregnant, than the larger the better. Overall, FYReS not only increases with female body mass but also substantially decreases as population density increases (Figure SI.2.1c in Supporting Information SI.2).

#### Subsequent lifetime rearing success

We also investigated the effect of lamb body mass and early pregnancy on SLReS, consisting of lifetime rearing success excluding offspring reared in the first reproductive opportunity (FYReS). SLReS reflects any fitness differences among individuals surviving to one year of age, whether they arise through variation in longevity or annual reproductive output. As the SLReS of females dying during their first year of life is zero, analyses presented here only include females that survived. The SLReS of female *i*, born in year *j* to mother *k* was modeled with a Poisson mixed model with a $\log$ link function of the form


(5) \begin{eqnarray*}
\ln (\mathrm{SLReS}_{ijk}) &=& \alpha + \alpha _t I_{t_{ijk}} + \alpha _p I_{p_{ijk}} + \beta _m m_{ijk} + \pmb {\beta _d} f(d_{ijk},2)\\
&& + \pmb {\beta } \pmb {X_{\bf {ijk}}} + u_{c_j} + u_{m_k} + \epsilon _{ijk},
\end{eqnarray*}

with $\bf {X}$ including a pregnancy-specific contrast to the slope for body mass, interaction terms between density and early pregnancy, and density, early pregnancy, and body mass, a second-order polynomial function for maternal age, and linear terms for birth and measurement dates. The remaining terms take analogous meaning as in previous models (Table SI.1.3 in Supporting Information SI.1). To study SLReS, the data were constrained to only include females from cohorts that were completely phenotyped for this trait, which was achieved by excluding all females born after 2006 (*n* = 550). This procedure resulted in including very few SLReS records associated with females that were still alive by the cut-off year (2015). While these surviving females’ lifetime fitness is underestimated, they have reared most of the offspring they will rear in their lives. Excluding these records would result in stronger bias because females that lived longer had potentially higher rearing success.

We applied the model in Equation 5 without the coefficients associated with early pregnancy and established that SLReS is dependent on lamb body mass (Figure 3B, Table SI.1.5a in Supporting Information SI.1). Rearing success is an increasing function of lamb body mass, with bigger animals being more likely to rear more offspring. The full model investigates the presence of a benefit of experience gained from a previous pregnancy, an effect independent from that of body mass, and it suggests that rearing success is not determined by early pregnancy status (Figure 3D, Table SI.1.5b in Supporting Information SI.1). Neither the contrast to the intercept (0.37; 95% CrI −0.06; 0.82) nor the interaction terms with body mass (0.02; 95% CrI −0.05; 0.07) and population density ($3.4\times 10^{-3}$; 95% CrI $-8.67\times 10^{-4}$; $7.46\times 10^{-3}$) are likely different from zero, however, most of the posterior density of the pregnancy-related coefficients supports positive values. Assessing the simultaneous effects of body mass and population density on SLReS, independent of early pregnancy, shows that not only is SLReS highest in females born at low population densities, but also that the effect of lamb body mass on SLReS is stronger in females that were also born in years of low population density (Supporting Information SI.2; Figure SI.2.1d).

#### Twin status

The parameterization of the path model (Figure 1B) requires knowledge of the effects of twin status on body mass, early pregnancy, survival, and SLReS, represented in Equations 1, 2, 3, and 5, respectively. Furthermore, given the binary nature of the variable, a model of the probability of being a twin is required so that the quantities such as mean phenotype or selection gradients can be averaged over this probability. For this purpose, even though much is known about the probability of producing a singleton versus twins in the Soay sheep of St Kilda, a binomial mixed model was fitted according to


(6) \begin{eqnarray*}
\ln \left(\frac{t_{ijk}}{1-t_{ijk}}\right) = \alpha + \pmb {\beta _d} f(d_{ijk},2) + \pmb {\beta } \pmb {X_{ijk}} + {u_c}_j + {u_m}_k + \epsilon _{ijk},
\end{eqnarray*}

with terms defined analogously to previous models. $\pmb {X}$ in this model includes a quadratic function for maternal age. Detailed information on the estimated quantities is provided in Supporting Information SI.1, Table SI.1.6, which corroborates previous knowledge that twinning rates vary with maternal age and environmental conditions (Clutton-Brock et al., 2004a; Wilson et al., 2009).

#### Model fitting

Models were fitted with MCMCglmm (Hadfield, 2010), using inverse gamma distributions for residual variances (of non-binary traits) and inverse Wishart distributions for the (co)variance components associated with random effects as prior distributions. Also, parameter expansion was implemented to increase convergence efficiency (Gelman, 2006; Hadfield, 2010). Model convergence was assessed by visual examination of trace and density plots of posterior distributions. We also made sure parameters were estimated based on reasonable effective sample sizes. Parameter point estimates and 95% credible intervals correspond to means and high posterior density intervals of posterior distributions, respectively.

### Selection analysis

The analyses shown so far establish that (a) early pregnancy is body mass-dependent, (b) there is selection against early pregnancy through first winter viability selection, (c) the lifetime fitness benefit of early pregnancy is very small, and (d) SLReS in surviving females is body mass-dependent but not demonstrably different in females that became and did not become pregnant. In this section, we use the information presented so far in a formal selection analysis to investigate *selection for* (i.e., arising due to causal effects of the focal trait on fitness) lamb body mass in Soay sheep females. In particular, we use a path analysis involving all pertinent traits (Figure 1B), allowing the estimation of an *extended selection gradient* (Morrissey, 2014, 2015). We then use the twin-network approach of Henshaw et al. (2020) to decompose the effect of lamb body mass on fitness into components via early pregnancy and otherwise. The path diagram (Figure 1B) includes twin status, *t*, mass, *m*, early pregnancy, *p*, first-year survival, *s*, first-year rearing success, subsequent lifetime rearing success, and absolute fitness, *W*, and can be represented by a vector-valued function of the following form:


(7) \begin{eqnarray*}
\pmb {z}_{ijk} &=& {\left[\begin{array}{l}t\\
m\\
p\\
s\\
\mathrm{FYReS}\\
\mathrm{SLReS}\\
\mathrm{W} \end{array}\right]}_{ijk} = \pmb {f(l)}_{ijk}\\
&=& {\left[\begin{array}{l}g^{-1}(\alpha + \pmb {\beta _d} f(d_{ijk},2) + \pmb {\beta } \pmb {X_{ijk}} + {u_c}_j + {u_m}_k + \epsilon _{ijk}) \mathrm{(Eqn. {6})}\\
\alpha + \alpha _t I_{t_{ijk}} + \pmb {\beta _d} f(d_{ijk},2) + \pmb {\beta } \pmb {X_{ijk}} + a_i + {u_c}_j + {u_m}_k + \epsilon _{ijk} \mathrm{(Eqn. {1})}\\
g^{-1}(\alpha + \alpha _t I_{t_{ijk}} + \pmb {\beta _m} f(m_{ijk},3) + \pmb {\beta _d} f(d_{ijk},2) + \pmb {\beta } \pmb {X_{ijk}} + {u_c}_j + {u_m}_k + \epsilon _{ijk}) \mathrm{(Eqn. {2})}\\
g^{-1}(\alpha + \alpha _t I_{t_{ijk}} + \alpha _p I_{p_{ijk}} + \beta _m m_{ijk} + \pmb {\beta _d} f(d_{ijk},2) + \pmb {\beta } \pmb {X_{ijk}} + {u_c}_j + {u_m}_k + \epsilon _{ijk}) \mathrm{(Eqn. {3})} \\
g^{-1}(\alpha + \beta _m m_{i} + \pmb {\beta _d} f(d_{i},2) + \pmb {\beta } \pmb {X_{i}} + \epsilon _{i}) \mathrm{(Eqn. {4})}\\
g^{-1}(\alpha + \alpha _t I_{t_{ijk}} + \beta _m m_{ijk} + \pmb {\beta _d} f(d_{ijk},2) + \pmb {\beta } \pmb {X_{ijk}} + {u_c}_j + {u_m}_k + \epsilon _{ijk}) \mathrm{(Eqn. {5})}\\
s_{ijk} \,\, (\mathrm{FYReS}_{i}p_{i}+\mathrm{SLReS}_{ijk}) \end{array}\right]},\\
\end{eqnarray*}

with *z* being a multivariate phenotype including the traits defined above. Note that in this path analytical model, SLReS reflects both reproductive investment and survival beyond the first year of age. The equation numbers given in brackets refer to the statistical models used to parameterize each component, with corresponding symbols taking the same meaning as previously described. The inverse link functions, $g^{-1}$ in Equation 7, correspond to the logistic function in the models for twinning, pregnancy, survival, and first-year rearing success and the exponential function in the model for SLReS. Fitness was defined as the sum of FYReS of females that became pregnant, weighted by the probability of early pregnancy, and survived their first annual cycle, and SLReS of females that survived their first annual cycle, both weighted by the probability of first-year survival.

Villemereuil et al. (2016) derived exact expressions to convert the latent scale parameters estimated in generalized linear mixed models into scales in which traits are expressed and selected. Following their work, the expected values for *z* ($\bar{z}$) are given by


(8) \begin{eqnarray*}
\pmb {\bar{z}} = \int \pmb {g^{-1}(\ell )} f_{\mathcal {MVN}}(\pmb {\ell },\pmb {\mu }, \pmb {P_\ell }) \, \mathrm{d}{\ell },
\end{eqnarray*}

where $\pmb {g}^{-1}$ corresponds to the inverse link functions used in the statistical models, $\pmb {\ell }$ are latent values for each trait (in the scale defined by the link functions), and $f_{\mathcal {MVN}}(\pmb {\ell },\pmb {\mu }, \pmb {P_\ell })$ is the probability density of a multivariate normal distribution with vector mean $\pmb {\mu }$ and (co)variance $\pmb {P_\ell }$. To obtain population density-specific values, $\pmb {\mu }$ contained latent scale predictions based on the model intercept and the estimated density effect. Likewise, the phenotypic variance-covariance matrix in the scale in which traits are expressed is given by


(9) \begin{eqnarray*}
\pmb {P} = \int \left(\pmb {g}^{-1}\pmb {(\ell )} - \pmb {\bar{z}}\right)^2 f_{\mathcal {MVN}}(\pmb {\ell }, \pmb {\mu }, \pmb {P_\ell }) \, \mathrm{d}{\ell }.
\end{eqnarray*}

Although population density is not included in the path diagram (Figure 1B), all the results presented in this section were obtained by marginalizing over the observed population density in all cohorts—i.e., Equations 8 and 9 were applied to the vector-valued developmental function given by Equation 7 for every observed value of population density, and mean values taken as the corresponding best estimates. Means and variances are given for average values of maternal age, measurement, and birth dates. We estimate $\pmb {\bar{z}}$ as


\begin{eqnarray*}
\pmb {\bar{z}} = {\left[\begin{array}{l}\bar{t}\\
\bar{m}\\
\bar{p}\\
\bar{s}\\
\bar{\mathrm{FYReS}}\\
\bar{\mathrm{SLReS}}\\
\bar{\mathrm{W}} \end{array}\right]} = {\left[\begin{array}{l}0.21 \: (0.16; 0.25)\\
12.71 \: (12.53; 12.88)\\
0.48 \: (0.46; 0.49)\\
0.57 \: (0.55; 0.59)\\
0.08 \: (0.07; 0.08)\\
3.00 \: (2.48; 3.53)\\
1.94 \: (1.64; 2.34) \end{array}\right]},
\end{eqnarray*}

and **P** as shown in Table 1. The correlations between lamb body mass and first-year survival (0.42, 95% CrI 0.33; 0.51) and between early pregnancy and first-year survival (0.10, 95% CrI 0.03; 0.19) are both positive, suggesting that despite the direct viability cost associated with early pregnancy (Table SI.1.3b in Supporting Information SI.1), the positive direct effect of lamb body mass on first-year survival more than offsets its negative impact via early pregnancy (Table 1). To investigate the consequences of this trade-off in the selection of lamb body mass, we calculated its *extended* directional selection gradient. For a univariate trait $z_j$ (e.g., body mass), the extended directional selection gradient is defined as the expected *causal* derivative (*sensu* Henshaw et al., 2020) of relative fitness with respect to $z_j$ taken over the joint probability distribution of all traits (Henshaw et al., 2020), or equivalently, the average partial derivative of fitness with respect to independent inputs to the system (Morrissey, 2015),


(10) \begin{eqnarray*}
\eta _j = \mathbb {E}\left(\frac{\partial W(\ell _j)}{\partial \ell _j} \right) \overline{W}^{-1}.
\end{eqnarray*}

**Table 1.** Phenotypic variance-covariance matrix for the path diagram in Figure 1, including the probability of being a twin (*t*), body mass (*m*), the probability of early pregnancy (*p*), the probability of first-year survival (*s*), first-year rearing success ($fyres$), subsequent lifetime rearing success ($slres$), and fitness, defined as lifetime rearing success (FYReS+SLReS) in surviving females weighed by the probability of survival. The diagonal corresponds to variances, the upper off-diagonal to covariances, and the lower off-diagonal to correlations among traits. Values are shown in the scale, traits are expressed, and values within brackets correspond to 95% highest posterior density credible intervals.

	*t*	*m*	*p*	*s*	fyres	slres	*W*
*t*	0.08 (0.06; 0.11)	-0.26 (-0.34; -0.16)	-0.01 (-0.02; -0.01)	-0.02 (-0.02; -0.01)	-0.01 (-0.01; 0.00)	-0.06 (-0.14; 0.00)	-0.08 (-0.15; -0.04)
*m*	-0.44 (-0.50; -0.34)	4.22 (3.55; 4.56)	0.32 (0.27; 0.36)	0.25 (0.20; 0.30)	0.15 (0.12; 0.17)	1.32 (0.85; 2.01)	1.61 (1.18; 2.23)
*p*	-0.15 (-0.22; -0.08)	0.56 (0.48; 0.64)	0.08 (0.07; 0.09)	0.01 (0.00; 0.01)	0.01 (0.01; 0.01)	0.09 (0.04; 0.18)	0.09 (0.04; 0.15)
*s*	-0.20 (-0.27; -0.12)	0.42 (0.33; 0.51)	0.10 (0.03; 0.19)	0.08 (0.07; 0.11)	0.01 (0.01; 0.01)	0.07 (0.03; 0.16)	0.31 (0.24; 0.40)
fyres	-0.20 (-0.25; -0.16)	0.59 (0.57; 0.63)	0.27 (0.20; 0.33)	0.25 (0.19; 0.31)	0.01 (0.01; 0.02)	0.05 (0.02; 0.10)	0.07 (0.04; 0.12)
slres	-0.05 (-0.11; 0.00)	0.19 (0.11; 0.25)	0.09 (0.04; 0.16)	0.08 (0.02; 0.13)	0.10 (0.04; 0.20)	9.87 (4.59; 35.67)	6.80 (3.92; 25.56)
W	-0.10 (-0.15; -0.04)	0.27 (0.18; 0.34)	0.11 (0.04; 0.16)	0.34 (0.24; 0.43)	0.18 (0.11; 0.30)	0.88 (0.80; 0.93)	9.52 (3.90; 23.01)

Equivalently, instead of being defined in terms of observed phenotypes $z_j$, $\eta _j$ is expressed in terms of the corresponding latent values, $\ell _j$, therefore accommodating phenotypes that are modeled probabilistically (e.g., early pregnancy). This selection gradient represents the ultimate effect of small changes in phenotype, $\ell _j$, on absolute fitness, *W*, averaged over the range of conditions experienced by individuals in the population. The algorithm for implementing the calculation described by Equation 10 is detailed in Supporting Information SI.5.

**Figure 4.**
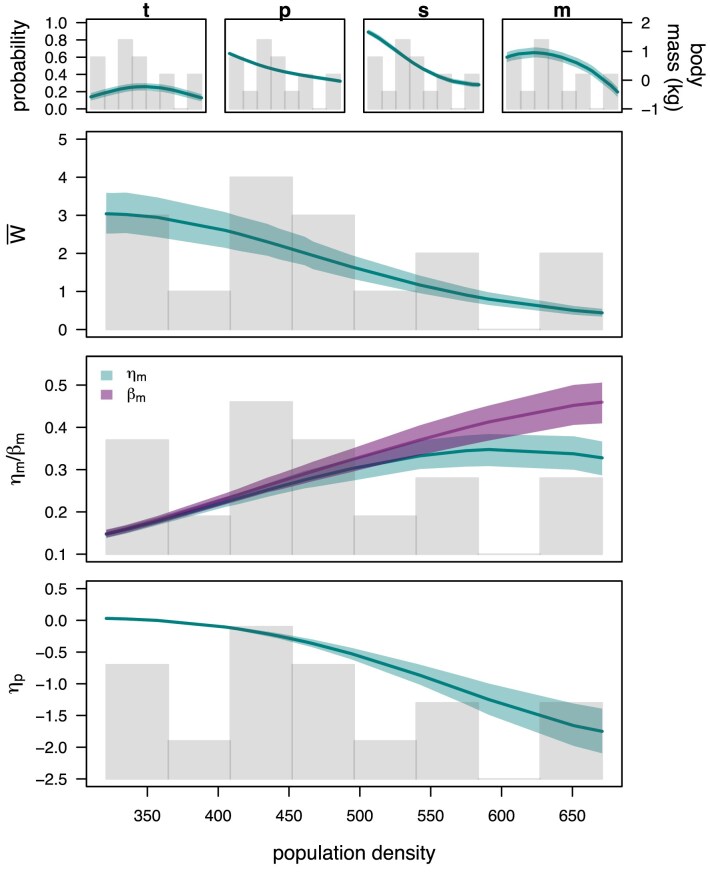
The effect of population density on traits depicted in the path diagram in Figure 1, and consequently on selection of lamb body mass and early pregnancy. The upper panel shows variation in mean twin status (*t*), early pregnancy (*p*), first-year survival (*s*), and mean-centered lamb body mass (*m*). The three lower panels include functions of mean fitness, selection of lamb body mass ($\eta _m$ and $\beta _m$), and selection of early pregnancy ($\eta _p$) on population density. All plots include a histogram showing the empirical distribution of population density. Shaded areas correspond to 95% highest posterior density credible intervals.

The extended selection gradient for mass is positive, $\eta _m =$ 0.26 (95% CrI 0.24; 0.27), whereas for lamb pregnancy it is negative, $\eta _p =$ −0.53 (95% CrI −0.62; −0.43). Importantly, the magnitude, but not the sign, of the conflict between viability and fecundity is density-dependent, and it is almost non-existent when population density is very low. In such circumstances, virtually all female lambs survive, and therefore early pregnancy does not have a significant cost. As a consequence, $\eta _p$ is higher (i.e., less strongly favoring low values) when population density is at its lowest and decreases with population density (Figure 4, Table 2).

**Table 2.** Selection gradients of lamb body mass and early pregnancy for three different values of observed population density—minimum, mean, and maximum (321, 452, and 671, respectively). Raw ($\eta$) and standardized selection gradients, either by the observed standard deviation ($\eta _{sd}$) or the observed mean ($\eta _{\mu }$), are shown. For lamb body mass, selection gradients considering first-year survival as a measure of fitness are also included ($\eta _s$). Values within brackets correspond to 95% highest posterior density credible intervals.

		*d* = 321	*d* = 452	*d* = 671
Body mass	$\eta$	0.15 (0.14; 0.16)	0.28 (0.26; 0.30)	0.33 (0.29; 0.37)
	$\eta _{sd}$	0.34 (0.32; 0.36)	0.65 (0.60; 0.70)	0.76 (0.66; 0.85)
	$\eta _{\mu }$	1.83 (1.72; 1.95)	3.49 (3.22; 3.75)	4.07 (3.56; 4.56)
	$\eta _{s}$	0.04 (0.04; 0.05)	0.15 (0.13; 0.16)	0.13 (0.11; 0.16)
	$\eta _{s_{sd}}$	0.10 (0.08; 0.11)	0.34 (0.29; 0.38)	0.31 (0.25; 0.37)
	$\eta _{s_{\mu }}$	0.53 (0.45; 0.60)	1.83 (1.58; 2.05)	1.66 (1.33; 1.98)
Early pregnancy	$\eta$	0.03 (0.02; 0.04)	−0.37 (-0.43; -0.30)	−1.75 (-2.10; -1.39)
	$\eta _{sd}$	0.02 (0.01; 0.02)	−0.18 (-0.21; -0.15)	−0.88 (-1.05; -0.70)

To investigate the mechanism by which lamb body mass influences fitness, we also calculated two path-specific selection gradients (Henshaw et al., 2020), acting via pregnancy ($\eta _{m \rightarrow p}$) and via all other pathways ($\eta _{m,\textrm{remaining}}$). The latter gradient describes that part of the effect of lamb body mass on fitness that is independent of its effect on pregnancy. In this case, $\eta _{m,\textrm{remaining}}$ can be calculated as the direct selection gradient $\beta _m$ on body mass while controlling for pregnancy. The gradient $\eta _{m,\textrm{remaining}}$, or $\beta _m$, is thus blind to the positive effect of lamb body mass on pregnancy and therefore to the pregnancy-mediated viability selection on body mass. The pregnancy-specific selection gradient $\eta _{m \rightarrow p}$ is then simply the difference between the extended selection gradient $\eta _m$ and $\beta _m$. On average, direct and total effects of mass on fitness do not differ much, and $\beta _m$ is only marginally larger (0.29; 95% CrI 0.27; 0.31) than $\eta _m$. The reason for that lies again on the dependency of these quantities on population density. $\eta _m$ and $\beta _m$ are very similar when population density is very low, as viability selection tends to zero and, therefore, there is no cost to early pregnancy. However, we show that $\eta _m$ and $\beta _m$ diverge substantially with increasing population density (Figure 4, Table 2), indicating that the mediation of selection for body mass by pregnancy is highly density-dependent.

**Figure 5.**
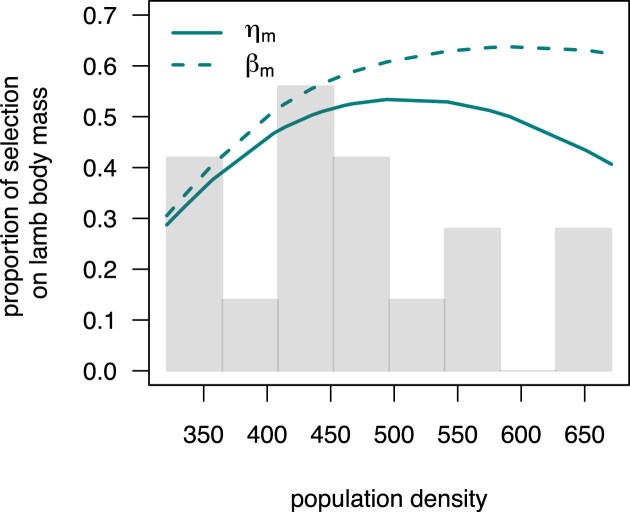
Proportion of selection on lamb body mass occurring through viability selection in Soay sheep females as a function of population density. This proportion was obtained both using the extended ($\eta _m$) and direct ($\beta _m$) directional selection gradient of lamb body mass. The histogram in gray shows the empirical distribution of population density.

The path analysis represented by Figure 1B invokes a definition of fitness that includes the entire life cycle. However, the mechanisms through which selection acts, survival and fecundity, occur at different points of the life of Soay sheep females. Viability selection is particularly strong in the first annual cycle, whereas most offspring are reared at older ages (only 2.2% of the offspring born to females that became pregnant as average-sized lambs in average-density years survive until the winter). To evaluate the strength of the two mechanisms is, therefore, also to understand in which period selection is the strongest. We quantified the contribution of selection on lamb body mass that occurs during the first year of life using a path diagram similar to the one in Figure 1B, but defining fitness as first-year survival. Measured this way, $\eta _m$ is 0.12 (95% CrI 0.11; 0.14), and therefore corresponds to about half of the total lifetime selection on body mass (Figure 5). Selection acting on lamb body mass through viability costs during the first year of life is proportionally very low when population density is lowest (around 30%), is highest at intermediate population densities, and decreases when population density is very high. A slightly different pattern is obtained if $\beta _m$, instead of $\eta _m$, is used to quantify the strength of selection. In this case, the relative importance of viability selection does not decrease when population density is at its highest. These two trajectories, put together, suggest that at low densities, when both fecundity and survival are very high (Figure 4  *p,s*), fecundity governs selection on lamb body mass, as yearling mothers (females that become pregnant as lambs) can successfully rear offspring. As population density increases and first-year survival decreases, viability selection becomes proportionally more important as it occurs before any opportunity for reproduction. At the higher end of naturally occurring population densities in this system, the probability of lamb survival is very low and fairly constant (Figure 4*s*). As a result, at these high densities, the cost of early pregnancy to viability selection is smaller, and the relative contribution from fecundity selection to total selection slightly increases. When inspecting $\beta$, this pattern is not apparent at higher population densities. As $\beta$ is naïve to the correlation between lamb body mass and early pregnancy, it does not capture the opposing effects body mass has on first-year survival (directly *versus* via early pregnancy), underlying the pattern observed for $\eta$.

### Alternate formulations of the bivariate relationship between lamb mass and early pregnancy

Reasonable alternate formations of the relationship between lamb mass and early pregnancy might treat the two as a bivariate component of the phenotype or might treat pregnancy as a plastic response to mass wherein there may be genetic variation for the reaction norm slope. In order to verify that key findings do not differ if these alternate formations are adopted, we conducted two supplemental sets of analyses.

#### Bivariate formulation

Quantifying the genetic association between body mass and early pregnancy provides a means of isolating the causal and spurious components of selection on body mass, as such associations are a signature of causal relationships between the two traits (Morrissey et al., 2010; Rausher, 1992). To investigate whether such an association exists, in the form of additive genetic covariance, we used an animal model corresponding to a multi-response generalized linear mixed model (Hadfield, 2010) for body mass and the probability of lamb pregnancy. Consistent with the strong effect of mass on the probability of pregnancy, we estimated substantial phenotypic and genetic correlations between female lamb mass and early pregnancy (Tables SI.3.1 and SI.3.2).

#### Reaction norm formulation

Finally, we conducted a complementary analysis of selection wherein we allowed for genetic variation among individuals in the slope of the probabilistic reaction norm of early pregnancy as a function of lamb body mass (see Supporting Information SI.4). This involved modeling the probability of early pregnancy using an expanded version of Equation 2 that allowed for genetic variation in pregnancy status, given mass via a random regression animal model (Wilson et al., 2010). Note that since early pregnancy status is only expressed once during life, non-genetic components of the individual reaction norm are not identifiable, but because pregnancy status of relatives is available, the genetic component is estimable. We then expanded Equation 7 to include probabilistic maturation reaction norm intercepts and slopes as separate latent traits, and we used this expanded mapping of traits onto one another and fitness to estimate extended selection gradients of both the intercept and slope (i.e., using Equation 10). Selection is predominantly negative for the maturation reaction norm intercept, in line with our overall finding of negative selection on early pregnancy status. Additionally, our approach suggests little selection on the probabilistic reaction norm slope (Figure SI.4.1).

## Discussion

We have characterized a trade-off between early life reproduction and survival, shaping selection of body mass in female Soay sheep lambs. Larger female lambs are more likely to become pregnant during their first annual cycle, and pregnant lambs are more likely to die than non-pregnant lambs of similar body mass. As a consequence, natural selection acts on lamb body mass through two distinct pathways: through the direct effect of lamb body mass on fitness, independent of early pregnancy status, and through its effect on early pregnancy. Selection on lamb body mass would, therefore, be stronger (more positive) if not for the relationship between this trait and early pregnancy. As a result, this correlation is one mechanism that contributes to shaping selection of body size in Soay sheep.

Recent theoretical advances enable the decomposition of selection according to component causal pathways, essentially by using a system of path analyses that allows for non-linear effects and interactions (Henshaw et al., 2020; Morrissey, 2015). We used this theory to disentangle and quantify the direct effect of lamb body mass on fitness and that arising via its effect on early pregnancy. Alternatives to this approach are the use of traditional path analysis (developed for linear traits, e.g. Latta & McCain, 2009; Morrissey, 2014) or multi-response (linear or generalized linear) models that explicitly estimate (additive genetic) correlations among traits (e.g. Bonnet et al. 2017). As compared to multi-response explicit genetic models, the approach we adopted might prove particularly useful when inference on the genetic architecture among traits is challenging. The current analysis makes an assumption of a causal (phenotypic) effect of mass on pregnancy status. This assumption alleviates the need for more involved inference of genetic covariances among the traits, as is a more typical quantitative genetic approach for assessing constraints (but see Supporting Information SI.3 for a bivariate analysis). The path analytic approach laid out by Morrissey (2015) and Henshaw et al. (2020) specifically harnesses the phenotypic relationship between traits—in our study, lamb body mass and early pregnancy—and builds it into a formal model of selection, thus circumventing the use genetic models and the challenges in fitting such models. Notably, information available suggests that the most considerable obstacle in dealing with the selection of correlated traits is not purely methodological. Rather, detecting genetic correlations seems to be a limiting step, with evidence for genetic constraints in the wild being rather scarce (Kruuk et al., 2008). Reasons for this may include insufficient knowledge about the biology and ecology of a species (Pemberton, 2010), but also the presence of confounding effects between genes and environment. Negative genetic covariation between two traits might be obscured at the phenotypic level by environmental covariation among traits (Morrissey et al., 2012b; Roff & Fairbairn, 2007). While the approach adopted here is not immune to these challenges, by relying on a conceptually explicit characterization of selection, it might contribute to their mitigation. Irrespective of specific analytical tools, we believe that further empirical studies focusing on genetic trade-offs are needed to advance our understanding of their role in the widespread mismatch between predicted evolutionary change and observed dynamics of body size (Blanckenhorn, 2000; Hansen & Houle, 2004; Kruuk et al., 2008).

Characterizing trade-offs involving early survival might often be associated with particular complexity. In general, fully incorporating the effects of early life viability selection is very challenging, because it fundamentally causes individuals to be *missing not at random* (Hadfield, 2008; Nakagawa & Freckleton, 2008) later in life. Simply put, we cannot know what phenotypes individuals would have expressed for late-life traits, if they have died early in life. The problem is not insurmountable (Hadfield, 2008; Mittell & Morrissey, 2024; Steinsland et al., 2014), but it is challenging indeed. In our analyses, we benefited from the very comprehensive and consistent data collection effort of the Soay sheep project, which includes a systematic search of individuals that die during the winter and their post-mortem. Such protocol allows, not only to assess over-winter mortality, which disproportionally affects lambs, but also to determine what fecundity the female lambs would have achieved, had they survived the winter, as pregnancy can be identified during post-mortems.

Soay sheep have existed on an island, Soay, which is adjacent to Hirta, where the long-term study of Soay sheep occurs, since prehistoric times (Clutton-Brock & Pemberton, 2004b). Soay and Hirta have similar population dynamics, and as such, the associations among population density, body mass, early pregnancy, and fitness reported here may be broadly representative of the long-term pattern selection experienced by Soay sheep (Grenfell et al., 1998). However, the current distribution of phenotype in the Soay sheep on Hirta, particularly their propensity to become pregnant in the first year of life, may be an adaptation to recent selection. Soay sheep were reintroduced to Hirta in 1932, when 107 individuals were moved from Soay (Clutton-Brock & Pemberton, 2004b), following the evacuation of the last human residents of Hirta. During the immediately subsequent decades of lower population density (Clutton-Brock et al., 2004a), selection likely favored a much faster life history. While results presented here are derived from data for the range of observed population densities since 1991, they are strongly indicative of what selection would happen at lower population densities. At lower population densities, survival rates of female lambs would be very high, regardless of pregnancy status (Figure SI.2.1 in Supporting Information SI.2). Additionally, the survival of offspring of females that became pregnant as lambs is extremely strongly density dependent (Table SI.1.4 in Supporting Information SI.1), and as such there would be a clear benefit, and little or no survival cost, to early reproduction at smaller population densities, such as those experienced by Soay sheep on Hirta during a key period in the last century.

A trade-off between early fecundity and survival in females might, together with an equivalent mechanism occurring in males, play a key role in shaping selection on body mass in Soay sheep. While we do not directly assess whether the trade-off occurring in females contributes to shaping selection on body mass in males, the existence of a strong positive genetic correlation in body mass across sexes (Robinson et al., 2009) might, in principle, facilitate that effect. Like females, male Soay sheep also participate to varying degrees in the rut during their first year of life (Chapman et al., 2023; Coltman et al., 1999). Additionally, evidence gathered in males so far: (a) fitness is positively associated with body size (Milner et al., 2004), (b) larger lambs are more likely to participate in the rut than smaller ones (Stevenson et al., 2004), and (c) rut activity has been associated with lower survival (Stevenson et al., 2004), is suggestive of a trade-off between early-life survival and reproductive success shaping the selection of body size in males not dissimilar to that observed in females.

## Summary

This study provides evidence of trade-off between early reproduction and survival, and how it mediates total lifetime selection on body size during the first year of life in female Soay sheep (*Ovis aries*). While body mass is positively selected overall—large lambs have the highest lifetime fitness—this selection is substantially attenuated by a survival-reproduction trade-off. Large female lambs are more likely to become pregnant, and pregnant lambs of a given body mass are more likely to die during the winter than non-pregnant lambs of the same body mass. The attenuation of selection by this trade-off is trivial at low population density but substantial at high population density. The study showcases involved, but nonetheless widely applicable, methods for characterizing complex mechanisms of natural selection. This approach to decomposing total selection could prove broadly useful for characterizing a wide variety of processes, including trade-offs, that influence selection in complex ways.

## Supplementary Material

qraf029_Supplemental_File

## Data Availability

The data underlying this article are available in the Dryad Digital Repository at https://doi.org/10.5061/dryad.44j0zpct6. In addition, see Supporting Information SI.6 for the R code used for the analyses presented in this manuscript.
