# Supplementary material for: An early-life survival and reproductive trade-off shapes selection on body size
Source: Evol Lett. 2025 Sep 13;9(6):637–50. doi: 10.1093/evlett/qraf029 (PMC12676467; doi:10.1093/evlett/qraf029)
Supplement: qraf029_Supplemental_File [file qraf029_supplemental_file.pdf]

Supporting Information to "An early-life survival and reproductive trade-off shapes selection on body size”

SI. 1 Model summary tables

Lamb body mass in August is dependent on birth and measurement dates. Both are indicators of how long lambs had the opportunity to grow and are particularly relevant because higher growth rates occur during the warmer months (before August), when vegetation is most available Crawley et al. (2004). As a result, Julian birth and measurement dates were included as covariates when modelling lamb body mass or lamb body mass was used as a predictor. Fixed effects for twin status, population density and maternal age at parity, including quadratic terms for the latter two, were included in all regressions, as well as the following linear interaction terms: between lamb body mass and population density in models including lamb body mass as predictor, between early pregnancy status and population density in models including early pregnancy as a predictor, and between these three traits in models including both lamb body mass and early pregnancy status as predictors. Likewise, random effects to estimate among-mother and among-cohort variation were included in all regressions. This fixed and random structure was applied to all statistical models except the one modelling FYReS (due to lack of statistical power).

Table SI.1.1: Coefficients of a linear regression of body mass in Soay female lambs. All covariates, except for twin status, were mean-centred. Variances in breeding values, random cohorts and maternal identity were estimated. Values within brackets correspond to 95% highest posterior density credible intervals. See Equation (1) in the main text for a model description.

parameter	posterior mode	95% CrI
intercept	1.60	(1.02; 2.07)
density ($\times$100)	-0.27	(-0.59; 0.05)
density^2 ($\times$100)	0.00	(-0.01; 0.00)
twin status	-2.91	(-3.21; -2.62)
maternal age	0.36	(0.29; 0.40)
maternal age^2	-0.11	(-0.13; -0.10)
birth date	-0.06	(-0.08; -0.05)
measurement date	0.09	(0.04; 0.13)
additive genetic variance:	0.67 (0.07; 1.14)	
variance in cohort effects:	0.71 (0.33; 1.08)	
variance in maternal effects:	0.59 (0.29; 1.21)	
residual variance:	1.81 (1.47; 2.33)	

Table SI.1.2: Coefficients of a binomial regression (logit link function) of early pregnancy on body mass in Soay female lambs. Other fixed effects include population density, twin status, and maternal age. All covariates, except for twin status, were mean-centred. Variances in random cohorts and maternal identity were also estimated. Values within brackets correspond to 95% highest posterior density credible intervals. See Equation (2) in the main text for a model description.

parameter	posterior mode	95% CrI
intercept	-0.81	(-1.39; -0.12)
body mass	0.69	(0.49; 0.87)
body mass^2	-0.11	(-0.18; -0.07)
body mass^3	0.01	(-0.01; 0.02)
density ($\times$100)	-0.45	(-0.89; -0.15)
density^2 ($\times$100)	0.00	(0.00; 0.01)
density $\times$ body mass ($\times$100)	0.11	(0.02; 0.23)
twin status	0.76	(0.17; 1.45)
maternal age	-0.03	(-0.13; 0.07)
maternal age^2	0.00	(-0.03; 0.03)
birth date	-0.02	(-0.05; 0.02)
measurement date	-0.07	(-0.12; 0.01)
variance in cohort effects:	0.46 (0.14; 1.27)	
variance in maternal effects:	0.01 (0.00; 1.34)	

Table SI.1.3: Coefficients of mixed effect binomial regressions with logit link function exploring the association of first-year survival with body mass and early pregnancy in Soay females; (a) body mass-dependent first-year survival, (b) first-year survival as a function of body mass and early pregnancy. Covariates except for twin and pregnancy status were mean-centred. See Equation (3) in the main text for a model description.

parameter	posterior mode	95% CrI
(a)		
intercept	-0.55	(-1.59; 0.38)
body mass	0.50	(0.36; 0.66)
density (×100)	-1.38	(-1.95; -0.72)
density^2 (×100)	0.01	(0.00; 0.01)
density × body mass (×100)	-0.06	(-0.18; 0.05)
twin status	0.06	(-0.70; 0.74)
maternal age	-0.02	(-0.12; 0.12)
maternal age^2	0.01	(-0.03; 0.05)
birth date	0.02	(-0.02; 0.05)
measurement date	-0.10	(-0.20; -0.03)
variance in cohort effects: 1.60 (0.78; 4.18)		
variance in maternal effects: 1.82 (0.61; 4.22)		
(b)		
intercept	0.14	(-0.95; 1.23)
pregnancy	-2.15	(-2.73; -1.35)
body mass	0.69	(0.47; 0.89)
body mass × pregnancy	0.19	(-0.18; 0.43)
density (×100)	-0.89	(-1.75; -0.28)
density^2 (×100)	0.00	(0.00; 0.01)
density × pregnancy (×100)	-1.35	(-2.04; -0.70)
density × body mass (×100)	0.10	(-0.07; 0.24)
density × body mass × pregnancy (×100)	-0.04	(-0.34; 0.27)
twin status	0.19	(-0.74; 0.88)
maternal age	0.04	(-0.12; 0.14)
maternal age^2	0.00	(-0.04; 0.04)
birth date	0.01	(-0.03; 0.05)
measurement date	-0.15	(-0.23; -0.04)
variance in cohort effects: 2.04 (0.98; 5.11)		
variance in maternal effects: 2.23 (0.85; 4.89)		

Table SI.1.4: Coefficients of a binomial regression (logit link function) of FYReS on body mass, birth and measurement dates, and population density in Soay sheep female lambs that became pregnant and survived their first winter. All covariates were mean-centred, and values within brackets correspond to 95% highest posterior density credible intervals. See Equation (4) in the main text for a model description.

Parameter	Posterior mode	95% CrI
intercept	-4.00	(-5.08; -2.94)
body mass	0.64	(0.43; 1.02)
density (×100)	-1.06	(-2.41; -0.11)
density^2 (×100)	0.00	(0.00; 0.01)
birth date	0.03	(-0.04; 0.10)
measurement date	0.06	(-0.11; 0.16)

Table SI.1.5: Coefficients of Poisson regressions with logarithm link functions, exploring the association between SLReS in females surviving their first year of life, lamb body mass and early pregnancy; (a) body mass-dependent SLReS, and (b) SLReS as a function of lamb body mass and early pregnancy. Covariates except for twin status and early pregnancy status were mean-centred, and values within brackets correspond to 95% highest posterior density credible intervals. See Equation (5) in the main text for a model description.

parameter	posterior mode	95% CrI
(a)		
intercept	0.64	(0.12; 0.99)
body mass	0.14	(0.04; 0.23)
density (×100)	-0.15	(-0.49; 0.01)
density^2 (×100)	0.00	(0.00; 0.00)
density × body mass (×100)	0.04	(-0.06; 0.09)
twin status	0.07	(-0.38; 0.52)
maternal age	0.00	(-0.08; 0.06)
maternal age^2	0.01	(0.00; 0.03)
birth date	0.01	(-0.02; 0.03)
measurement date	0.01	(-0.06; 0.07)
variance in cohort effects:	0.00 (0.00; 0.37)	
variance in maternal effects:	0.38 (0.00; 0.68)	
residual variance:	0.45 (0.20; 0.86)	
(b)		
intercept	0.38	(-0.07; 0.83)
pregnancy	0.37	(-0.06; 0.82)
body mass	0.14	(0.03; 0.27)
pregnancy × body mass	0.02	(-0.28; 0.09)
density (×100)	-0.25	(-0.52; 0.06)
density^2 (×100)	0.00	(0.00; 0.00)
density × pregnancy (×100)	0.34	(-0.09; 0.75)
density × body mass (×100)	0.10	(0.00; 0.21)
density × body mass × pregnancy (×100)	-0.24	(-0.39; -0.06)
twin status	0.14	(-0.34; 0.57)
maternal age	0.00	(-0.08; 0.07)
maternal age^2	0.02	(0.00; 0.04)
birth date	0.00	(-0.02; 0.03)
measurement date	0.02	(-0.05; 0.07)
variance in cohort effects:	0.00 (0.00; 0.38)	
variance in maternal effects:	0.30 (0.00; 0.60)	
residual variance:	0.51 (0.26; 0.91)	

Table SI.1.6: Coefficients of a binomial regression (logit link function) of the probability of being a twin as a function of mean-centred population density and maternal age. Values within brackets correspond to 95% highest posterior density credible intervals. See Equation (6) in the main text for a model description.

parameter	posterior mode	95% CrI
intercept	-2.47	(-3.37; -1.43)
density ($\times$100)	-0.07	(-0.47; 0.55)
density^2 ($\times$100)	-0.01	(-0.01; 0.00)
maternal age	0.90	(0.69; 1.14)
maternal age^2	-0.15	(-0.20; -0.08)
variance in cohort effects:	0.72 (0.15; 2.59)	
variance in maternal effects:	9.25 (5.02; 14.35)	

SI. 2 Supplementary figures

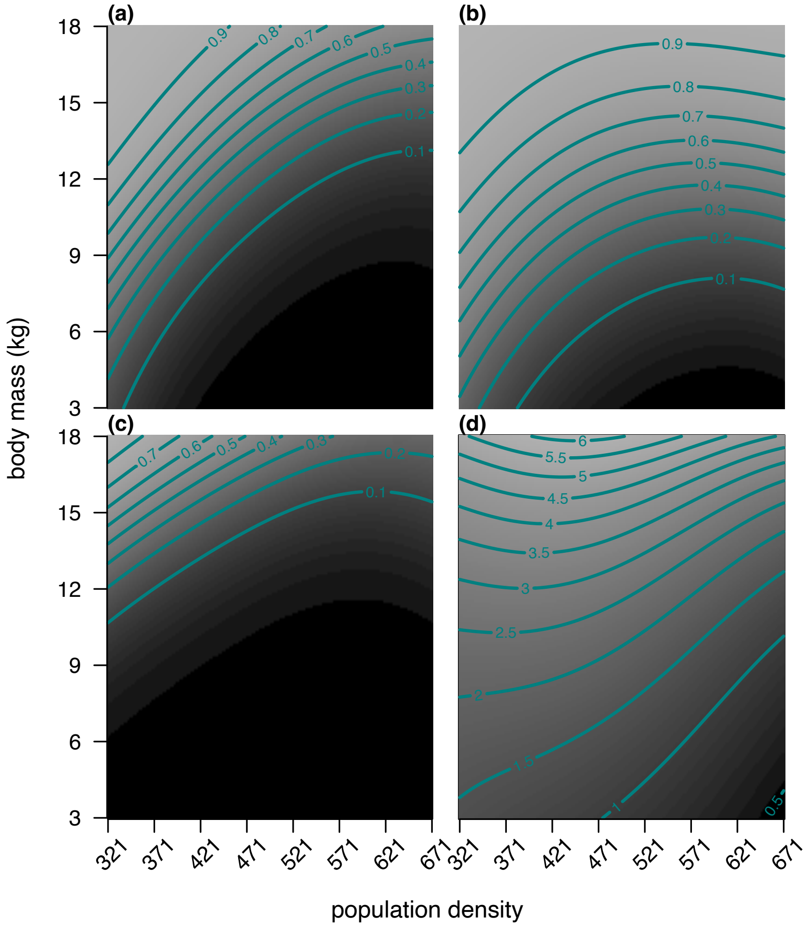

Figure SI.2.1: Probability of first-year survival in pregnant (a) and non-pregnant (b) female lambs, and FYReS (c) and SLReS (d) in Soay sheep females that survived their first annual cycle as a function of lamb body mass and population density. These are predictions based on models in Tables SI.1.3, SI.1.4 and SI.1.5 (Supplementary Information 1). Alt text: "Four plots representing first-year survival in pregnant (a) and non-pregnant (b) female lambs, and FYReS (c) and SLReS as a function of body mass and population density."

SI. 3 Bivariate association of early pregnancy and body mass

Quantifying the genetic association between body mass and early pregnancy provides a means of isolating the causal and spurious components of selection on body mass, as such associations are a signature of causal relationships between the two traits Rausher (1992); Morrissey et al. (2010). To investigate whether such an association exists, in the form of additive genetic covariance, we used an animal model corresponding to a multi-response generalised linear mixed model Hadfield (2010) for body mass, m, and the probability of lamb pregnancy, p. As lamb pregnancy is Bernoulli-distributed, a latent scale variable, p_ℓ, was defined such that $p_\ell = \ln\left(\frac{p}{1-p}\right)$. As such, both m and p_ℓ are assumed to be drawn from a multivariate normal distribution, with a mean vector that includes both mean mass, μ_m, and mean pregnancy probability in the logit scale, μ_{p_ℓ}, and a variance-covariance matrix $\boldsymbol{P_\ell}$,

$$\begin{bmatrix} m \\ p_\ell \end{bmatrix} \sim \mathcal{MVN}\left(\begin{bmatrix} \mu_m \\ \mu_{p_\ell} \end{bmatrix}, \boldsymbol{P_\ell}\right). \tag{SI.3.1}$$

Both μ_m and μ_{p_ℓ} depend on twin status (singleton or twin), population density, and maternal age at parity, whereas the former also depends on birth and measurement dates. The corresponding parameters and their estimates are listed in Table SI.3.1. The subscript ℓ denotes *latent scale*, which here applies to early pregnancy only, since lamb body mass was modelled on its natural scale via an identity link function. The genetic and environmental contributions to $\boldsymbol{P_\ell}$ were partitioned by including random effects on breeding values (animal model, Henderson (1975)). A $\boldsymbol{G_\ell}$ matrix and an $\boldsymbol{E_\ell}$ matrix can be defined such that

$$\boldsymbol{P_\ell} = \begin{bmatrix} \sigma^2_m & \sigma_{m,p_\ell} \\ \sigma_{m,p_\ell} & \sigma^2_{p_\ell} \end{bmatrix} = \begin{bmatrix} \sigma^2_{m_a} & \sigma_{m_a,p_{\ell_a}} \\ \sigma_{m_a,p_{\ell_a}} & \sigma^2_{p_{\ell_a}} \end{bmatrix} + \begin{bmatrix} \sigma^2_{m_e} & \sigma_{m_e,p_{\ell_e}} \\ \sigma_{m_e,p_{\ell_e}} & \sigma^2_{p_{\ell_e}} \end{bmatrix}, \tag{SI.3.2}$$

where subscripts a and e denote the additive genetic and the environmental contribution to the overall (co)variances, respectively Falconer (1981). To simplify the notation, we do not use any extra subscript to denote phenotypic (co)variances (see matrix $\boldsymbol{P_\ell}$ in Equation SI.3.2). Besides residual variances and covariances, the matrix $\boldsymbol{E_\ell}$ also includes variances and covariances associated with cohort and maternal random effects. Since the overdispersion variance of a generalised linear mixed model is unobservable for Bernoulli (binomial with a single trial) response variables, the residual variance for p_ℓ was set to one. Estimates for $\boldsymbol{G_\ell}$ and $\boldsymbol{P_\ell}$ are presented in Table SI.3.2.

Taking into account the additive genetic and the phenotypic variances in lamb body mass, 0.78 (95% credible interval [CrI] 0.20; 1.27) and 3.85 (95% CrI 3.39; 4.45), respectively, the heritability of this trait is estimated

to be 0.16 (95% CrI 0.05; 0.32)[1], very similar to the estimate reported by Bérénos et al. (2014) for both sexes (0.12, SE 0.036). Villemereuil et al. (2016) derived exact expressions to convert the latent scale parameters estimated in generalised linear mixed models into scales in which traits are expressed and selected. We used their results to derive the parameters in the model for early pregnancy in the probability rather than the logit scale. Following Villemereuil et al. (2016), the expected values for body mass and the probability of early pregnancy are given by

$$\bar{\boldsymbol{z}} = \begin{bmatrix} \bar{m} \\ \bar{p} \end{bmatrix} = \int \boldsymbol{g}^{-\mathbf{1}}(\boldsymbol{\ell}) f_{\mathcal{MVN}}(\boldsymbol{\ell}, \boldsymbol{\mu}, \boldsymbol{P_\ell})\, \mathrm{d}\ell\,, \qquad \text{(SI.3.3)}$$

where $\boldsymbol{g}^{-1}$ corresponds to the inverse link functions used to model body mass and the probability of early pregnancy. As the adopted link functions for these variables were the identity and the logit functions, respectively, their inverses are the identity and logistic functions, the latter corresponding to $\frac{\exp(p_\ell)}{\exp(p_\ell)+1}$. $\boldsymbol{\ell}$ are the latent values for lamb body mass and early pregnancy. $f_{\mathcal{MVN}}(\boldsymbol{\ell}, \boldsymbol{\mu}, \boldsymbol{P_\ell})$ is the probability density of a multivariate normal distribution with vector mean $\boldsymbol{\mu}$ and variance $\boldsymbol{P_\ell}$. Likewise, the phenotypic variance-covariance matrix is given by

$$\boldsymbol{P} = \int \left(\boldsymbol{g}^{-1}(\boldsymbol{\ell}) - \bar{\boldsymbol{z}}\right)^2 f_{\mathcal{MVN}}(\boldsymbol{\ell}, \boldsymbol{\mu}, \boldsymbol{P_\ell})\, \mathrm{d}\ell\,. \qquad \text{(SI.3.4)}$$

The $\boldsymbol{G}$ matrix

$$\boldsymbol{G} = \boldsymbol{\Phi} \boldsymbol{G_\ell} \boldsymbol{\Phi}^T, \qquad \text{(SI.3.5)}$$

can also be derived Villemereuil et al. (2016), where $\boldsymbol{\Phi}$ is the average derivative of the expected values with respect to the latent values,

$$\boldsymbol{\Phi} = \int \frac{\mathrm{d}\boldsymbol{g}^{-1}}{\mathrm{d}\boldsymbol{\ell}} f_{\mathcal{MVN}}(\boldsymbol{\ell}, \boldsymbol{\mu}, \boldsymbol{P_\ell})\, \mathrm{d}\ell\,. \qquad \text{(SI.3.6)}$$

Estimates of $\boldsymbol{P}$ and $\boldsymbol{G}$ are presented in Table SI.3.2, and details on how derivatives and integrals were solved are provided in Supporting Information 5. The heritability in the scale in which traits are expressed is obtained directly through its definition, as the proportion of the additive genetic variance relative to the phenotypic variance, using the derived values. The heritability of early pregnancy on the probability scale is 0.40 (95% CrI 0.12; 0.64), corroborating the genetic basis of this trait. Using the derived values on the probability scale, we also obtained the conditional genetic variance of lamb body mass. This measure allows

[1]The values provided correspond to the mode and 95% credible intervals of the posterior distribution of the heritability.

us to evaluate the proportion of the additive genetic variance in lamb body mass that is independent of early pregnancy. Following Hansen et al. (2003), this quantity, $\sigma^2_{m_a|p_a}$, is given by

$$\sigma^2_{m_a|p_a} = \sigma^2_{m_a} - \frac{{\sigma_{m_a,p_a}}^2}{\sigma^2_{p_a}}, \tag{SI.3.7}$$

which in this particular case equals 0.40 (95% CrI 0.06; 0.85). The analogous metric for early pregnancy corresponds to 0.03 (95% CrI 0.00; 0.05). Since $\sigma^2_{m_a}$ and $\sigma^2_{p_a}$ were estimated to be, respectively, 0.78 (95% CrI 0.20; 1.27) and 0.03 (95% CrI 0.00; 0.08, Tab. SI.3.2), the percentage of the additive genetic variance in lamb body mass and early pregnancy that are independent from one another corresponds to 59% (95% CrI 29%; 100%). Substantial proportions of the genetic variances in lamb body mass and early pregnancy are not independent, and therefore *selection for* each one of these traits necessarily results in *selection of* the other.

Table SI.3.1: Coefficients of the bivariate model of lamb body mass and early pregnancy in Soay sheep females. Values within brackets correspond to 95% highest posterior density credible intervals. See Equations (SI.3.1) and (SI.3.2) for a model description.

parameter		posterior mode	95% CrI
fixed effects			
body mass	intercept	13.821	(13.215; 14.261)
	density	-0.002	(-0.005; 0.001)
	density^2	0.000	(0.000; 0.000)
	twin status	-2.972	(-3.245; -2.652)
	maternal age	0.391	(0.330; 0.436)
	maternal age^2	-0.108	(-0.125; -0.095)
pregnancy	intercept	0.000	(-0.850; 0.636)
	density	-0.005	(-0.009; -0.001)
	density^2	0.000	(0.000; 0.000)
	twin status	1.851	(1.055; 2.273)
	maternal age	-0.140	(-0.268; -0.023)
	maternal age^2	0.043	(-0.003; 0.071)
random effects			
breeding values	body mass	0.785	(0.199; 1.272)
	body mass:pregnancy	0.307	(-0.001; 1.494)
	pregnancy	0.695	(0.001; 5.859)
cohort	body mass	0.596	(0.266; 1.141)
	body mass:pregnancy	0.385	(0.136; 1.163)
	pregnancy	0.932	(0.136; 1.163)
maternal ID	body mass	0.577	(0.349; 1.014)
	body mass:pregnancy	0.002	(-0.159; 0.414)
	pregnancy	0.005	(0.000; 0.824)
residuals	body mass	1.940	(1.387; 2.224)
	body mass:pregnancy	1.059	(0.699; 1.267)
	pregnancy	1.000	(1.000; 1.000)

Table SI.3.2: Additive genetic (***G***) and phenotypic (***P***) variance-covariance matrices, as estimated by the bivariate model of lamb body mass and early pregnancy in Soay sheep females. Matrices in both the latent and probability scales are presented. Values correspond to posterior modes and 95% highest posterior density credible intervals. See Equations (SI.3.1) and (SI.3.2) for a model description.

	G		***P***	
	body mass	pregnancy	body mass	pregnancy
body mass	0.78 (0.20; 1.27)		3.85 (3.39; 4.45)	
pregnancy (latent)	0.31 (0.00; 1.49)	0.69 (0.00; 5.86)	2.35 (1.53; 3.37)	3.21 (1.81; 9.51)
pregnancy (probability)	0.09 (0.00; 0.18)	0.03 (0.00; 0.08)	0.33 (0.26; 0.42)	0.09 (0.07; 0.14)

SI. 4 Selection of the probabilistic reaction norm for early pregnancy

We expanded our selection analysis to take an explicit probablistic maturation reaction norm perspective. Essentially, rather than treating the slope of the function relating the probability of becoming pregnant as a function of body mass as common value for all individuals, we allowed for the possibility of genetic variation among individuals for this slope. We first estimated the relevant genetic parameters, and then built them into an expanded version of the model depicted in Figure 1b and Equation (7) (main text). We then integrated those estimates into an extended version of the selection model, and calculated the extended selection gradients of both the elevation (intercept), and the slope, of the reaction norm.

First, to quantify the genetic variance, we fitted a generalised random regression animal model of the form

$$\ln\left(\frac{p_{ik}}{1-p_{ik}}\right) = \alpha + \beta_m m_{ik} + \boldsymbol{\beta_d} f(d_{ik}, 2) + u_{mk} + a_{int_i} + a_{sl_i} \times m_{ik} + \epsilon_{ijk}, \qquad \text{(SI.4.1)}$$

a version of Equation (2) where variance in breeding values is estimated, associated with both the intercept (a_{int}) and the slope for body mass (a_{sl}). To assess selection of reaction norm parameters, we (1) evaluated the fitness of pregnant and non-pregnant females as a function of body mass and population density, thus obtaining *conditional individual fitness functions*, and we (2) calculated extended selection gradients for the intercept and slope of the logistic regression of the probability of pregnancy on mass. In (1) we were particularly interested in assessing whether the *conditional individual fitness functions* derived for pregnant and non-pregnant females would cross, as that would be a clear indication of variation in selection. In both analyses, we evaluated Equation (7) at fixed values of body mass and population density, setting the probability of pregnancy to 0 or 1 in analysis (1).

We found strong evidence of additive genetic variance in both the intercepts (2.04, 95% CrI 0.13; 9.19) and slopes (0.19, 95% CrI 0.05; 0.67) of the random regression being likely different from zero, confirming a genetic basis of the reaction norms (Tab. SI.4.1). Additionally, a positive additive genetic covariance between intercepts and slopes exists (0.61, 95% CrI 0.15; 2.35), suggesting that individuals that are genetically most predisposed to become pregnant are also those who's pregnancy status is most sensitive to their body mass.

Our extended selection gradient analysis found little evidence of variation in selection acting on the slope of the pregnancy-body mass reaction norm (Fig. SI.4.1). Most conditional individual fitness functions for

pregnant and non-pregnant females never cross, except for the lower end of naturally occurring population densities in this system (Fig. SI.4.1a,b,c), suggesting that the effect of body mass on fitness does not differ qualitatively between pregnant and non-pregnant females. Finally, we calculated extended selection gradients of the intercept and slope of the probablistic maturation reaction norm across sensible values of mean phenotype (body mass) and environmental conditions (population density). Estimates of η for the intercept are negative across almost the entire parameter space, while estimates of η for the slope of the reaction norm of early pregnancy on body mass are very close to zero (Fig. SI.4.1b). These results suggest that selection predominantly favours a decrease in the probability of early pregnancy, irrespective of population size and mean body mass. On the other hand, selection on the slope of the reaction norm is largely absent.

Table SI.4.1: Coefficients of a random regression animal model to estimate additive genetic reaction norms of early pregnancy on body mass in Soay female lambs. A binomial regression with logit link function was fit to the data, where variance in random cohorts was also estimated. All covariates were mean-centred. Values within brackets correspond to 95% highest posterior density credible intervals.

parameter	posterior mode	95% CrI
intercept	-0.88	(-1.63; -0.17)
body mass	0.74	(0.53; 0.98)
density ($\times$100)	-0.46	(-1.02; -0.08)
density^2 ($\times$100)	0.00	(0.00; 0.01)
variance in breeding values (intercepts):	2.04 (0.13; 9.19)	
variance in breeding values (slopes for mass):	0.19 (0.05; 0.67)	
covariance between breeding values (intercepts and slopes):	0.61 (0.15; 2.35)	
variance in cohort effects:	0.80 (0.19; 2.25)	

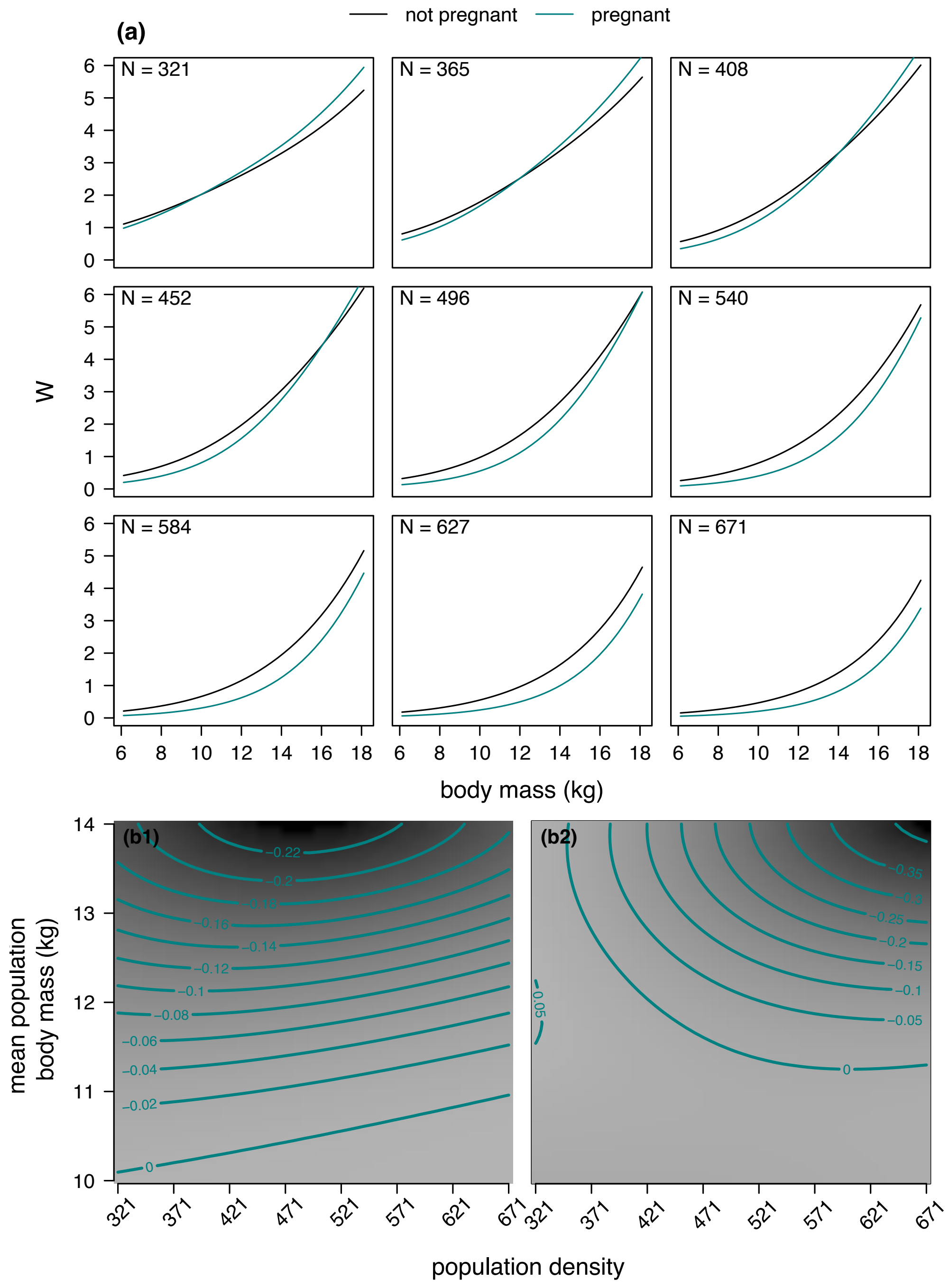

Figure SI.4.1: (a) Conditional individual fitness functions of (mean) body mass for pregnant and non-pregnant Soay sheep females and (b) selection gradients for the intercept (b1) and slope (b2) of early pregnancy on body mass as a function of population density and body mass. Note that if the effect of body mass on the strength of selection varies between pregnant and non-pregnant females the conditional individual fitness functions in (a) are expected to cross.

SI. 5 Details on solving derivatives and integrals

Estimating average trait values (Eq. (SI.3.3), main text) and variances (Eq. (SI.3.4), main text) on the expected scale implies solving integrals of high dimensionality in the context of the path analysis, and particularly when these equations are applied to the vector-valued function $\boldsymbol{f(\ell)}$ in equation (7). In such circumstances, solving integrals using Monte Carlo simulations is particularly useful as the error is independent from the integrand dimensionality, but mainly because other numerical methods are likely to fail. Monte Carlo integration relies on the fact that the integral $\int_L g(\ell)f(\ell)\mathrm{d}\ell$ (or $\int_L g^{-1}(\ell)f(\ell)\mathrm{d}\ell$ if applied to an inverse link function) corresponds to the expectation of g, $\mathbb{E}_f[g(L)]$. As a consequence, a random sample of latent values, L, generated using its density f can be used to approximate the empirical average of g,

$$\bar{g} \approx \frac{1}{n}\sum_{i=1}^{n} g(\ell_i), \tag{SI.5.1}$$

where n is the number of draws from L. $\bar{g}$ is also an approximation to $\int_L g(\ell)f(\ell)\mathrm{d}\ell$. This approximation converges to the real value of the integral by the law of large numbers as $n \to \infty$ Robert & Casella (2005). We sampled a large enough number of values from the distribution of the latent traits on which we evaluated $\boldsymbol{f(\ell)}$. Since the statistical models were fitted in a Bayesian framework, this procedure was applied to $n_{post} = 2000$ posterior independent samples.

For lower dimensional integrals, and particularly to obtain Φ (average derivative of the expected values with respect to the latent ones) in equation (SI.3.6) (main text), we used function *cuhre* from R package **cubature** that implemments a deterministic algorithm that uses cubature rules of polynomial degree, also allowing for multidimensional integration.

Likewise, the differentiation in the calculation of extended selection gradients (Eq. 10, main text) was accomplished by numerical linear approximation,

$$\mathrm{W}'(\ell) \approx \frac{\mathrm{W}(\ell + h) - \mathrm{W}(\ell)}{h}, \tag{SI.5.2}$$

where h is some small value. So, the extended selection gradient for mass would be obtained by evaluating W for a certain value of latent mass ℓ and at for $\ell + h$. This procedure was applied to $n_{MC} = 1000$ values from the distribution of the latent distributions on which we evaluated $\boldsymbol{f(\ell)}$ times $n_{post} = 2000$ posterior independent samples.

SI. 6 R code

6.1 Set up

6.1.1 R libraries

```
library(mvtnorm)
library(MCMCglmm)
library(QGglmm)
library(MASS)
library(xtable)
# library(MasterBayes) # not maintained at the time of
# submission
library(effects)
library(plyr)
library(QGglmm)
library(binom)
library(survival)
library(cubature)
library(plot3D)
```

6.1.2 Constants for analyses

```
n_dec = 2  #decimal places
last_year = 2015  # last year of data
n_samp = 2000  # number of posterior MCMC samples
n_MC = 1000  # number of Monte Carlo simulations for solving derivatives/integrals (see SI.5)
```

6.1.3 Colours for figures

```
teal = rgb(0, 128, 128, maxColorValue = 255)
grey_pal = grey.colors(n = 100, start = 0, end = 0.7, gamma = 2.2,
    alpha = NULL)
pal_cov = colorRampPalette(c(rgb(0, 200, 200, maxColorValue = 255),
    rgb(100, 0, 100, maxColorValue = 255)), space = "rgb")
```

6.1.4 Read data

```
load("data_ewes1.RData")  # all observations included
load("data_ewes2.RData")  # no missing observations
load("data_ewes3.RData")  # known first-year survival status
load("data_ewes4.RData")  # known SLReS
load("data_ewes_all_ages.RData")  # equivalent to data_ewes2 but including information for all ages
load("data_ped.RData")  # pedigree data
load("data_pop.RData")  # population data
sd_mass = sd(data_ewes4$MumWeight)
mu_mass = mean(data_ewes4$MumWeight)
sd_preg = sd(data_ewes4$pregnancy)
```

```
data_ewes2$mass_c = data_ewes2$mass - mean(data_ewes2$mass)
data_ewes2$measure_date_c = data_ewes2$measure_date - mean(data_ewes2$measure_date)
data_ewes2$birth_date_c = data_ewes2$birth_date - mean(data_ewes2$birth_date)
data_ewes2$mum_age_c = data_ewes2$mum_age - mean(data_ewes2$mum_age)
data_ewes2$density_c = data_ewes2$density - mean(data_ewes2$density)
```

6.2 Analyses

Code associated with the corresponding headings in the main text or Supporting Information (SI.2, SI.3, and SI.4).

6.2.1 Study system

Data collection

```
# number of individuals
n_ind1 = dim(data_ewes1)[1]
n_ind3 = dim(data_ewes3)[1]
n_ind4 = dim(data_ewes4)[1]
# age and longevity
age = data_ewes1$death_year - data_ewes1$cohort
age = age[!is.na(age)]
long_mean = mean(age)
long_max = max(age)
# statistics associated with the pedigree
n_ped = dim(data_ped)[1]
prop_ped_both = length(data_ped$ID[!is.na(data_ped$sireID) & !is.na(data_ped$mumID)])/n_ped
prop_ped_mum = length(data_ped$ID[is.na(data_ped$sireID) & !is.na(data_ped$mumID)])/n_ped
prop_ped_dad = length(data_ped$ID[!is.na(data_ped$sireID) & is.na(data_ped$mumID)])/n_ped
```

Summary statistics of body mass and early pregnancy

```
# number of pregnant lambs (914)
n_preg = sum(data_ewes1$pregnancy, na.rm = TRUE)
# proportion of lambs pregnant per year
prop_preg_year = tapply(data_ewes1$pregnancy, data_ewes1$cohort, mean)
```

6.2.2 Models of body mass, early pregnancy, survival and fitness

Twin status

```
prior = list(R = list(V = 1, nu = 1, fix = 1), G = list(G1 = list(V = 1,
    nu = 1, alpha.mu = 0, alpha.V = 1000), G2 = list(V = 1, nu = 1,
    alpha.mu = 0, alpha.V = 1000)))
mod_twin = MCMCglmm(twin ~ density_c + I(density_c^2) + mum_age_c +
    I(mum_age_c^2), random = ~mum_id + cohort, prior = prior, data = data_ewes2,
    nitt = 2e+06, burnin = 1500000, thin = 100, verbose = FALSE, family = "categorical")
mod_twin$Sol = mod_twin$Sol[3001:5000, ]
mod_twin$VCV = mod_twin$VCV[3001:5000, ]
mod_twin_sol = posterior.mode(as.mcmc(mod_twin$Sol))
mod_twin_sol_hpd = HPDinterval(as.mcmc(mod_twin$Sol))
mod_twin_vcv = posterior.mode(as.mcmc(mod_twin$VCV))
mod_twin_vcv_hpd = HPDinterval(as.mcmc(mod_twin$VCV))
```

Lamb body mass

```
prior = list(R = list(V = 1e-16, nu = -2), G = list(G1 = list(V = 1,
    nu = 1, alpha.mu = 0, alpha.V = 2000), G2 = list(V = 1, nu = 1,
    alpha.mu = 0, alpha.V = 2000), G3 = list(V = 1, nu = 1, alpha.mu = 0,
    alpha.V = 2000)))
mod_mass = MCMCglmm(mass ~ bith_date_c + measure_date_c + density_c +
    I(density_c^2) + mum_age_c + I(mum_age_c^2) + twin, random = ~animal +
    mum_id + cohort, prior = prior, data = data_ewes2, nitt = 1e+06,
    burnin = 5e+05, thin = 100, pedigree = data_ped, verbose = FALSE)
mod_mass$Sol = mod_mass$Sol[3001:5000, ]  # making sure to keep the last 2000 samples
mod_mass$VCV = mod_mass$VCV[3001:5000, ]  # making sure to keep the last 2000 samples
mod_mass_sol = posterior.mode(as.mcmc(mod_mass$Sol))
mod_mass_sol_hpd = HPDinterval(as.mcmc(mod_mass$Sol))
mod_mass_vcv = posterior.mode(as.mcmc(mod_mass$VCV))
mod_mass_vcv_hpd = HPDinterval(as.mcmc(mod_mass$VCV))
```

Early pregnancy

```
prior = list(R = list(V = 1, nu = 1, fix = 1), G = list(G1 = list(V = 1,
    nu = 1, alpha.mu = 0, alpha.V = 1000), G2 = list(V = 1, nu = 1,
    alpha.mu = 0, alpha.V = 1000)), B = list(mu = rep(0, 12), V = diag(12) *
    (1 + pi^2/3)))
mod_preg = MCMCglmm(pregnancy ~ mass_c + I(mass_c^2) + I(mass_c^3) +
    density_c + I(density_c^2) + mum_age_c + I(mum_age_c^2) + twin +
    mass_c:density_c + measure_date_c + birth_date_c, random = ~mum_id +
    cohort, family = "categorical", nitt = 2e+06, burnin = 1800000,
    thin = 50, prior = prior, data = data_ewes2, verbose = FALSE)
mod_preg$Sol = mod_preg$Sol[2001:4000, ]  # making sure to keep the last 2000 samples
mod_preg$VCV = mod_preg$VCV[2001:4000, ]  # making sure to keep the last 2000 samples
mod_preg_sol = apply(mod_preg$Sol, 2, function(x) posterior.mode(as.mcmc(x)))
mod_preg_vcv = apply(mod_preg$VCV, 2, function(x) posterior.mode(as.mcmc(x)))
mod_preg_sol_hpd = HPDinterval(as.mcmc(mod_preg$Sol))
mod_preg_vcv_hpd = HPDinterval(as.mcmc(mod_preg$VCV))
```

First-year survival

Proportion of birth before May 1.

```
birth_before_may = dim(data_ewes3[data_ewes3$birth_month < 5 & !is.na(data_ewes3$birth_month),
    ])[1]/dim(data_ewes3[!is.na(data_ewes3$birth_month), ])[1] * 100
```

Model as a function of body mass and pregnancy.

```
prior = list(R = list(V = 1, nu = 1, fix = 1), G = list(G1 = list(V = 1,
    nu = 1, alpha.mu = 0, alpha.V = 1000), G2 = list(V = 1, nu = 1,
    alpha.mu = 0, alpha.V = 1000)))
mod_surv = MCMCglmm(survival ~ mass_c * pregnancy * density_c + I(density_c^2) +
    mum_age_c + I(mum_age_c^2) + twin + birth_date_c + measure_date_c,
    random = ~mum_id + cohort, family = "categorical", nitt = 2e+06,
    burnin = 1600000, thin = 100, prior = prior, data = data_ewes3,
    verbose = FALSE)
# p-value for text
mod_surv_p_aux = summary(mod_surv)$solutions[3, 5]
if (mod_surv_p_aux < 1e-04) {
```

```
    mod_surv_p = "$<$ 0.0001"
} else if (mod_surv_p_aux < 0.001) {
    mod_surv_p = "$<$ 0.001"
} else if (mod_surv_p_aux < 0.01) {
    mod_surv_p = "$<$ 0.01"
} else if (mod_surv_p_aux < 0.05) {
    mod_surv_p = "$<$ 0.05"
} else mod_surv_p = "$>$ 0.05"
mod_surv$Sol = mod_surv$Sol[2001:4000, ]  # making sure to keep the last 2000 samples
mod_surv$VCV = mod_surv$VCV[2001:4000, ]  # making sure to keep the last 2000 samples
mod_surv_sol = posterior.mode(as.mcmc(mod_surv$Sol))
mod_surv_sol_hpd = HPDinterval(as.mcmc(mod_surv$Sol))
mod_surv_vcv = posterior.mode(as.mcmc(mod_surv$VCV))
mod_surv_vcv_hpd = HPDinterval(as.mcmc(mod_surv$VCV))
#### survival for averaged-sized females
prop_surv_notpreg_all = array(dim = n_samp)  ## survival among females who did not become pregnant
prop_surv_preg_all = array(dim = n_samp)  ## survival among females who did not become pregnant
for (i in 1:n_samp) {
    prop_surv_notpreg_all[i] = QGmean(mu = mod_surv$Sol[i, 1], var = mod_surv$VCV[i,
        1] + mod_surv$VCV[i, 3], link.inv = plogis)
    prop_surv_preg_all[i] = QGmean(mu = mod_surv$Sol[i, 1] + mod_surv$Sol[i,
        3], var = mod_surv$VCV[i, 1] + mod_surv$VCV[i, 3], link.inv = plogis)
}
prop_surv_notpreg = posterior.mode(as.mcmc(prop_surv_notpreg_all)) *
    100
prop_surv_preg = posterior.mode(as.mcmc(prop_surv_preg_all)) * 100
prop_surv_notpreg_hpd = HPDinterval(as.mcmc(prop_surv_notpreg_all)) *
    100
prop_surv_preg_hpd = HPDinterval(as.mcmc(prop_surv_preg_all)) * 100
```

Model as a function of body mass only.

```
prior = list(R = list(V = 1, n = 0, fix = 1), G = list(G1 = list(V = 1,
    nu = 1, alpha.mu = 0, alpha.V = 1000), G2 = list(V = 1, nu = 1,
    alpha.mu = 0, alpha.V = 1000)))
mod_surv_nopreg = MCMCglmm(survival ~ mass_c * density_c + I(density_c^2) +
    twin + mum_age_c + I(mum_age_c^2) + bith_date_c + measure_date_c,
    random = ~mum_id + cohort, family = "categorical", data = data_ewes3,
    prior = prior, nitt = 3e+06, burnin = 2800000, thin = 100)
# p-value for text
mod_surv_nopreg_p_aux = summary(mod_surv_nopreg)$solutions[2, 5]
if (mod_surv_nopreg_p_aux < 1e-04) {
    mod_surv_nopreg_p = "$<$ 0.0001"
} else if (mod_surv_nopreg_p_aux < 0.001) {
    mod_surv_nopreg_p = "$<$ 0.001"
} else if (mod_surv_nopreg_p_aux < 0.01) {
    mod_surv_nopreg_p = "$<$ 0.01"
} else if (mod_surv_nopreg_p_aux < 0.05) {
    mod_surv_nopreg_p = "$<$ 0.05"
} else mod_surv_nopreg_p = "$>$ 0.05"
mod_surv_nopreg_sol = posterior.mode(mod_surv_nopreg$Sol)
mod_surv_nopreg_sol_hpd = HPDinterval(mod_surv_nopreg$Sol)
mod_surv_nopreg_vcv = posterior.mode(mod_surv_nopreg$VCV)
mod_surv_nopreg_vcv_hpd = HPDinterval(mod_surv_nopreg$VCV)
```

Masses of pregnant and non-pregnant females.

```
# subset of pregnant females
d_p1 = data_ewes3[data_ewes3$pregnancy == 1, ]
# subset of pregnant and surviving females
d_p1s1 = d_p1[d_p1$survival == 1, ]
# subset of pregnant and surviving females with surviving
# offspring
d_p1s1o1 = d_p1s1[d_p1s1$fyres == 1 & !is.na(d_p1s1$fyres), ]
mod_mass_p1 = MCMCglmm(mass ~ measure_date_c + bith_date_c + density_c +
    I(density_c^2) + mum_age_c + I(mum_age_c^2) + twin, random = ~mum_id +
    cohort, data = d_p1, thin = 100, nitt = 5e+05, burnin = 3e+05)
mass_p1_sol = posterior.mode(mod_mass_p1$Sol)
mass_p1_sol_hpd = HPDinterval(mod_mass_p1$Sol)
mod_mass_p1s1 = MCMCglmm(mass ~ measure_date_c + bith_date_c + density_c +
    I(density_c^2) + mum_age_c + I(mum_age_c^2) + twin, random = ~mum_id +
    cohort, data = d_p1s1, thin = 100, nitt = 1e+06, burnin = 8e+05)
mass_p1s1_sol = posterior.mode(mod_mass_p1s1$Sol)
mass_p1s1_sol_hpd = HPDinterval(mod_mass_p1s1$Sol)
```

First-year rearing success (FYReS)

Number of surviving offspring (until November 1 and April 1).

```
n_surv_off_nov = as.numeric(table(data_ewes1$pregnancy[data_ewes1$surv_off_1Nov ==
    1]))
n_surv_off_apr = as.numeric(table(data_ewes1$pregnancy[data_ewes1$fyres ==
    1]))
```

Fitting the model.

```
prior = list(R = list(V = 1, fix = 1))
mod_fyres = MCMCglmm(fyres ~ mass_c + birth_date_c + measure_date_c +
    density_c + I(density_c^2), family = "categorical", data = data_ewes3[data_ewes3$pregnancy ==
    1 & data_ewes3$survival == 1 & !is.na(data_ewes3$surv_off_1Nov),
    ], prior = prior, nitt = 2e+06, burnin = 1800000, thin = 100)
mod_fyres_sol = posterior.mode(mod_fyres$Sol)
mod_fyres_vcv = posterior.mode(mod_fyres$VCV)
mod_fyres_sol_hpd = HPDinterval(mod_fyres$Sol)
mod_fyres_vcv_hpd = HPDinterval(mod_fyres$VCV)
prop_fyres_all = array(dim = n_samp)
for (i in 1:n_samp) prop_fyres_all[i] = QGmean(mu = mod_fyres$Sol[i,
    1], var = mod_fyres$VCV[i, 1], link.inv = plogis)
prop_fyres = posterior.mode(as.mcmc(prop_fyres_all)) * 100
prop_fyres_hpd = HPDinterval(as.mcmc(prop_fyres_all)) * 100
```

Subsequent lifetime rearing success (SLReS)

```
prior = list(R = list(V = 1, nu = 0.002), G = list(G1 = list(V = 1,
    nu = 1, alpha.mu = 0, alpha.V = 1000), G2 = list(V = 1, nu = 1,
    alpha.mu = 0, alpha.V = 1000)))
mod_slres = MCMCglmm(slres ~ mass_c * pregnancy * density_c + I(density_c^2) +
    twin + mum_age_c + I(mum_age_c^2) + measure_date_c + bith_date_c,
    random = ~mum_id + cohort, rcov = ~units, family = "poisson",
```

```
    data = data_ewes4[!is.na(data_ewes4$survival) & data_ewes4$survival ==
        1, ], nitt = 2e+05, prior = prior, burnin = 1e+05, thin = 50)
mod_slres_nopreg = MCMCglmm(slres ~ mass_c * density_c + I(density_c^2) +
    twin + mum_age_c + I(mum_age_c^2) + measure_date_c + bith_date_c,
    random = ~mum_id + cohort, rcov = ~units, family = "poisson",
    dsata = data_ewes4[!is.na(data_ewes4$survival) & data_ewes4$survival ==
        1, ], nitt = 2e+05, prior = prior, burnin = 1e+05, thin = 50)
mod_slres_nopreg_sol = posterior.mode(mod_slres_nopreg$Sol)
mod_slres_nopreg_sol_hpd = HPDinterval(mod_slres_nopreg$Sol)
mod_slres_nopreg_vcv = posterior.mode(mod_slres_nopreg$VCV)
mod_slres_nopreg_vcv_hpd = HPDinterval(mod_slres_nopreg$VCV)
mod_slres_sol = posterior.mode(mod_slres$Sol)
mod_slres_sol_hpd = HPDinterval(mod_slres$Sol)
mod_slres_vcv = posterior.mode(mod_slres$VCV)
mod_slres_vcv_hpd = HPDinterval(mod_slres$VCV)
```

6.2.3 Selection analysis

R function setting up the approach to solving integrals as detailed in Supplementary Information 5. This function is, therefore, used to calculate mean phenotype, $\bar{z}$, the variance-covariance matrix, $\mathbf{P}$, and extended selection gradients, η.

```
f_eta = function(eps, x, density_i) {
    eps_twin = eps[, 1, ] + x[1]
    eps_mass = eps[, 2, ] + x[2]
    eps_preg = eps[, 3, ] + x[3]
    eps_surv = eps[, 4, ] + x[4]
    eps_fyres = eps[, 5, ] + x[5]
    eps_slres = eps[, 6, ] + x[6]
    twin_i = plogis(mod_twin$Sol[, 1] + mod_twin$Sol[, 2] * rep(density_i,
        n_samp) + mod_twin$Sol[, 3] * rep(density_i, n_samp)^2 + eps_twin)
    mass_i = mod_mass$Sol[, 1] + mod_mass$Sol[, 8] * twin_i + mod_mass$Sol[,
        4] * rep(density_i, n_samp) + mod_mass$Sol[, 5] * rep(density_i,
        n_samp)^2 + eps_mass
    pregnancy_i = plogis(mod_preg$Sol[, 1] + mod_preg$Sol[, 2] * mass_i +
        mod_preg$Sol[, 3] * mass_i^2 + mod_preg$Sol[, 4] * mass_i^3 +
        mod_preg$Sol[, 9] * twin_i + mod_preg$Sol[, 5] * density_i +
        mod_preg$Sol[, 6] * density_i^2 + mod_preg$Sol[, 12] * mass_i *
        density_i + eps_preg)
    survival_i = plogis(mod_surv$Sol[, 1] + mod_surv$Sol[, 2] * mass_i +
        mod_surv$Sol[, 3] * pregnancy_i + mod_surv$Sol[, 11] * pregnancy_i *
        mass_i + mod_surv$Sol[, 8] * twin_i + mod_surv$Sol[, 4] *
        density_i + mod_surv$Sol[, 5] * density_i^2 + mod_surv$Sol[,
        12] * mass_i * density_i + mod_surv$Sol[, 13] * pregnancy_i *
        density_i + mod_surv$Sol[, 14] * mass_i * pregnancy_i * density_i +
        eps_surv)
    fyres_i = plogis(mod_fyres$Sol[, 1] + mod_fyres$Sol[, 2] * mass_i +
        eps_fyres)
    slres_i = exp(mod_slres_nopreg$Sol[, 1] + mod_slres_nopreg$Sol[,
        2] * mass_i + mod_slres_nopreg$Sol[, 5] * twin_i + mod_slres_nopreg$Sol[,
        3] * density_i + mod_slres_nopreg$Sol[, 4] * density_i^2 +
        mod_slres_nopreg$Sol[, 10] * mass_i * density_i + eps_slres)
```

```
    w_i = survival_i * ((pregnancy_i * fyres_i) + slres_i)
    return(array(c(twin_i, mass_i, pregnancy_i, survival_i, fyres_i,
        slres_i, w_i), dim = c(n_MC, n_samp, 7)))
}
```

Similar function to caculate β instead of η.

```
f_beta = function(eps, x, density_i) {
    eps_twin = eps[, 1, ] + x[1]
    eps_mass = eps[, 2, ] + x[2]
    eps_preg = eps[, 3, ] + x[3]
    eps_surv = eps[, 4, ] + x[4]
    eps_fyres = eps[, 5, ] + x[5]
    eps_slres = eps[, 6, ] + x[6]
    twin_i = plogis(mod_twin$Sol[, 1] + mod_twin$Sol[, 2] * density_i +
        mod_twin$Sol[, 3] * density_i^2 + eps_twin)
    mass_i = mod_mass$Sol[, 1] + mod_mass$Sol[, 8] * twin_i + mod_mass$Sol[,
        4] * density_i + mod_mass$Sol[, 5] * density_i^2 + eps_mass
    pregnancy_i = plogis(mod_preg$Sol[, 1] + mod_preg$Sol[, 9] * twin_i +
        mod_preg$Sol[, 5] * density_i + mod_preg$Sol[, 6] * density_i^2 +
        eps_preg)
    survival_i = plogis(mod_surv$Sol[, 1] + mod_surv$Sol[, 2] * mass_i +
        mod_surv$Sol[, 3] * pregnancy_i + mod_surv$Sol[, 11] * pregnancy_i *
        mass_i + mod_surv$Sol[, 8] * twin_i + mod_surv$Sol[, 4] *
        density_i + mod_surv$Sol[, 5] * density_i^2 + mod_surv$Sol[,
        12] * mass_i * density_i + mod_surv$Sol[, 13] * pregnancy_i *
        density_i + mod_surv$Sol[, 14] * mass_i * pregnancy_i * density_i +
        eps_surv)
    fyres_i = plogis(mod_fyres$Sol[, 1] + mod_fyres$Sol[, 2] * mass_i +
        eps_fyres)
    slres_i = exp(mod_slres_nopreg$Sol[, 1] + mod_slres_nopreg$Sol[,
        2] * mass_i + mod_slres_nopreg$Sol[, 5] * twin_i + mod_slres_nopreg$Sol[,
        3] * density_i + mod_slres_nopreg$Sol[, 4] * density_i^2 +
        mod_slres_nopreg$Sol[, 10] * mass_i * density_i + eps_slres)
    w_i <- survival_i * ((pregnancy_i * fyres_i) + slres_i)
    return(array(c(twin_i, mass_i, pregnancy_i, survival_i, fyres_i,
        slres_i, w_i), dim = c(n_MC, n_samp, 7)))
}
```

Using the approach in equation (SI.5.2), Supporting Information 5, to calculate mean phenotype, $\bar{z}$ (Eq. 8, main text) and selection gradients, η and β (Eq. 10, main text).

```
h = 0.2
hp = 0.3
n_dens = length(unique(data_ewes4$density))
uniquedensity = sort(unique(data_ewes4$density_c))
uniqueDensity = sort(unique(data_ewes4$density))
## solve (eq. 8) and (eq. 10) - see supplementary information 5
sigma_eps_all = list()
## note that it is not marginalising cohort so as to get an
## average cohort at a given density, instead of an average over
## cohorts at a given density (approach adopted throughout)
for (i in 1:n_samp) {
    sigma_eps_all[[i]] = diag(c(mod_twin$VCV[i, 1] + mod_twin$VCV[i,
```

```
        3], mod_mass$VCV[i, 1] + mod_mass$VCV[i, 2] + mod_mass$VCV[i,
        4], mod_preg$VCV[i, 1] + mod_preg$VCV[i, 3], mod_surv$VCV[i,
        1] + mod_surv$VCV[i, 3], mod_fyres$VCV[i, 1], mod_slres_nopreg$VCV[i,
        1] + mod_slres_nopreg$VCV[i, 3]))
}
eps = array(dim = c(n_MC, 6, n_samp))
for (i in 1:n_samp) eps[, , i] = rmvnorm(n_MC, rep(0, 6), sigma_eps_all[[i]])
## r objects mean phenotype
zbar_dens_all = array(dim = c(n_samp, 7, n_dens))
zbar_beta_dens_all = array(dim = c(n_samp, 7, n_dens))
# extended selection gradient (mass)
eta_mass_dens_all = array(dim = c(n_samp, n_dens))
# direct selection gradient (mass)
beta_mass_dens_all = array(dim = c(n_samp, n_dens))
# extended selection gradient (preg)
eta_preg_dens_all = array(dim = c(n_samp, n_dens))
# extended selection gradient (mass, W = surv)
eta_mass_Wsurv_dens_all = array(dim = c(n_samp, n_dens))
# direct selection gradient (mass, W = surv)
beta_mass_Wsurv_dens_all = array(dim = c(n_samp, n_dens))
for (k in 1:n_dens) {
    ## mean phenotype
    zbar_k_nMC = f_eta(eps, x = c(0, 0, 0, 0, 0, 0), density_i = uniquedensity[k])
    zbar_k = apply(zbar_k_nMC, c(2, 3), mean)
    zbar_dens_all[, , k] = zbar_k
    zbar_k_prime_nMC = f_eta(eps, x = c(0, h, 0, 0, 0, 0), density_i = uniquedensity[k])
    zbar_k_prime = apply(zbar_k_prime_nMC, c(2, 3), mean)
    zbar_k_prime_preg_nMC = f_eta(eps, x = c(0, 0, hp, 0, 0, 0), density_i = uniquedensity[k])
    zbar_k_prime_preg = apply(zbar_k_prime_preg_nMC, c(2, 3), mean)
    zbar_k_beta_nMC = f_beta(eps, x = c(0, 0, 0, 0, 0, 0), density_i = uniquedensity[k])
    zbar_k_beta = apply(zbar_k_beta_nMC, c(2, 3), mean)
    zbar_k_beta_prime_nMC = f_beta(eps, x = c(0, h, 0, 0, 0, 0), density_i = uniquedensity[k])
    zbar_k_beta_prime = apply(zbar_k_beta_prime_nMC, c(2, 3), mean)
    zbar_beta_dens_all[, , k] = zbar_k_beta
    ## extended and direct selection gradient for mass
    eta_mass_dens_all[, k] = (zbar_k_prime[, 7] - zbar_k[, 7])/h/zbar_k[,
        7]
    beta_mass_dens_all[, k] = (zbar_k_beta_prime[, 7] - zbar_k_beta[,
        7])/h/zbar_k_beta[, 7]
    ## extended selection gradient for pregnancy
    eta_preg_dens_all[, k] = (zbar_k_prime_preg[, 7] - zbar_k[, 7])/(zbar_k_prime_preg[,
        3] - zbar_k[, 3])/zbar_k[, 7]
    ## extended and direct selection gradient for mass, with
    ## fitness defined as first-year survival
    eta_mass_Wsurv_dens_all[, k] = (zbar_k_prime[, 4] - zbar_k[, 4])/h/zbar_k[,
        4]
    beta_mass_Wsurv_dens_all[, k] = (zbar_k_beta_prime[, 4] - zbar_k_beta[,
        4])/h/zbar_k_beta[, 4]
}
```

Summary statistics for mean phenotype.

```
zbar_all = apply(zbar_dens_all, c(1, 2), function(x) mean(as.mcmc(x)))
zbar = apply(zbar_all, 2, function(x) mean(as.mcmc(x)))
zbar_hpd = HPDinterval(as.mcmc(zbar_all))
zbar_mass_text = zbar[2] + mean(data_ewes1$MumWeight, na.rm = TRUE)
zbar_mass_text_hpd = zbar_hpd[2, ] + mean(data_ewes1$MumWeight, na.rm = TRUE)
zbar_dens = apply(zbar_dens_all, c(2, 3), function(x) mean(as.mcmc(x)))
zbar_dens_hpd = apply(zbar_dens_all, c(2, 3), function(x) HPDinterval(as.mcmc(x)))
```

Using the approach in equation (SI.5.2), Supporting Information 5, to calculate the variance-covariance matrix, **P**, (Eq. 9, main text).

```
vcov_ALL = array(dim = c(n_MC, n_samp, n_dens, 7, 7))
for (k in 1:n_dens) {
    zall = f_eta(eps, x = c(0, 0, 0, 0, 0, 0), density_i = uniquedensity[k])
    for (i in 1:n_samp) {
        for (j in 1:n_MC) {
            vcov_ALL[j, i, k, , ] = (zall[j, i, ] - zbar_dens_all[i,
                , k]) %*% t(zall[j, i, ] - zbar_dens_all[i, , k])
        }
    }
}
```

Summary statistics for the variance-covariance matrix.

```
vcov_dens_all = apply(vcov_ALL, c(4, 5, 2, 3), mean)
vcov_all = apply(vcov_dens_all, c(1, 2, 3), function(x) mean(x))
vcor_all = array(dim = c(7, 7, n_samp))
for (i in 1:n_samp) {
    vcor_all[, , i] = cov2cor(vcov_all[, , i])
}
```

```
vcov = apply(vcov_all, c(1, 2), function(x) posterior.mode(as.mcmc(x)))
vcor = apply(vcor_all, c(1, 2), function(x) posterior.mode(as.mcmc(x)))
vcov_all_95CrIU = vcov_all_95CrIL = matrix(ncol = 7, nrow = 7)
vcor_all_95CrIU = vcor_all_95CrIL = matrix(ncol = 7, nrow = 7)
for (i in 1:7) {
    for (j in 1:7) {
        vcov_all_95CrIU[i, j] = HPDinterval(as.mcmc(vcov_all[i, j,
            ]), prob = 0.95)[2]
        vcov_all_95CrIL[i, j] = HPDinterval(as.mcmc(vcov_all[i, j,
            ]), prob = 0.95)[1]
        vcor_all_95CrIU[i, j] = HPDinterval(as.mcmc(vcor_all[i, j,
            ]), prob = 0.95)[2]
        vcor_all_95CrIL[i, j] = HPDinterval(as.mcmc(vcor_all[i, j,
            ]), prob = 0.95)[1]
    }
}
```

Summary statistics for the extended selection gradient for mass.

```
eta_mass_all = apply(eta_mass_dens_all, 1, function(x) mean(as.mcmc(x)))
eta_mass = mean(eta_mass_all)
eta_mass_hpd = HPDinterval(as.mcmc(eta_mass_all))
```

```
eta_mass_sd = eta_mass * sd_mass
eta_mass_sd_hpd = eta_mass_hpd * sd_mass
eta_mass_dens = apply(eta_mass_dens_all, 2, function(x) mean(as.mcmc(x)))
eta_mass_dens_hpd = HPDinterval(as.mcmc(eta_mass_dens_all))
eta_mass_dens_sd = eta_mass_dens * sd_mass
eta_mass_dens_sd_hpd = eta_mass_dens_hpd * sd_mass
### for 3 different particular population densities
density_min = min(uniqueDensity)  #pos 1
density_med = median(uniqueDensity)  # pos 10
density_max = max(uniqueDensity)  # pos length(uniqueDensity)
eta_mass_d1 = eta_mass_dens[1]
eta_mass_d1_hpd = HPDinterval(as.mcmc(eta_mass_dens_all))[1, ]
eta_mass_d2 = eta_mass_dens[10]
eta_mass_d2_hpd = HPDinterval(as.mcmc(eta_mass_dens_all))[10, ]
eta_mass_d3 = eta_mass_dens[16]
eta_mass_d3_hpd = HPDinterval(as.mcmc(eta_mass_dens_all))[16, ]
eta_mass_d1_sd = eta_mass_d1 * sd_mass
eta_mass_d1_sd_hpd = eta_mass_d1_hpd * sd_mass
eta_mass_d2_sd = eta_mass_d2 * sd_mass
eta_mass_d2_sd_hpd = eta_mass_d2_hpd * sd_mass
eta_mass_d3_sd = eta_mass_d3 * sd_mass
eta_mass_d3_sd_hpd = eta_mass_d3_hpd * sd_mass
eta_mass_d1_mu = eta_mass_d1 * mu_mass
eta_mass_d1_mu_hpd = eta_mass_d1_hpd * mu_mass
eta_mass_d2_mu = eta_mass_d2 * mu_mass
eta_mass_d2_mu_hpd = eta_mass_d2_hpd * mu_mass
eta_mass_d3_mu = eta_mass_d3 * mu_mass
eta_mass_d3_mu_hpd = eta_mass_d3_hpd * mu_mass
```

Summary statistics for the extended selection gradient for early pregnanvy.

```
eta_preg_all = apply(eta_preg_dens_all, 1, function(x) mean(as.mcmc(x)))
eta_preg = mean(as.mcmc(eta_preg_all))
eta_preg_hpd = HPDinterval(as.mcmc(eta_preg_all))
eta_preg_sd = eta_preg * sd_preg
eta_preg_sd_hpd = eta_preg_hpd * sd_preg
eta_preg_dens = apply(eta_preg_dens_all, 2, function(x) mean(as.mcmc(x)))
eta_preg_dens_hpd = HPDinterval(as.mcmc(eta_preg_dens_all))
eta_preg_dens_sd = eta_preg_dens * sd_preg
eta_preg_dens_sd_hpd = eta_preg_dens_hpd * sd_preg
### for 3 different densities
eta_preg_d1 = eta_preg_dens[1]
eta_preg_d1_hpd = eta_preg_dens_hpd[1, ]
eta_preg_d2 = eta_preg_dens[10]
eta_preg_d2_hpd = eta_preg_dens_hpd[10, ]
eta_preg_d3 = eta_preg_dens[16]
eta_preg_d3_hpd = eta_preg_dens_hpd[16, ]
eta_preg_d1_sd = eta_preg_d1 * sd_preg
eta_preg_d1_sd_hpd = eta_preg_d1_hpd * sd_preg
eta_preg_d2_sd = eta_preg_d2 * sd_preg
eta_preg_d2_sd_hpd <- eta_preg_d2_hpd * sd_preg
eta_preg_d3_sd = eta_preg_d3 * sd_preg
eta_preg_d3_sd_hpd = eta_preg_d3_hpd * sd_preg
```

Summary statistics for the extended selection gradient for mass, defining fitness as first-year survival.

```
eta_mass_Wsurv_all = apply(eta_mass_Wsurv_dens_all, 1, function(x) mean(as.mcmc(x)))
eta_mass_Wsurv = mean(as.mcmc(eta_mass_Wsurv_all))  #used to be mean
eta_mass_Wsurv_hpd = HPDinterval(as.mcmc(eta_mass_Wsurv_all))
eta_mass_Wsurv_sd = eta_mass_Wsurv * sd_mass
eta_mass_Wsurv_sd_hpd = eta_mass_Wsurv_hpd * sd_mass
prop_mass_Wsurv_all = eta_mass_Wsurv_all/eta_mass_all
prop_mass_Wsurv = mean(as.mcmc(prop_mass_Wsurv_all))
prop_mass_Wsurv_hpd = HPDinterval(as.mcmc(prop_mass_Wsurv_all))
eta_mass_Wsurv_dens = apply(eta_mass_Wsurv_dens_all, 2, function(x) mean(as.mcmc(x)))
eta_mass_Wsurv_dens_hpd = HPDinterval(as.mcmc(eta_mass_Wsurv_dens_all))
eta_mass_Wsurv_dens_sd = eta_mass_Wsurv_dens * sd_mass
eta_mass_Wsurv_dens_sd_hpd = eta_mass_Wsurv_dens_hpd * sd_mass
# ### for 3 different densities
eta_mass_Wsurv_d1 = eta_mass_Wsurv_dens[1]
eta_mass_Wsurv_d1_hpd = eta_mass_Wsurv_dens_hpd[1, ]
eta_mass_Wsurv_d2 = eta_mass_Wsurv_dens[10]
eta_mass_Wsurv_d2_hpd = eta_mass_Wsurv_dens_hpd[10, ]
eta_mass_Wsurv_d3 = eta_mass_Wsurv_dens[16]
eta_mass_Wsurv_d3_hpd = eta_mass_Wsurv_dens_hpd[16, ]
eta_mass_Wsurv_d1_sd = eta_mass_Wsurv_d1 * sd_mass
eta_mass_Wsurv_d1_sd_hpd = eta_mass_Wsurv_d1_hpd * sd_mass
eta_mass_Wsurv_d2_sd = eta_mass_Wsurv_d2 * sd_mass
eta_mass_Wsurv_d2_sd_hpd = eta_mass_Wsurv_d2_hpd * sd_mass
eta_mass_Wsurv_d3_sd = eta_mass_Wsurv_d3 * sd_mass
eta_mass_Wsurv_d3_sd_hpd = eta_mass_Wsurv_d3_hpd * sd_mass
eta_mass_Wsurv_d1_mu = eta_mass_Wsurv_d1 * mu_mass
eta_mass_Wsurv_d1_mu_hpd = eta_mass_Wsurv_d1_hpd * mu_mass
eta_mass_Wsurv_d2_mu = eta_mass_Wsurv_d2 * mu_mass
eta_mass_Wsurv_d2_mu_hpd = eta_mass_Wsurv_d2_hpd * mu_mass
eta_mass_Wsurv_d3_mu = eta_mass_Wsurv_d3 * mu_mass
eta_mass_Wsurv_d3_mu_hpd = eta_mass_Wsurv_d3_hpd * mu_mass
```

Summary statistics for the direct selection gradient for mass.

```
beta_mass_all = apply(beta_mass_dens_all, 1, function(x) mean(as.mcmc(x)))
beta_mass = mean(as.mcmc(beta_mass_all))
beta_mass_hpd = HPDinterval(as.mcmc(beta_mass_all))
beta_mass_sd = beta_mass * sd_mass
beta_mass_sd_hpd = beta_mass_hpd * sd_mass
beta_mass_dens = apply(beta_mass_dens_all, 2, function(x) mean(as.mcmc(x)))
beta_mass_dens_hpd = apply(beta_mass_dens_all, 2, function(x) HPDinterval(as.mcmc(x)))
beta_mass_dens_sd = beta_mass_dens * sd_mass
beta_mass_dens_sd_hpd = beta_mass_dens_hpd * sd_mass
diff_beta_mass_all = beta_mass_all - eta_mass_all
diff_beta_mass = mean(as.mcmc(diff_beta_mass_all))
diff_beta_mass_hpd = HPDinterval(as.mcmc(diff_beta_mass_all))
```

Summary statistics for the direct selection gradient for mass, defining fitness as first-year survival.

```
beta_mass_Wsurv_all = apply(beta_mass_Wsurv_dens_all, 1, function(x) mean(as.mcmc(x)))
beta_mass_Wsurv = mean(as.mcmc(beta_mass_Wsurv_all))
beta_mass_Wsurv_hpd = HPDinterval(as.mcmc(beta_mass_Wsurv_all))
```

```
beta_mass_Wsurv_dens = apply(beta_mass_Wsurv_dens_all, 2, function(x) mean(as.mcmc(x)))
beta_mass_Wsurv_dens_hpd = HPDinterval(as.mcmc(beta_mass_Wsurv_dens_all))
```

6.3 Figures (main text)

6.3.1 Figure 2

Auxiliary code for figure 2c.

```
modPregFig1_prop_all = array(dim = n_samp)
for (i in 1:n_samp) modPregFig1_prop_all[i] = QGmean(mu = mod_preg$Sol[i,
    1], var = mod_preg$VCV[i, 1] + mod_preg$VCV[i, 2] + mod_preg$VCV[i,
    3], link.inv = plogis)
modPregFig1_prop = mean(modPregFig1_prop_all)
```

Auxiliary code for figure 2d.

```
masses = (30:180)/10
cmasses = (30:180)/10 - mean(data_ewes2$mass, na.rm = TRUE)
modpregsize_prop_mass_all = array(dim = c(n_samp, length(masses)))
for (i in 1:length(masses)) {
    for (j in 1:n_samp) {
        modpregsize_prop_mass_all[j, i] = QGmean(mu = mod_preg$Sol[j,
            1] + mod_preg$Sol[j, 2] * cmasses[i] + mod_preg$Sol[j,
            3] * cmasses[i]^2 + mod_preg$Sol[j, 4] * cmasses[i]^3,
            var = mod_preg$VCV[j, 1] + mod_preg$VCV[j, 2] + mod_preg$VCV[j,
                3], link.inv = plogis)
    }
}
modpregsize_prop_mass = apply(modpregsize_prop_mass_all, 2, mean)
```

```
data_ewes2$mass_cat = round(data_ewes2$mass, 0)
binomCI = binom.confint(sum(data_ewes2$pregnancy[data_ewes2$mass_cat ==
    3]), length(data_ewes2$pregnancy[data_ewes2$mass_cat == 3]), methods = "exact")[1,
    ]
for (i in 1:15) {
    binomCI = rbind(binomCI, binom.confint(sum(data_ewes2$pregnancy[data_ewes2$mass_cat ==
        i + 3]), length(data_ewes2$pregnancy[data_ewes2$mass_cat ==
        i + 3]), methods = "exact")[1, ])
}
```

Auxiliary code for figure 2e.

```
data_ewes2$cohort_c = data_ewes2$cohort - mean(data_ewes2$cohort)
modfig1d1_v2 = MCMCglmm(mass ~ cohort_c + measure_date_c + bith_date_c +
    density_c + I(density_c^2), nitt = 1e+05, burnin = 20000, thin = 50,
    data = data_ewes2, verbose = FALSE)
prior = list(R = list(V = 1, n = 0, fix = 1))
modfig1d2_v2 = MCMCglmm(pregnancy ~ cohort_c + density_c + I(density_c^2),
    family = "categorical", data = data_ewes2, nitt = 1e+06, burnin = 750000,
    thin = 50, rcov = ~units, prior = prior, verbose = FALSE)
```

```
modfig1d1_sol = posterior.mode(modfig1d1$Sol)
modfig1d1_vcv = posterior.mode(modfig1d1$VCV)
cyears = (1991:2015) - mean(data_ewes2$cohort)
modfig1d2_all = array(dim = c(n_samp, length(1991:2015)))
modfig1d2_U_all = array(dim = c(n_samp, length(1991:2015)))
modfig1d2_L_all = array(dim = c(n_samp, length(1991:2015)))
for (i in 1:length(1991:2015)) {
    for (j in 1:n_samp) {
        modfig1d2_all[j, i] = QGmean(mu = modfig1d2$Sol[j, 1] + modfig1d2$Sol[j,
            2] * cyears[i], var = modfig1d2$VCV[j, 1], link.inv = plogis)
        modfig1d2_U_all[j, i] = QGmean(mu = modfig1d2$Sol[j, 1] +
            modfig1d2$Sol[j, 2] * cyears[i] + modfig1d2$Sol[j, 3] *
            sd(data_ewes2$density_c) + modfig1d2$Sol[j, 4] * sd(data_ewes2$density_c)^2,
            var = modfig1d2$VCV[j, 1], link.inv = plogis)
        modfig1d2_L_all[j, i] = QGmean(mu = modfig1d2$Sol[j, 1] +
            modfig1d2$Sol[j, 2] * cyears[i] - modfig1d2$Sol[j, 3] *
            sd(data_ewes2$density_c) + modfig1d2$Sol[j, 4] * -sd(data_ewes2$density_c)^2,
            var = modfig1d2$VCV[j, 1], link.inv = plogis)
    }
}
modfig1d2_mean = apply(modfig1d2_all, 2, mean)
modfig1d2_meanL = apply(modfig1d2_L_all, 2, mean)
modfig1d2_meanU = apply(modfig1d2_U_all, 2, mean)
```

Auxiliary code for figure 2d.

```
data_ewes_all_ages$age_cat = 4
data_ewes_all_ages$age_cat[data_ewes_all_ages$age == 1] = 1
data_ewes_all_ages$age_cat[data_ewes_all_ages$age == 2] = 2
data_ewes_all_ages$age_cat[data_ewes_all_ages$age == 3 | data_ewes_all_ages$age ==
    4 | data_ewes_all_ages$age == 5] = 3
data_ewes_all_ages$age_cat = as.factor(data_ewes_all_ages$age_cat)
```

Code for figure 2.

```
pdf("ewes_Fig2.pdf", height = 7.25, width = 6)
par(mar = c(1.25, 4.7, 0.75, 0), xpd = TRUE, oma = c(3, 2, 2, 5))
layout(matrix(c(1, 1, 2, 2, 3, 3, 4, 5, 6, 6, 7, 8), 6, 2, byrow = TRUE),
    heights = c(0.75, 0.45, 0.25, 0.75, 0.25, 0.75))
# figure 2a
plot(1991:2015, tapply(data_ewes1$pregnancy, data_ewes1$cohort, length)[7:31],
    type = "l", lwd = 2, col = 0, las = 1, ylim = c(0, 320), xlim = c(1991,
        2015), xlab = "", ylab = "number of female lambs", frame = FALSE,
    xaxs = "i", xaxt = "n")
polygon(c(1991:2015, rev(1991:2015)), c(tapply(data_ewes1$pregnancy,
    data_ewes1$cohort, length), rev(tapply(data_ewes1$pregnancy, data_ewes1$cohort,
    sum))), col = "grey", border = NA)
polygon(c(1991:2015, rev(1991:2015)), c(tapply(data_ewes1$pregnancy,
    data_ewes1$cohort, sum), rev(rep(0, 25))), col = teal, border = NA)
par(new = TRUE)
plot(1991:2015, data_pop$density[7:31], ylim = c(-100, 800), type = "l",
    axes = FALSE, ylab = "", xlab = "", col = 1, lty = 3, lwd = 2,
    xaxs = "i", xlim = c(1991, 2015))
axis(side = 4, seq(0, 800, by = 200), las = 1)
```

```
text(2018.25, 400, "population density", srt = 270, xpd = NA)
legend("topleft", c("pregnant lambs", "total ewe lambs", "population size"),
    bty = "n", lwd = 2, fill = c(teal, "grey", NA), lty = c(0, 0,
        3), col = c(teal, "grey", 1), border = c(0), x.intersp = c(0,
        0, 1.5), cex = 0.8)
for (i in 1991:2015) segments(x0 = i, x1 = i, y0 = -100, y1 = 800,
    col = rgb(0.5, 0.5, 0.5, 0.2))
text(1991.5, 855, expression(bold("(a)")))
# figure 2b
n_surv_yr_preg = tapply(data_ewes1$survival[data_ewes1$pregnancy ==
    1], data_ewes1$cohort[data_ewes1$pregnancy == 1], sum, na.rm = TRUE)
n_preg_yr = tapply(data_ewes1$pregnancy, data_ewes1$cohort, sum)
plot(1991:2015, n_surv_yr_preg/n_preg_yr, type = "l", las = 1, col = teal,
    ylab = "survival among \n pregnant females", frame = FALSE, xaxt = "n",
    xlab = "year", ylim = c(0, 0.5), yaxs = "i", xaxs = "i")
axis(1, at = seq(1991, 2015, by = 3))
for (i in 1991:2015) segments(x0 = i, x1 = i, y0 = 0, y1 = 1, col = rgb(0.5,
    0.5, 0.5, 0.2))
mtext(1, line = 2.5, text = "year", cex = 0.8)
text(1991.5, 0.48, expression(bold("(b)")))
# space
plot.new()
# fig 2c
wg0 = length(data_ewes2$mass[data_ewes2$pregnancy == 0])
wg1 = length(data_ewes2$mass[data_ewes2$pregnancy == 1])
plot(0, 1, ylim = c(0, 0.13), type = "l", las = 1, col = 0, xlab = "body mass (kg)",
    ylab = "probability density", main = "", xlim = c(3, 18), xaxs = "i",
    yaxs = "i")
polygon(density(data_ewes2$mass[data_ewes2$pregnancy == 0], weights = rep((1 -
    modPregFig1_prop)/wg0, wg0)), col = "grey", border = 0)
polygon(density(data_ewes2$mass[data_ewes2$pregnancy == 1], weights = rep(modPregFig1_prop/wg1,
    wg1)), col = rgb(0, 128, 128, 160, maxColorValue = 255), border = NA)
legend("topright", c("pregnant", "not pregnant"), fill = c(rgb(0,
    128, 128, 160, maxColorValue = 255), "grey"), border = c(0, 0),
    bty = "n", cex = 0.8)
text(3.75, 0.123, expression(bold("(c)")))
#####
# 2d
plot(masses, modpregsize_prop_mass, lwd = 2, col = teal, xlab = "body mass (kg)",
    ylab = "probability of early pregnancy", las = 1, lty = 1, ylim = c(0,
        1), cex = 0.2, type = "l", xlim = c(3, 18), xaxt = "n")
axis(side = 1, seq(4, 18, by = 2))
points(c(3:18), binomCI$mean, pch = 15, col = "grey")
segments(x0 = c(3:18), y0 = binomCI$lower, x1 = c(3:18), y1 = binomCI$upper,
    col = "grey")
text(3.5, 0.98, expression(bold("(d)")))
#####
# space
plot.new()
mtext(side = 3, text = "body mass (kg)", cex = 0.8, line = -2)
# (d) pregnancy and mass as a function of time !! check
plot(1991:2015, modfig1d1_sol[1] + modfig1d1_sol[2] * cyears, type = "l",
    ylim = c(8, 14), las = 1, ylab = "", xlab = "year", yaxt = "n")
```

```
polygon(c(1991:2015, rev(1991:2015)), c(modfig1d1_sol[1] + modfig1d1_sol[2] *
    cyears + modfig1d1_sol[5] * sd(data_ewes2$density_c) + modfig1d1_sol[6] *
    sd(data_ewes2$density_c)^2, rev(modfig1d1_sol[1] + modfig1d1_sol[2] *
    cyears + modfig1d1_sol[5] * (-sd(data_ewes2$density_c)) + modfig1d1_sol[6] *
    (-sd(data_ewes2$density_c^2)))), col = "grey90", border = NA)
lines(1991:2015, modfig1d1_sol[1] + modfig1d1_sol[2] * cyears)
axis(2, at = c(12, 13, 14), las = 1)
segments(y0 = 11, y1 = 11, x0 = 1990.1, x1 = 2015.9)
text(1984, 12.5, "mean body \nmass (kg)", srt = 90)
text(1991.5, 13.75, expression(bold("(e)")))
par(new = TRUE)
plot(1991:2015, modfig1d2_mean, yaxt = "n", type = "l", ylim = c(0.3,
    0.82), ylab = "", xlab = "", xaxt = "n")
polygon(c(1991:2015, rev(1991:2015)), c(modfig1d2_meanL, rev(modfig1d2_meanU)),
    col = "grey90", border = NA, axis = F)
lines(1991:2015, modfig1d2_mean)
axis(2, at = c(0.3, 0.4, 0.5), las = 1)
# axis(1, seq(from = 1991, to = 2015, by = 5), str = 35)
mtext(1, line = 2.5, text = "year", cex = 0.8)
text(1984, 0.42, "probability \nof pregnancy", srt = 90)
#####
# (e) survival curves
data_ewes_all_ages$time2 = data_ewes_all_ages$time + 0.5
plot(survfit(Surv(log(time2), cens) ~ 1, data = data_ewes_all_ages[data_ewes_all_ages$age_cat ==
    1, ]), conf.int = FALSE, col = 0, lwd = 2, las = 1, ylab = "survival probability",
    xlab = "log(years of life)", xlim = c(-1.5, 2.9), ylim = c(0,
        1), yaxs = "i", xaxs = "i")
polygon(c(c(-1.5, log(0.5)), c(log(0.5), -1.5)), c(c(1, 1), c(0, 0)),
    col = rgb(0, 128, 128, 100, maxColorValue = 255), border = NA)
lines(survfit(Surv(log(time2), cens) ~ 1, data = data_ewes_all_ages[data_ewes_all_ages$age_cat ==
    4, ]), conf.int = FALSE, col = "grey80", lwd = 2)
lines(survfit(Surv(log(time2), cens) ~ 1, data = data_ewes_all_ages[data_ewes_all_ages$age_cat ==
    3, ]), conf.int = FALSE, col = "grey60", lwd = 2)
lines(survfit(Surv(log(time2), cens) ~ 1, data = data_ewes_all_ages[data_ewes_all_ages$age_cat ==
    2, ]), conf.int = FALSE, col = "grey40", lwd = 2)
lines(survfit(Surv(log(time2), cens) ~ 1, data = data_ewes_all_ages[data_ewes_all_ages$age_cat ==
    1, ]), conf.int = FALSE, col = "grey20", lwd = 2)
segments(x0 = -1.5, x1 = log(0.5), y0 = 1, y1 = 1, col = "grey20",
    lwd = 2)
segments(x0 = log(0.5), x1 = log(0.5), y0 = 0.261, y1 = 1, col = "grey20",
    lwd = 2)
legend("topright", c("lambs", "yearlings", "2 to 4 year olds", "5 years and older"),
    lty = 1, lwd = 2, col = c("grey0", "grey40", "grey60", "grey80",
        "grey90"), bty = "n", cex = 0.8)
text(-1.275, 0.94, expression(bold("(f)")))
text(-1.1, 0.5, "gestation", srt = 90, col = "grey20")
text(1.1, 0.5, "p < 0.0001")
mtext(1, line = 2.5, text = "ln(years of life)", cex = 0.8)
dev.off()
```

6.3.2 Figure 3

Auxiliary code for figure 3a.

```
masses = (30:180)/10
cmasses = (30:180)/10 - mean(data_ewes3$mass, na.rm = TRUE)
# a
modsurvsize_prop_mass_i = array(dim = c(n_samp, length(masses)))
for (i in 1:n_samp) {
    for (j in 1:length(masses)) {
        modsurvsize_prop_mass_i[i, j] = QGmean(mu = mod_surv_nopreg$Sol[i,
            1] + mod_surv_nopreg$Sol[i, 2] * cmasses[j], var = mod_surv_nopreg$VCV[i,
            1] + mod_surv_nopreg$VCV[i, 2] + mod_surv_nopreg$VCV[i,
            3], link.inv = plogis)
    }
}
modsurvsize_prop_mass = apply(modsurvsize_prop_mass_i, 2, mean)
```

Auxiliary code for figure 3c.

```
modsurvsizenopreg_prop_mass_i = array(dim = c(n_samp, length(masses)))
modsurvsizepreg_prop_mass_i = array(dim = c(n_samp, length(masses)))
for (i in 1:n_samp) {
    for (j in 1:length(masses)) {
        modsurvsizenopreg_prop_mass_i[i, j] = QGmean(mu = mod_surv$Sol[i,
            1] + mod_surv$Sol[i, 2] * cmasses[j], var = mod_surv$VCV[i,
            1] + mod_surv$VCV[i, 2] + mod_surv$VCV[i, 3], link.inv = plogis)
        modsurvsizepreg_prop_mass_i[i, j] = QGmean(mu = mod_surv$Sol[i,
            1] + mod_surv$Sol[i, 3] + mod_surv$Sol[i, 2] * cmasses[j] +
            mod_surv$Sol[i, 11] * cmasses[j], var = mod_surv$VCV[i,
            1] + mod_surv$VCV[i, 2] + mod_surv$VCV[i, 3], link.inv = plogis)
    }
}
modsurvsizenopreg_prop_mass = apply(modsurvsizenopreg_prop_mass_i,
    2, mean)
modsurvsizepreg_prop_mass = apply(modsurvsizepreg_prop_mass_i, 2,
    mean)
```

Auxiliary code for figure 3b.

```
masses = (30:180)/10
cmasses = (30:180)/10 - mean(data_ewes4$MumWeight, na.rm = TRUE)
n_slres_no_preg_mass_all = array(dim = c(n_samp, length(masses)))
for (i in 1:n_samp) {
    for (j in 1:length(masses)) {
        n_slres_no_preg_mass_all[i, j] = QGmean(mu = mod_slres_nopreg$Sol[i,
            1] + mod_slres_nopreg$Sol[i, 2] * cmasses[j], var = mod_slres_nopreg$VCV[i,
            1] + mod_slres_nopreg$VCV[i, 2] + mod_slres_nopreg$VCV[i,
            3], link.inv = exp)
    }
}
n_slres_no_preg_mass = apply(n_slres_no_preg_mass_all, 2, mean)
```

Auxiliary code for figure 3d.

```
prior = list(R = list(V = 1, nu = 0.002), G = list(G1 = list(V = 1,
    nu = 1, alpha.mu = 0, alpha.V = 1000), G2 = list(V = 1, nu = 1,
    alpha.mu = 0, alpha.V = 1000)))
```

```
mod_slres_fig3 = MCMCglmm(slres ~ mass_c + pregnancy + density_c +
    mass_c:pregnancy + I(density_c^2) + twin + mum_age_c + I(mum_age_c^2) +
    measure_date_c + bith_date_c, random = ~mum_id + cohort, rcov = ~units,
    family = "poisson", data = sheepms4[!is.na(sheepms4$survival) &
        sheepms4$survival == 1, ], nitt = 2e+05, prior = prior, burnin = 1e+05,
    thin = 50)
```

```
n_slres_not_preg_mass_all = array(dim = c(n_samp, length(masses)))
n_slres_preg_mass_all = array(dim = c(n_samp, length(masses)))
for (i in 1:n_samp) {
    for (j in 1:length(masses)) {
        n_slres_not_preg_mass_all[i, j] = QGmean(mu = mod_slres_fig3$Sol[i,
            1] + mod_slres_fig3$Sol[i, 2] * cmasses[j], var = mod_slres_fig3$VCV[i,
            1] + mod_slres_fig3$VCV[i, 2] + mod_slres_fig3$VCV[i,
            3], link.inv = exp)
        n_slres_preg_mass_all[i, j] = QGmean(mu = mod_slres_fig3$Sol[i,
            1] + mod_slres_fig3$Sol[i, 3] + mod_slres_fig3$Sol[i,
            2] * cmasses[j] + mod_slres_fig3$Sol[i, 11] * cmasses[j],
            var = mod_slres_fig3$VCV[i, 1] + mod_slres_fig3$VCV[i,
                2] + mod_slres_fig3$VCV[i, 3], link.inv = exp)
    }
}
n_slres_not_preg_mass = apply(n_slres_not_preg_mass_all, 2, mean)
n_slres_preg_mass = apply(n_slres_preg_mass_all, 2, mean)
```

Code for figure 3.

```
pdf("ewes_Fig3.pdf", height = 5, width = 6)
par(mfrow = c(2, 2), xpd = TRUE, mar = c(1, 3, 0, 2), oma = c(4, 3,
    4, 0))
#--a
plot(data_ewes3$mass, data_ewes3$survival, pch = 16, col = 1, ylab = "",
    xlab = "", las = 1, xaxt = "n")
lines(masses, modsurvsize_prop_mass, col = 1, lwd = 2)
text(3.5, 0.97, expression(bold("(a)")))
legend(12, 1.25, c("all", "pregnant", "not pregnant"), lty = 1, col = c(1,
    teal, "grey"), bty = "n", cex = 0.8, horiz = TRUE, inset = c(-1,
    0), xpd = NA, lwd = 2)
#---b
plot(data_ewes4$mass[!is.na(data_ewes4$survival) & data_ewes4$survival ==
    1], data_ewes4$slres[!is.na(data_ewes4$survival) & data_ewes4$survival ==
    1], pch = 16, las = 1, ylab = "", xlab = "", xaxt = "n", col = 1,
    ylim = c(0, 13), xlim = c(3, 18))
lines(masses, n_slres_no_preg_mass, lwd = 2, col = 1)
text(3.5, 12.5, expression(bold("(b)")))
#--c
plot(data_ewes3$mass[data_ewes3$pregnancy == 0], data_ewes3$survival[data_ewes3$pregnancy ==
    0], pch = 16, col = "grey", ylab = "probability of first-year survival",
    xlab = "", las = 1, xaxt = "n")
axis(1, at = seq(4, 16, by = 4))
points(data_ewes3$mass[data_ewes3$pregnancy == 1], data_ewes3$survival[data_ewes3$pregnancy ==
    1], pch = 16, col = teal)
lines(masses, modsurvsizenopreg_prop_mass, col = "grey", lwd = 2)
```

```
lines(masses, modsurvsizepreg_prop_mass, col = teal, lwd = 2)
text(3.5, 0.97, expression(bold("(c)")))
mtext("probability of first-year survival", 2, line = 3, cex = 0.8,
    at = 1.1)
#---d
plot(data_ewes4$mass[data_ewes4$pregnancy == 0 & !is.na(data_ewes4$survival) &
    data_ewes4$survival == 1], data_ewes4$slres[data_ewes4$pregnancy ==
    0 & !is.na(data_ewes4$survival) & data_ewes4$survival == 1], pch = 16,
    col = "grey", las = 1, ylab = "", xlab = "", xaxt = "n", ylim = c(0,
        13), xlim = c(3, 18), cex.lab = 1.2)
points(data_ewes4$mass[data_ewes4$pregnancy == 1 & !is.na(data_ewes4$survival) &
    data_ewes4$survival == 1], data_ewes4$slres[data_ewes4$pregnancy ==
    1 & !is.na(data_ewes4$survival) & data_ewes4$survival == 1], col = teal,
    pch = 16)
lines(masses, n_slres_not_preg_mass, lwd = 2, col = "grey")
lines(masses, n_slres_preg_mass, lwd = 2, col = teal)
axis(1, at = seq(4, 16, by = 4))
text(3.5, 12.5, expression(bold("(d)")))
mtext("SLReS", side = 2, line = 3, cex = 0.8, at = 14)
mtext("body mass (kg)", 1, line = 3, cex = 0.9, at = -1)
dev.off()
```

6.3.3 Figure 4

Code for figure 4.

```
pdf("ewes_Fig4.pdf", height = 6, width = 5)
layout(matrix(c(1, 2, 3, 4, 5, 5, 5, 5, 6, 6, 6, 6, 7, 7, 7, 7), ncol = 4,
    byrow = TRUE), heights = c(0.5, 1, 1, 1))
par(xpd = TRUE, oma = c(4.5, 4, 0, 5), mar = c(0, 0.5, 1, 0.5))
# twinning
plot(uniqueDensity, zbar_dens[1, ], xaxt = "n", xlab = "", type = "l",
    lwd = 2, ylim = c(0, 1), col = teal, las = 1, main = "t")
polygon(c(uniqueDensity, rev(uniqueDensity)), c(zbar_dens_hpd[1, 1,
    ], rev(zbar_dens_hpd[2, 1, ])), col = rgb(0, 128, 128, 100, maxColorValue = 255),
    border = NA)
lines(uniqueDensity, zbar_dens[1, ], lwd = 2, col = teal)
mtext("probability", 2, line = 3, cex = 0.8)
par(new = TRUE)
hist(uniqueDensity, col = rgb(0.33, 0.33, 0.33, 0.2), add = FALSE,
    ylim = c(0, 5), main = "", yaxt = "n", border = rgb(0.33, 0.33,
        0.33, 0.2), xaxt = "n")
# pregnancy
plot(uniqueDensity, zbar_dens[3, ], xaxt = "n", xlab = "", ylab = "",
    type = "l", lwd = 2, ylim = c(0, 1), col = teal, yaxt = "n", main = "p")
polygon(c(uniqueDensity, rev(uniqueDensity)), c(zbar_dens_hpd[1, 3,
    ], rev(zbar_dens_hpd[2, 3, ])), col = rgb(0, 128, 128, 100, maxColorValue = 255),
    border = NA)
lines(uniqueDensity, zbar_dens[3, ], lwd = 2, col = teal)
par(new = TRUE)
hist(uniqueDensity, col = rgb(0.33, 0.33, 0.33, 0.2), add = FALSE,
    ylim = c(0, 5), main = "", yaxt = "n", border = rgb(0.33, 0.33,
        0.33, 0.2), xaxt = "n")
```

```
# survival
plot(uniqueDensity, zbar_dens[4, ], xaxt = "n", xlab = "", ylab = "",
    type = "l", lwd = 2, ylim = c(0, 1), col = teal, yaxt = "n", main = "s")
polygon(c(uniqueDensity, rev(uniqueDensity)), c(zbar_dens_hpd[1, 4,
    ], rev(zbar_dens_hpd[2, 4, ])), col = rgb(0, 128, 128, 100, maxColorValue = 255),
    border = NA)
lines(uniqueDensity, zbar_dens[4, ], lwd = 2, col = teal)
par(new = TRUE)
hist(uniqueDensity, col = rgb(0.33, 0.33, 0.33, 0.2), add = FALSE,
    ylim = c(0, 5), main = "", yaxt = "n", border = rgb(0.33, 0.33,
        0.33, 0.2), xaxt = "n")
# mass
plot(uniqueDensity, zbar_dens[2, ], xaxt = "n", xlab = "", ylab = "",
    type = "l", lwd = 2, col = teal, yaxt = "n", main = "m", ylim = c(-1,
        2))
axis(side = 4, seq(-1, 2, by = 1), las = 1)
polygon(c(uniqueDensity, rev(uniqueDensity)), c(zbar_dens_hpd[1, 2,
    ], rev(zbar_dens_hpd[2, 2, ])), col = rgb(0, 128, 128, 100, maxColorValue = 255),
    border = NA)
lines(uniqueDensity, zbar_dens[2, ], lwd = 2, col = teal)
text(890, 0.43, labels = "body \nmass (kg)", xpd = NA, srt = -90,
    cex = 1.2)
par(new = T)
hist(uniqueDensity, col = rgb(0.33, 0.33, 0.33, 0.2), add = FALSE,
    ylim = c(0, 5), main = "", yaxt = "n", border = rgb(0.33, 0.33,
        0.33, 0.2), xaxt = "n")
# fitness
plot(uniqueDensity, zbar_dens[7, ], xlab = "", ylab = "", las = 1,
    col = teal, type = "l", lwd = 2, xaxt = "n", ylim = c(0, 5))
mtext(expression(paste(bar(W))), 2, line = 3, cex = 0.8)
polygon(c(uniqueDensity, rev(uniqueDensity)), c(zbar_dens_hpd[1, 7,
    ], rev(zbar_dens_hpd[2, 7, ])), col = rgb(0, 128, 128, 100, maxColorValue = 255),
    border = NA)
lines(uniqueDensity, zbar_dens[7, ], lwd = 2, col = teal)
par(new = TRUE)
hist(uniqueDensity, col = rgb(0.33, 0.33, 0.33, 0.2), add = FALSE,
    ylim = c(0, 5), main = "", yaxt = "n", border = rgb(0.33, 0.33,
        0.33, 0.2), xaxt = "n")
# eta for mass
plot(uniqueDensity, eta_mass_dens, xlab = "", ylab = "", las = 1,
    col = teal, type = "l", lwd = 2, xaxt = "n", ylim = c(0.1, 0.55))
polygon(c(uniqueDensity, rev(uniqueDensity)), c(eta_mass_dens_hpd[,
    1], rev(eta_mass_dens_hpd[, 2])), col = rgb(0, 128, 128, 100,
    maxColorValue = 255), border = NA)
lines(uniqueDensity, eta_mass_dens, lwd = 2, col = teal)
# beta for mass
polygon(c(uniqueDensity, rev(uniqueDensity)), c(beta_mass_dens_hpd[1,
    ], rev(beta_mass_dens_hpd[2, ])), col = adjustcolor(pal_cov(3)[3],
    alpha.f = 0.5), border = NA)
lines(uniqueDensity, beta_mass_dens, lwd = 2, col = adjustcolor(pal_cov(3)[3],
    alpha.f = 0.5))
mtext(expression(paste(eta[m], "/", beta[m])), 2, line = 3, cex = 0.8)
par(new = TRUE)
```

```
hist(uniqueDensity, col = rgb(0.33, 0.33, 0.33, 0.2), add = FALSE,
    ylim = c(0, 5), main = "", yaxt = "n", border = rgb(0.33, 0.33,
        0.33, 0.2), xaxt = "n")
legend("topleft", c(expression(paste(eta[m])), expression(paste(beta[m]))),
    fill = c(rgb(0, 128, 128, 100, maxColorValue = 255), adjustcolor(pal_cov(3)[3],
        alpha.f = 0.5)), bty = "n", border = 0)
plot(uniqueDensity, eta_preg_dens, xlab = "", ylab = "", las = 1,
    col = teal, type = "l", lwd = 2, ylim = c(-2.5, 0.5))
polygon(c(uniqueDensity, rev(uniqueDensity)), c(eta_preg_dens_hpd[,
    1], rev(eta_preg_dens_hpd[, 2])), col = rgb(0, 128, 128, 100,
    maxColorValue = 255), border = NA)
lines(uniqueDensity, eta_preg_dens, lwd = 2, col = teal)
mtext(expression(paste(eta[p])), 2, line = 3, cex = 0.8)
mtext("population density", 1, line = 3, cex = 0.8)
par(new = TRUE)
hist(uniqueDensity, col = rgb(0.33, 0.33, 0.33, 0.2), add = FALSE,
    ylim = c(0, 5), main = "", yaxt = "n", border = rgb(0.33, 0.33,
        0.33, 0.2), xaxt = "n", ylab = "")
dev.off()
```

6.3.4 Figure 5

Code for figure 5.

```
pdf("ewes_Fig5.pdf", height = 5, width = 5)
par(mar = c(4, 6, 4, 4))
plot(uniqueDensity, eta_mass_Wsurv_dens/eta_mass_dens, xlab = "population density",
    ylab = "proportion of selection \non lamb body mass", las = 1,
    col = teal, type = "l", lwd = 2, ylim = c(0, 0.7), cex.lab = 0.9,
    cex.axis = 0.9)
lines(uniqueDensity, beta_mass_Wsurv_dens/beta_mass_dens, lwd = 2,
    col = teal, lty = 2)
par(new = TRUE)
hist(uniqueDensity, col = rgb(0.33, 0.33, 0.33, 0.2), add = FALSE,
    ylim = c(0, 5), main = "", yaxt = "n", xaxt = "n", border = rgb(0.33,
        0.33, 0.33, 0.2), xlab = "", ylab = "")
legend("topleft", c(expression(paste(eta[m])), expression(paste(beta[m]))),
    bty = "n", lwd = 2, lty = c(1, 2), col = teal)
dev.off()
```

6.4 Supporting Information 2 - supplementary figures

Auxiliary code for figure SI.2.1.

```
cdens2 = seq(-162, 188, by = 2)
dens2 = seq(321, 671, by = 2)
n_dens2 = length(dens2)
```

```
modsurvsizenopreg_prop_mass_ijk = array(dim = c(n_samp, length(masses),
    n_dens2))
modsurvsizepreg_prop_mass_ijk = array(dim = c(n_samp, length(masses),
```

```
    n_dens2))
for (i in 1:n_samp) {
    for (k in 1:n_dens2) {
        for (j in 1:length(masses)) {
            modsurvsizenopreg_prop_mass_ijk[i, j, k] = QGmean(mu = mod_surv$Sol[i,
                1] + mod_surv$Sol[i, 2] * cmasses[j] + mod_surv$Sol[i,
                4] * cdens2[k] + mod_surv$Sol[i, 5] * cdens2[k]^2 +
                mod_surv$Sol[i, 12] * cmasses[j] * cdens2[k], var = mod_surv$VCV[i,
                1] + mod_surv$VCV[i, 3], link.inv = plogis)
            modsurvsizepreg_prop_mass_ijk[i, j, k] = QGmean(mu = mod_surv$Sol[i,
                1] + mod_surv$Sol[i, 2] * cmasses[j] + mod_surv$Sol[i,
                3] + mod_surv$Sol[i, 11] * cmasses[j] + mod_surv$Sol[i,
                4] * cdens2[k] + mod_surv$Sol[i, 5] * cdens2[k]^2 +
                mod_surv$Sol[i, 12] * cmasses[j] * cdens2[k] + mod_surv$Sol[i,
                13] * cdens2[k] + mod_surv$Sol[i, 14] * cmasses[j] *
                cdens2[k], var = mod_surv$VCV[i, 1] + mod_surv$VCV[i,
                3], link.inv = plogis)
        }
    }
}
```

```
modlrs_mass_ijk = array(dim = c(n_samp, length(masses), n_dens2))
for (i in 1:n_samp) {
    for (k in 1:n_dens2) {
        for (j in 1:length(masses)) {
            modlrs_mass_ijk[i, j, k] = QGmean(mu = mod_slres_nopreg$Sol[i,
                1] + mod_slres_nopreg$Sol[i, 2] * cmasses[j] + mod_slres_nopreg$Sol[i,
                3] * cdens2[k] + mod_slres_nopreg$Sol[i, 4] * cdens2[k]^2 +
                mod_slres_nopreg$Sol[i, 10] * cdens2[k] * cmasses[j],
                var = mod_slres_nopreg$VCV[i, 1] + mod_slres_nopreg$VCV[i,
                  3], link.inv = exp)
        }
    }
}
```

```
modars_mass_ijk = array(dim = c(n:samp, length(masses), n_dens2))
for (i in 1:n_samp) {
    for (k in 1:n_dens2) {
        for (j in 1:length(masses)) {
            modars_mass_ijk[i, j, k] <- QGmean(mu = mod_fyres$Sol[i,
                1] + mod_fyres$Sol[i, 2] * cmasses[j] + mod_fyres$Sol[i,
                5] * cdens2[k] + mod_fyres$Sol[i, 6] * cdens2[k]^2,
                var = mod_fyres$VCV[i, 1], link.inv = plogis)
        }
    }
}
```

Code for figure SI.2.1.

```
pdf("ewes_FigSI2_1.pdf", height = 6, width = 6)
par(xpd = T, oma = c(4.5, 4, 0, 5), mar = c(0, 0.5, 1, 0.5), mfrow = c(2,
    2))
```

```
image(t(modsurvsizepreg_prop_mass_plot), axes = FALSE, col = grey_pal,
    frame.plot = FALSE, lwd = 2, useRaster = TRUE)
contour(t(modsurvsizepreg_prop_mass_plot), add = TRUE, fg = 9, col = teal,
    cex = 0.95, lwd = 2)
axis(side = 2, at = seq(0, 1, length.out = 6), labels = seq(3, 18,
    length.out = 6), las = 1)
text(x = 0.04, y = 1.03, labels = expression(bold("(a)")))
image(t(modsurvsizenopreg_prop_mass_plot), axes = FALSE, col = grey_pal,
    frame.plot = FALSE, useRaster = TRUE)
contour(t(modsurvsizenopreg_prop_mass_plot), add = TRUE, fg = 9, col = teal,
    cex = 0.95, lwd = 2)
text(x = 0.04, y = 1.03, labels = expression(bold("(b)")))
image(t(modars_mass_plot), axes = FALSE, col = grey_pal, frame.plot = FALSE,
    useRaster = TRUE)
contour(t(modars_mass_plot), add = TRUE, fg = 9, col = teal, cex = 0.95,
    lwd = 2)
axis(side = 2, at = seq(0, 1, length.out = 6), labels = seq(3, 18,
    length.out = 6), las = 1)  #rownames
axis(side = 1, at = seq(0, 1, length.out = 8), labels = FALSE)  #colnames
text(seq(0, 1, length.out = 8), rep(0, 8) - 0.05, labels = seq(321,
    671, length.out = 8), srt = 45, pos = 1, xpd = NA, cex = 1)
mtext("body mass (kg)", 2, line = 3, at = 1.1, cex = 0.9)
text(x = 0.04, y = 1.03, labels = expression(bold("(c)")))
image(t(modlrs_mass_plot), axes = FALSE, col = grey_pal, frame.plot = TRUE,
    useRaster = TRUE)
contour(t(modlrs_mass_plot), add = TRUE, fg = 9, col = teal, cex = 0.95,
    lwd = 2)
axis(side = 1, at = seq(0, 1, length.out = 8), labels = FALSE)  #colnames
text(seq(0, 1, length.out = 8), rep(0, 8) - 0.05, labels = seq(321,
    671, length.out = 8), srt = 45, pos = 1, xpd = NA, cex = 1)
mtext("population density", side = 1, line = 3, cex = 0.9, at = -0.1)
text(x = 0.04, y = 1.03, labels = expression(bold("(d)")))
dev.off()
```

6.5 Supporting Information 3 - bivariate association of early pregnancy and body mass

6.5.1 Model

```
prior = list(R = list(V = diag(2), nu = 3, fix = 2), G = list(G1 = list(V = diag(2),
    nu = 3, alpha.mu = c(0, 0), alpha.V = diag(2) * 1000), G2 = list(V = diag(2),
    nu = 3, alpha.mu = c(0, 0), alpha.V = diag(2) * 1000), G3 = list(V = diag(2),
    nu = 3, alpha.mu = c(0, 0), alpha.V = diag(2) * 1000)))
mod_bivariate = MCMCglmm(cbind(MumWeight, pregnancy) ~ trait - 1 +
    at.level(trait, 1):bith_date_c + at.level(trait, 1):measure_date_c +
    (twin + density_c + I(density_c^2) + mum_age_c + I(mum_age_c^2)) *
        trait, random = ~us(trait):animal + us(trait):mum_id + us(trait):cohort,
    rcov = ~us(trait):units, family = c("gaussian", "categorical"),
    data = data_ewes2, prior = prior, verbose = TRUE, nitt = 2e+06,
    thin = 50, burnin = 1500000, pedigree = data_ped)
mod_bivariate$Sol = mod_bivariate$Sol[8001:10000, ]
mod_bivariate$VCV = mod_bivariate$VCV[8001:10000, ]
```

6.5.2 Deriving quantities in the latent scale

Mean phenotype and variance-covariance matrix.

```
## mean values
mu_lat_all = mod_bivariate$Sol
mu_lat_mean = apply(mu_lat_all, 2, mean)
mu_lat_mode = posterior.mode(as.mcmc(mu_lat_all))
mu_lat_hpd = apply(mu_lat_all, 2, function(x) HPDinterval(as.mcmc(x)))
## vaiance estimates for table
var_lat_all = mod_bivariate$VCV
var_lat_mode = apply(var_lat_all, 2, function(x) posterior.mode(as.mcmc(x)))
var_lat_hpd = apply(var_lat_all, 2, function(x) HPDinterval(as.mcmc(x)))
### additive genetic and phenotypic variance-covariance matrices
G_all = list()
E1_all = list()
E2_all = list()
E3_all = list()
E_all = list()
P_all = list()
G_corr_all = list()
E_corr_all = list()
P_corr_all = list()
for (i in 1:n_samp) {
    G_all[[i]] = matrix(as.numeric(mod_bivariate$VCV[i, 1:4]), 2,
        2)
    E1_all[[i]] = matrix(as.numeric(mod_bivariate$VCV[i, 5:8]), 2,
        2)
    E2_all[[i]] = matrix(as.numeric(mod_bivariate$VCV[i, 9:12]), 2,
        2)
    E3_all[[i]] = matrix(as.numeric(mod_bivariate$VCV[i, 13:16]),
        2, 2)
    E_all[[i]] = E1_all[[i]] + E2_all[[i]] + E3_all[[i]]
    P_all[[i]] = G_all[[i]] + E_all[[i]]
    G_corr_all[[i]] = cov2cor(G_all[[i]])
    E_corr_all[[i]] = cov2cor(E_all[[i]])
    P_corr_all[[i]] = cov2cor(P_all[[i]])
}
G_all_array = do.call(cbind, G_all)
G_all_array = array(G_all_array, dim = c(dim(G_all[[1]]), length(G_all)))
G_mean = apply(G_all_array, c(1, 2), mean, na.rm = TRUE)
G_mode = apply(G_all_array, c(1, 2), function(x) posterior.mode(as.mcmc(x)))
G_95CrIU = G_95CrIL = matrix(ncol = 2, nrow = 2)
G_95CrIU[1, 1] = HPDinterval(as.mcmc(G_all_array[1, 1, ]), prob = 0.95)[2]
G_95CrIL[1, 1] = HPDinterval(as.mcmc(G_all_array[1, 1, ]), prob = 0.95)[1]
G_95CrIU[1, 2] = G_95CrIU[2, 1] = HPDinterval(as.mcmc(G_all_array[1,
    2, ]), prob = 0.95)[2]
G_95CrIL[1, 2] = G_95CrIL[2, 1] = HPDinterval(as.mcmc(G_all_array[1,
    2, ]), prob = 0.95)[1]
G_95CrIU[2, 2] = HPDinterval(as.mcmc(G_all_array[2, 2, ]), prob = 0.95)[2]
G_95CrIL[2, 2] = HPDinterval(as.mcmc(G_all_array[2, 2, ]), prob = 0.95)[1]
G_corr_all_array = do.call(cbind, G_corr_all)
G_corr_all_array = array(G_corr_all_array, dim = c(dim(G_corr_all[[1]]),
    length(G_corr_all)))
```

```
G_corr_mean = apply(G_corr_all_array, c(1, 2), mean, na.rm = TRUE)
G_corr_mode = apply(G_corr_all_array, c(1, 2), function(x) posterior.mode(as.mcmc(x)))
G_corr_95CrIU = G_corr_95CrIL = diag(2)
G_corr_95CrIU[1, 2] = G_corr_95CrIU[2, 1] = HPDinterval(as.mcmc(G_corr_all_array[1,
    2, ]), prob = 0.95)[2]
G_corr_95CrIL[1, 2] = G_corr_95CrIL[2, 1] = HPDinterval(as.mcmc(G_corr_all_array[1,
    2, ]), prob = 0.95)[1]
E_all_array = do.call(cbind, E_all)
E_all_array = array(E_all_array, dim = c(dim(E_all[[1]]), length(E_all)))
E_mean = apply(E_all_array, c(1, 2), mean, na.rm = TRUE)
E_mode = apply(E_all_array, c(1, 2), function(x) posterior.mode(as.mcmc(x)))
E_95CrIU = E_95CrIL = matrix(ncol = 2, nrow = 2)
E_95CrIU[1, 1] = HPDinterval(as.mcmc(E_all_array[1, 1, ]), prob = 0.95)[2]
E_95CrIL[1, 1] = HPDinterval(as.mcmc(E_all_array[1, 1, ]), prob = 0.95)[1]
E_95CrIU[1, 2] = E_95CrIU[2, 1] = HPDinterval(as.mcmc(E_all_array[1,
    2, ]), prob = 0.95)[2]
E_95CrIL[1, 2] = E_95CrIL[2, 1] = HPDinterval(as.mcmc(E_all_array[1,
    2, ]), prob = 0.95)[1]
E_95CrIU[2, 2] = HPDinterval(as.mcmc(E_all_array[2, 2, ]), prob = 0.95)[2]
E_95CrIL[2, 2] = HPDinterval(as.mcmc(E_all_array[2, 2, ]), prob = 0.95)[1]
E_corr_all_array = do.call(cbind, E_corr_all)
E_corr_all_array = array(E_corr_all_array, dim = c(dim(E_corr_all[[1]]),
    length(E_corr_all)))
E_corr_mean = apply(E_corr_all_array, c(1, 2), mean, na.rm = TRUE)
E_corr_mode = apply(E_corr_all_array, c(1, 2), function(x) posterior.mode(as.mcmc(x)))
E_corr_95CrIU = E_corr_95CrIL = diag(2)
E_corr_95CrIU[1, 2] = E_corr_95CrIU[2, 1] = HPDinterval(as.mcmc(E_corr_all_array[1,
    2, ]))[2]
E_corr_95CrIL[1, 2] = E_corr_95CrIL[2, 1] = HPDinterval(as.mcmc(E_corr_all_array[1,
    2, ]))[1]
P_all_array = do.call(cbind, P_all)
P_all_array = array(P_all_array, dim = c(dim(P_all[[1]]), length(P_all)))
P_mean = apply(P_all_array, c(1, 2), mean, na.rm = TRUE)
P_mode = apply(P_all_array, c(1, 2), function(x) posterior.mode(as.mcmc(x)))
P_95CrIU = P_95CrIL = matrix(ncol = 2, nrow = 2)
P_95CrIU[1, 1] = HPDinterval(as.mcmc(P_all_array[1, 1, ]), prob = 0.95)[2]
P_95CrIL[1, 1] = HPDinterval(as.mcmc(P_all_array[1, 1, ]), prob = 0.95)[1]
P_95CrIU[1, 2] = P_95CrIU[2, 1] <- HPDinterval(as.mcmc(P_all_array[1,
    2, ]), prob = 0.95)[2]
P_95CrIL[1, 2] = P_95CrIL[2, 1] <- HPDinterval(as.mcmc(P_all_array[1,
    2, ]), prob = 0.95)[1]
P_95CrIU[2, 2] = HPDinterval(as.mcmc(P_all_array[2, 2, ]), prob = 0.95)[2]
P_95CrIL[2, 2] = HPDinterval(as.mcmc(P_all_array[2, 2, ]), prob = 0.95)[1]
P_corr_all_array = do.call(cbind, P_corr_all)
P_corr_all_array = array(P_corr_all_array, dim = c(dim(P_corr_all[[1]]),
    length(P_corr_all)))
P_corr_mean = apply(P_corr_all_array, c(1, 2), mean, na.rm = TRUE)
P_corr_mode = apply(P_corr_all_array, c(1, 2), function(x) posterior.mode(as.mcmc(x)))
P_corr_95CrIU = P_corr_95CrIL = diag(2)
P_corr_95CrIU[1, 2] = P_corr_95CrIU[2, 1] = HPDinterval(as.mcmc(P_corr_all_array[1,
    2, ]))[2]
P_corr_95CrIL[1, 2] = P_corr_95CrIL[2, 1] = HPDinterval(as.mcmc(P_corr_all_array[1,
    2, ]))[1]
```

Heritability.

```
h2_mass_lat_mode = posterior.mode(as.mcmc(G_all_array[1, 1, ]/P_all_array[1,
    1, ]))
h2_mass_lat_hpd = HPDinterval(as.mcmc(G_all_array[1, 1, ]/P_all_array[1,
    1, ]))
```

6.5.3 Deriving quantities in the scale in which traits are expressed

Mean phenotype.

```
mu_dat_all = array(dim = c(n_samp, 2))
for (i in 1:n_samp) {
    f_muDat = function(lat) {
        c(lat[1], plogis(lat[2])) * dmvnorm(lat, mu_lat_all[i, 1:2],
            P_all_array[1:2, 1:2, i])
    }
    mu_dat_all[i, ] = cuhre(nComp = 2, f = f_muDat, lower = mu_lat_all[i,
        1:2] - 5 * sqrt(diag(P_all_array[1:2, 1:2, i])), upper = mu_lat_all[i,
        1:2] + 5 * sqrt(diag(P_all_array[1:2, 1:2, i])), flags = list(verbose = 0))$integral
}
```

Variance-covariance matrix.

```
sigma_dat_all = array(dim = c(n_samp, 2, 2))
for (i in 1:n_samp) {
    f_sigma_dat <- function(lat) {
        as.vector((c(lat[1], plogis(lat[2])) %*% t(c(lat[1], plogis(lat[2])))) *
            dmvnorm(lat, mu_lat_all[i, 1:2], P_all_array[1:2, 1:2,
                i]))
    }
    second_non_central = cuhre(nComp = 4, f = f_sigma_dat, lower = mu_lat_all[i,
        1:2] - 5 * sqrt(diag(P_all_array[1:2, 1:2, i])), upper = mu_lat_all[i,
        1:2] + 5 * sqrt(diag(P_all_array[1:2, 1:2, i])), flags = list(verbose = 0))$integral
    sigma_dat_all[i, 1:2, 1:2] = matrix(second_non_central, 2, 2) -
        mu_dat_all[i, 1:2] %*% t(mu_dat_all[i, 1:2])
}
```

```
P_dat_all_array = sigma_dat_all
P_dat_mean = apply(P_dat_all_array, c(2, 3), mean, na.rm = TRUE)
P_dat_mode = apply(P_dat_all_array, c(2, 3), function(x) posterior.mode(as.mcmc(x)))
P_dat_95CrIU = P_dat_95CrIL = matrix(ncol = 2, nrow = 2)
P_dat_95CrIU[1, 1] = HPDinterval(as.mcmc(P_dat_all_array[, 1, 1]),
    prob = 0.95)[2]
P_dat_95CrIL[1, 1] = HPDinterval(as.mcmc(P_dat_all_array[, 1, 1]),
    prob = 0.95)[1]
P_dat_95CrIU[1, 2] = P_dat_95CrIU[2, 1] <- HPDinterval(as.mcmc(P_dat_all_array[,
    1, 2]), prob = 0.95)[2]
P_dat_95CrIL[1, 2] = P_dat_95CrIL[2, 1] <- HPDinterval(as.mcmc(P_dat_all_array[,
    1, 2]), prob = 0.95)[1]
P_dat_95CrIU[2, 2] = HPDinterval(as.mcmc(P_dat_all_array[, 2, 2]),
    prob = 0.95)[2]
P_dat_95CrIL[2, 2] = HPDinterval(as.mcmc(P_dat_all_array[, 2, 2]),
```

```
    prob = 0.95)[1]
P_dat_corr_all_array = sigma_dat_all
for (i in 1:dim(sigma_dat_all)[1]) {
    P_dat_corr_all_array[i, 1:2, 1:2] <- cov2cor(sigma_dat_all[i,
        1:2, 1:2])
}
P_dat_corr_mean = apply(P_dat_corr_all_array, c(2, 3), mean, na.rm = TRUE)
P_dat_corr_mode = apply(P_dat_corr_all_array, c(2, 3), function(x) posterior.mode(as.mcmc(x)))
P_dat_corr_95CrIU = P_dat_corr_95CrIL = diag(2)
P_dat_corr_95CrIU[1, 2] = P_dat_corr_95CrIU[2, 1] = HPDinterval(as.mcmc(P_dat_corr_all_array[,
    1, 2]))[2]
P_dat_corr_95CrIL[1, 2] = P_dat_corr_95CrIL[2, 1] = HPDinterval(as.mcmc(P_dat_corr_all_array[,
    1, 2]))[1]
```

Additive genetic variance-covariance matrix.

```
QGpsi_all = array(dim = c(n_samp, 2))
G_dat_all = array(dim = c(n_samp, 2, 2))
G_dat_corr_all = array(dim = c(n_samp, 2, 2))
for (i in 1:n_samp) {
    f_QGpsi = function(lat) {
        c(1, dlogis(lat[2])) * dmvnorm(lat, mu_lat_all[i, 1:2], P_all_array[1:2,
            1:2, i])
    }
    QGpsi_all[i, ] = cuhre(nComp = 2, f = f_QGpsi, lower = mu_lat_all[i,
        1:2] - 5 * sqrt(diag(P_all_array[1:2, 1:2, i])), upper = mu_lat_all[i,
        1:2] + 5 * sqrt(diag(P_all_array[1:2, 1:2, i])), flags = list(verbose = 0))$integral
    G_dat_all[i, 1:2, 1:2] = diag(QGpsi_all[i, ]) %*% G_all_array[1:2,
        1:2, i] %*% t(diag(QGpsi_all[i, ]))
    G_dat_corr_all[i, 1:2, 1:2] <- cov2cor(G_dat_all[i, 1:2, 1:2])
}
```

```
G_dat_mean = apply(G_dat_all, c(2, 3), mean, na.rm = TRUE)
G_dat_mode = apply(G_dat_all, c(2, 3), function(x) posterior.mode(as.mcmc(x)))
G_dat_95CrIU = G_dat_95CrIL = matrix(ncol = 2, nrow = 2)
G_dat_95CrIU[1, 1] = HPDinterval(as.mcmc(G_dat_all[, 1, 1]), prob = 0.95)[2]
G_dat_95CrIL[1, 1] = HPDinterval(as.mcmc(G_dat_all[, 1, 1]), prob = 0.95)[1]
G_dat_95CrIU[1, 2] = G_dat_95CrIU[2, 1] = HPDinterval(as.mcmc(G_dat_all[,
    1, 2]), prob = 0.95)[2]
G_dat_95CrIL[1, 2] = G_dat_95CrIL[2, 1] = HPDinterval(as.mcmc(G_dat_all[,
    1, 2]), prob = 0.95)[1]
G_dat_95CrIU[2, 2] = HPDinterval(as.mcmc(G_dat_all[, 2, 2]), prob = 0.95)[2]
G_dat_95CrIL[2, 2] = HPDinterval(as.mcmc(G_dat_all[, 2, 2]), prob = 0.95)[1]
G_dat_corr_mean = apply(G_dat_corr_all, c(2, 3), mean, na.rm = TRUE)
G_dat_corr_mode = apply(G_dat_corr_all, c(2, 3), function(x) posterior.mode(as.mcmc(x)))
G_dat_corr_95CrIU = G_dat_corr_95CrIL = matrix(ncol = 2, nrow = 2)
G_dat_corr_95CrIU[1, 1] = HPDinterval(as.mcmc(G_dat_corr_all[, 1,
    1]), prob = 0.95)[2]
G_dat_corr_95CrIL[1, 1] = HPDinterval(as.mcmc(G_dat_corr_all[, 1,
    1]), prob = 0.95)[1]
G_dat_corr_95CrIU[1, 2] = G_dat_corr_95CrIU[2, 1] = HPDinterval(as.mcmc(G_dat_corr_all[,
    1, 2]), prob = 0.95)[2]
G_dat_corr_95CrIL[1, 2] = G_dat_corr_95CrIL[2, 1] = HPDinterval(as.mcmc(G_dat_corr_all[,
```

```
    1, 2]), prob = 0.95)[1]
G_dat_corr_95CrIU[2, 2] = HPDinterval(as.mcmc(G_dat_corr_all[, 2,
    2]), prob = 0.95)[2]
G_dat_corr_95CrIL[2, 2] = HPDinterval(as.mcmc(G_dat_corr_all[, 2,
    2]), prob = 0.95)[1]
```

Heritabilities.

```
h2_mass_dat_mode = posterior.mode(as.mcmc(G_dat_all[, 1, 1]/P_dat_all_array[,
    1, 1]))
h2_mass_dat_hpd = HPDinterval(as.mcmc(G_dat_all[, 1, 1]/P_dat_all_array[,
    1, 1], prob = 0.95))
h2_p_dat_mode = posterior.mode(as.mcmc(G_dat_all[, 2, 2]/P_dat_all_array[,
    2, 2]))
h2_p_dat_hpd = HPDinterval(as.mcmc(G_dat_all[, 2, 2]/P_dat_all_array[,
    2, 2], prob = 0.95))
```

Conditional genetic variances.

```
va_mass_cond_all = array(dim = n_samp)
va_preg_cond_all = array(dim = n_samp)
for (i in 1:n_samp) {
    va_mass_cond_all[i] = G_dat_all[i, 1, 1] - G_dat_all[i, 1, 2]^2/G_dat_all[i,
        2, 2]
    va_preg_cond_all[i] = G_dat_all[i, 2, 2] - G_dat_all[i, 1, 2]^2/G_dat_all[i,
        1, 1]
}
va_mass_cond = posterior.mode(as.mcmc(va_mass_cond_all))
va_mass_cond_hpd = HPDinterval(as.mcmc(va_mass_cond_all))
va_preg_cond = posterior.mode(as.mcmc(va_preg_cond_all))
va_preg_cond_hpd = HPDinterval(as.mcmc(va_preg_cond_all))
prop_va_mass_cond_all = va_mass_cond_all/G_dat_all[, 1, 1]
prop_va_mass_cond = posterior.mode(as.mcmc(prop_va_mass_cond_all))
prop_va_mass_cond_hpd = HPDinterval(as.mcmc(prop_va_mass_cond_all))
prop_va_preg_cond_all = va_preg_cond_all/G_dat_all[, 2, 2]  #same as above
prop_va_preg_cond = posterior.mode(as.mcmc(prop_va_preg_cond_all))
prop_va_preg_cond_hpd = HPDinterval(as.mcmc(prop_va_preg_cond_all))
```

6.6 Supporting Information 4 - selection of the probabilitistic reaction norm for early pregnancy

6.6.1 Random regression animal model

Code for fitting the model in Equation SI4.1 (Supporting Information 4).

```
prior = list(G = list(G1 = list(V = diag(2) * (0.002/1.002), nu = 1.002),
    G2 = list(V = 1, nu = 1, alpha.mu = 0, alpha.V = 1000)), R = list(V = 1,
    nu = 1, fix = 1))
MCMCfactor = 50
mod_preg_gen = MCMCglmm(pregnancy ~ mass_c + density_c + I(density_c^2),
    random = ~us(1 + mass_c):animal + cohort, rcov = ~units, family = "categorical",
    data = data_ewes2, prior = prior, pedigree = data_ped, nitt = 25000 *
        MCMCfactor, thin = 10 * MCMCfactor, burnin = 10000 * MCMCfactor)
```

```
mod_preg_gen_sol = posterior.mode(mod_preg_gen$Sol)
mod_preg_gen_sol_hpd = HPDinterval(mod_preg_gen$Sol)
mod_preg_gen_vcv = posterior.mode(mod_preg_gen$VCV)
mod_preg_gen_vcv_hpd = HPDinterval(mod_preg_gen$VCV)
```

6.6.2 Conditional individual fitness functions of (mean) body mass

Function to calculate conditional individual fitness functions of (mean) body mass for non-pregnant Soay sheep females.

```
f_z_bf_i_p0 = function(eps, density_i, mass) {
    eps_twin = eps[, 1, ]
    eps_mass = eps[, 2, ]
    eps_preg = eps[, 3, ]
    eps_surv = eps[, 4, ]
    eps_fyres = eps[, 5, ]
    eps_slres = eps[, 6, ]
    twin_i <- plogis(mod_twin$Sol[, 1] + mod_twin$Sol[, 2] * rep(density_i,
        n_samp) + mod_twin$Sol[, 3] * rep(density_i, n_samp)^2 + eps_twin)
    mass_i = rep(mass, n_samp) + eps_mass
    pregnancy_i = rep(0, n_samp) + eps_preg
    survival_i <- plogis(mod_surv$Sol[, 1] + mod_surv$Sol[, 2] * mass_i +
        mod_surv$Sol[, 3] * pregnancy_i + mod_surv$Sol[, 11] * pregnancy_i *
        mass_i + mod_surv$Sol[, 8] * twin_i + mod_surv$Sol[, 4] *
        rep(density_i, n_samp) + mod_surv$Sol[, 5] * rep(density_i,
        n_samp)^2 + mod_surv$Sol[, 12] * mass_i * rep(density_i, n_samp) +
        mod_surv$Sol[, 13] * pregnancy_i * rep(density_i, n_samp) +
        mod_surv$Sol[, 14] * mass_i * pregnancy_i * rep(density_i,
            n_samp) + eps_surv)
    fyres_i = plogis(mod_fyres$Sol[, 1] + mod_fyres$Sol[, 2] * mass_i +
        eps_fyres)
    slres_i = exp(mod_slres_nopreg$Sol[, 1] + mod_slres_nopreg$Sol[,
        2] * mass_i + mod_slres_nopreg$Sol[, 5] * twin_i + mod_slres_nopreg$Sol[,
        3] * density_i + mod_slres_nopreg$Sol[, 4] * density_i^2 +
        mod_slres_nopreg$Sol[, 10] * mass_i * rep(density_i, n_samp) +
        eps_slres)
    w_i = survival_i * ((pregnancy_i * fyres_i) + slres_i)
    (array(c(twin_i, mass_i, pregnancy_i, survival_i, fyres_i, slres_i,
        w_i), dim = c(n_MC, n_samp, 7)))
}
```

Function to calculate conditional individual fitness functions of (mean) body mass for pregnant Soay sheep females.

```
f_z_bf_i_p1 = function(eps, density_i, mass) {
    eps_twin = eps[, 1, ]
    eps_mass = eps[, 2, ]
    eps_preg = eps[, 3, ]
    eps_surv = eps[, 4, ]
    eps_fyres = eps[, 5, ]
    eps_slres = eps[, 6, ]
    twin_i = plogis(mod_twin$Sol[, 1] + mod_twin$Sol[, 2] * rep(density_i,
        n_samp) + mod_twin$Sol[, 3] * rep(density_i, n_samp)^2 + eps_twin)
```

```
    mass_i = rep(mass, n_samp) + eps_mass
    pregnancy_i = rep(1, n_samp) + eps_preg
    survival_i = plogis(mod_surv$Sol[, 1] + mod_surv$Sol[, 2] * mass_i +
        mod_surv$Sol[, 3] * pregnancy_i + mod_surv$Sol[, 11] * pregnancy_i *
        mass_i + mod_surv$Sol[, 8] * twin_i + mod_surv$Sol[, 4] *
        rep(density_i, n_samp) + mod_surv$Sol[, 5] * rep(density_i,
        n_samp)^2 + mod_surv$Sol[, 12] * mass_i * rep(density_i, n_samp) +
        mod_surv$Sol[, 13] * pregnancy_i * rep(density_i, n_samp) +
        mod_surv$Sol[, 14] * mass_i * pregnancy_i * rep(density_i,
            n_samp) + eps_surv)
    fyres_i = plogis(mod_fyres$Sol[, 1] + mod_fyres$Sol[, 2] * mass_i +
        eps_fyres)
    slres_i = exp(mod_slres_nopreg$Sol[, 1] + mod_slres_nopreg$Sol[,
        2] * mass_i + mod_slres_nopreg$Sol[, 5] * twin_i + mod_slres_nopreg$Sol[,
        3] * density_i + mod_slres_nopreg$Sol[, 4] * density_i^2 +
        mod_slres_nopreg$Sol[, 10] * mass_i * rep(density_i, n_samp) +
        eps_slres)
    w_i = survival_i * ((pregnancy_i * fyres_i) + slres_i)
    (array(c(twin_i, mass_i, pregnancy_i, survival_i, fyres_i, slres_i,
        w_i), dim = c(n_MC, n_samp, 7)))
}
```

Applying the functions above to obtain the conditional individual fitness functions.

```
n_mass = 40
n_dens = 9
masses = seq(-6, 6, length.out = n_mass)
densities = seq(321, 671, length.out = n_dens) - mean(data_ewes2$density,
    na.rm = TRUE)
hp = 0.2
eps = array(dim = c(n_MC, 6, n_samp))
for (i in 1:n_samp) eps[, , i] = rmvnorm(n_MC, rep(0, 6), sigma_eps_all[[i]])
grid = expand.grid(densities, masses)
zbar_p0_dens_all = array(dim = c(n_samp, 7, n_dens, n_mass))
zbar_p1_dens_all = array(dim = c(n_samp, 7, n_dens, n_mass))
# indexes to avoid nested loops
ks = rep(1:n_dens, dim(grid)[1]/n_dens)
js = rep(1:n_mass, times = dim(grid)[1]/n_mass/n_dens, each = n_dens)
for (i in 1:dim(grid)[1]) {
    zbar_p0_k_nMC = f_z_bf_i_p0(eps, density_i = grid[i, 1], mass = grid[i,
        2])
    zbar_p0_k = apply(zbar_p0_k_nMC, c(2, 3), mean)
    zbar_p1_k_nMC = f_z_bf_i_p1(eps, density_i = grid[i, 1], mass = grid[i,
        2])
    zbar_p1_k = apply(zbar_p1_k_nMC, c(2, 3), mean)
    k = ks[i]
    j = js[i]
    zbar_p0_dens_all[, , k, j] = zbar_p0_k
    zbar_p1_dens_all[, , k, j] = zbar_p1_k
}
```

```
zbar_p0_dens = apply(zbar_p0_dens_all, c(2:4), mean)
zbar_p1_dens = apply(zbar_p1_dens_all, c(2:4), mean)
```

6.6.3 Extended selection gradients for the intercept and slope of the logistic regression of the probability of pregnancy on mass

Function for intercepts.

```
f_z_bf_i_int = function(eps, x, density_i, mass) {
    eps_twin = eps[, 1, ]
    eps_mass = eps[, 2, ]
    eps_preg = eps[, 3, ]
    eps_surv = eps[, 4, ]
    eps_fyres = eps[, 5, ]
    eps_slres = eps[, 6, ]
    twin_i = plogis(mod_twin$Sol[, 1] + mod_twin$Sol[, 2] * rep(density_i,
        n_samp) + mod_twin$Sol[, 3] * rep(density_i, n_samp)^2 + eps_twin)
    mass_i = rep(mass, n_samp) + eps_mass
    pregnancy_i = plogis(rep(preg_int, n_samp) + rep(preg_slope, n_samp) *
        mass_i + mod_preg$Sol[, 9] * twin_i + mod_preg$Sol[, 5] *
        rep(density_i, n_samp) + mod_preg$Sol[, 6] * rep(density_i,
        n_samp)^2 + mod_preg$Sol[, 12] * mass_i * rep(density_i, n_samp) +
        eps_preg)
    pregnancy_i = plogis(mod_preg$Sol[, 1] + x + mod_preg$Sol[, 2] *
        mass_i + mod_preg$Sol[, 3] * mass_i^2 + mod_preg$Sol[, 4] *
        mass_i^3 + mod_preg$Sol[, 9] * twin_i + mod_preg$Sol[, 5] *
        density_i + mod_preg$Sol[, 6] * density_i^2 + mod_preg$Sol[,
        12] * mass_i * density_i + eps_preg)
    survival_i = plogis(mod_surv$Sol[, 1] + mod_surv$Sol[, 2] * mass_i +
        mod_surv$Sol[, 3] * pregnancy_i + mod_surv$Sol[, 11] * pregnancy_i *
        mass_i + mod_surv$Sol[, 8] * twin_i + mod_surv$Sol[, 4] *
        rep(density_i, n_samp) + mod_surv$Sol[, 5] * rep(density_i,
        n_samp)^2 + mod_surv$Sol[, 12] * mass_i * rep(density_i, n_samp) +
        mod_surv$Sol[, 13] * pregnancy_i * rep(density_i, n_samp) +
        mod_surv$Sol[, 14] * mass_i * pregnancy_i * rep(density_i,
            n_samp) + eps_surv)
    fyres_i = plogis(mod_fyres$Sol[, 1] + mod_fyres$Sol[, 2] * mass_i +
        eps_fyres)
    slres_i = exp(mod_slres_nopreg$Sol[, 1] + mod_slres_nopreg$Sol[,
        2] * mass_i + mod_slres_nopreg$Sol[, 5] * twin_i + mod_slres_nopreg$Sol[,
        3] * density_i + mod_slres_nopreg$Sol[, 4] * density_i^2 +
        mod_slres_nopreg$Sol[, 10] * mass_i * rep(density_i, n_samp) +
        eps_slres)
    w_i = survival_i * ((pregnancy_i * fyres_i) + slres_i)
    return(array(c(twin_i, mass_i, pregnancy_i, survival_i, fyres_i,
        slres_i, w_i), dim = c(n_MC, n_samp, 7)))
}
```

Function for slopes.

```
f_z_bf_i_sl <- function(eps, x, density_i, mass) {
    eps_twin = eps[, 1, ]
    eps_mass = eps[, 2, ]
```

```
    eps_preg = eps[, 3, ]
    eps_surv = eps[, 4, ]
    eps_fyres = eps[, 5, ]
    eps_slres = eps[, 6, ]
    twin_i = plogis(mod_twin$Sol[, 1] + mod_twin$Sol[, 2] * rep(density_i,
        n_samp) + mod_twin$Sol[, 3] * rep(density_i, n_samp)^2 + eps_twin)
    mass_i = rep(mass, n_samp) + eps_mass
    pregnancy_i = plogis(mod_preg$Sol[, 1] + (mod_preg$Sol[, 2] +
        x) * mass_i + mod_preg$Sol[, 3] * mass_i^2 + mod_preg$Sol[,
        4] * mass_i^3 + mod_preg$Sol[, 9] * twin_i + mod_preg$Sol[,
        5] * density_i + mod_preg$Sol[, 6] * density_i^2 + mod_preg$Sol[,
        12] * mass_i * density_i + eps_preg)
    survival_i = plogis(mod_surv$Sol[, 1] + mod_surv$Sol[, 2] * mass_i +
        mod_surv$Sol[, 3] * pregnancy_i + mod_surv$Sol[, 11] * pregnancy_i *
        mass_i + mod_surv$Sol[, 8] * twin_i + mod_surv$Sol[, 4] *
        rep(density_i, n_samp) + mod_surv$Sol[, 5] * rep(density_i,
        n_samp)^2 + mod_surv$Sol[, 12] * mass_i * rep(density_i, n_samp) +
        mod_surv$Sol[, 13] * pregnancy_i * rep(density_i, n_samp) +
        mod_surv$Sol[, 14] * mass_i * pregnancy_i * rep(density_i,
            n_samp) + eps_surv)
    fyres_i = plogis(mod_fyres$Sol[, 1] + mod_fyres$Sol[, 2] * mass_i +
        eps_fyres)
    slres_i = exp(mod_slres_nopreg$Sol[, 1] + mod_slres_nopreg$Sol[,
        2] * mass_i + mod_slres_nopreg$Sol[, 5] * twin_i + mod_slres_nopreg$Sol[,
        3] * density_i + mod_slres_nopreg$Sol[, 4] * density_i^2 +
        mod_slres_nopreg$Sol[, 10] * mass_i * rep(density_i, n_samp) +
        eps_slres)
    w_i = survival_i * ((pregnancy_i * fyres_i) + slres_i)
    (array(c(twin_i, mass_i, pregnancy_i, survival_i, fyres_i, slres_i,
        w_i), dim = c(n_MC, n_samp, 7)))
}
```

Using the functions above to calculate the extended selection gradients.

```
n_masses = 50
n_dens = 60
masses = seq(-2, 2, length.out = n_masses)
densities = seq(321, 671, length.out = n_dens) - mean(data_ewes2$density,
    na.rm = TRUE)
hp = 0.2
eps = array(dim = c(n_MC, 6, n_samp))
for (i in 1:n_samp) eps[, , i] = rmvnorm(n_MC, rep(0, 6), sigma_eps_all[[i]])
grid = expand.grid(densities, masses)
zbar_int_dens_all = array(dim = c(n_samp, 7, n_dens, n_masses))
zbar_sl_dens_all = array(dim = c(n_samp, 7, n_dens, n_masses))
eta_pregInt_dens_all = array(dim = c(n_samp, n_dens, n_masses))
eta_pregSl_dens_all = array(dim = c(n_samp, n_dens, n_masses))
# indexes to avoid nested loops
ks = rep(1:n_dens, dim(grid)[1]/n_dens)
js = rep(1:n_masses, times = dim(grid)[1]/n_masses/n_dens, each = n_dens)
for (i in 1:dim(grid)[1]) {
    zbar_int_k_nMC = f_z_bf_i_int(eps, x = 0, density_i = grid[i,
        1], mass = grid[i, 2])
    zbar_int_k = apply(zbar_int_k_nMC, c(2, 3), mean)
```

```
    zbar_sl_k_nMC = f_z_bf_i_sl(eps, x = 0, density_i = grid[i, 1],
        mass = grid[i, 2])
    zbar_sl_k = apply(zbar_sl_k_nMC, c(2, 3), mean)
    zbar_k_primeInt_nMC = f_z_bf_i_int(eps, x = hp, density_i = grid[i,
        1], mass = grid[i, 2])
    zbar_k_primeInt = apply(zbar_k_primeInt_nMC, c(2, 3), mean)
    zbar_k_primeSl_nMC = f_z_bf_i_sl(eps, x = hp, density_i = grid[i,
        1], mass = grid[i, 2])
    zbar_k_primeSl = apply(zbar_k_primeSl_nMC, c(2, 3), mean)
    k = ks[i]
    j = js[i]
    zbar_int_dens_all[, , k, j] = zbar_int_k
    zbar_sl_dens_all[, , k, j] = zbar_sl_k
    eta_pregInt_dens_all[, k, j] = (zbar_k_primeInt[, 7] - zbar_int_k[,
        7])/hp/zbar_int_k[, 7]
    eta_pregSl_dens_all[, k, j] = (zbar_k_primeSl[, 7] - zbar_sl_k[,
        7])/hp/zbar_sl_k[, 7]
}
```

```
eta_pregInt_dens <- apply(eta_pregInt_dens_all, c(2, 3), mean)
eta_pregSl_dens <- apply(eta_pregSl_dens_all, c(2, 3), mean)
```

6.6.4 Figure SI.4.1

```
pdf("ewes_FigSI4_1.pdf", height = 10, width = 7)
par(oma = c(6, 5, 4, 4), mar = c(0.5, 0.5, 0.5, 0.5))
layout(mat = matrix(c(12, 12, 12, 12, 12, 12, 3, 3, 4, 4, 5, 5, 6,
    6, 7, 7, 8, 8, 9, 9, 10, 10, 11, 11, 13, 13, 13, 13, 13, 13, 1,
    1, 1, 2, 2, 2), byrow = TRUE, ncol = 6), heights = c(0.4, 0.9,
    0.9, 0.9, 0.3, 1.5))
d = seq(321, 671, length.out = 8)
m = seq(-2, 2, length.out = 5) + mean(data_ewes2$MumWeight, na.rm = TRUE)
image(t(eta_pregInt_dens), axes = FALSE, col = grey_pal, frame.plot = FALSE,
    lwd = 2, useRaster = TRUE)
contour(t(eta_pregInt_dens), add = TRUE, fg = 9, col = teal, cex = 0.95,
    lwd = 2)
axis(side = 2, at = seq(0, 1, length.out = 5), labels = round(m, 0),
    las = 1, cex.axis = 1.2)
axis(side = 1, at = seq(0, 1, length.out = 8), labels = FALSE, las = 1,
    cex.axis = 1.2)
text(seq(0, 1, length.out = 8), rep(0, 8) - 0.05, labels = round(d,
    0), srt = 45, pos = 1, xpd = NA, cex = 1.2)
text(x = 0.06, y = 0.96, labels = expression(bold("(b1)")), cex = 1.2)
mtext(side = 1, "population density", line = 4, at = 1.05)
mtext(side = 2, "mean population \n body mass (kg)", line = 3)
image(t(eta_pregSl_dens), axes = FALSE, col = grey_pal, frame.plot = TRUE,
    lwd = 2, useRaster = TRUE)
axis(side = 1, at = seq(0, 1, length.out = 8), labels = FALSE)
text(seq(0, 1, length.out = 8), par("usr")[3] - 0.05, labels = round(d,
    0), srt = 45, pos = 1, xpd = NA, cex = 1.2)
contour(t(eta_pregSl_dens), add = TRUE, fg = 9, col = teal, cex = 0.95,
```

```
    lwd = 2)
text(x = 0.06, y = 0.96, labels = expression(bold("(b2)")), cex = 1.2)
n_mass = 40
n_dens = 9
masses = seq(-6, 6, length.out = n_mass) + mean(data_ewes2$MumWeight,
    na.rm = TRUE)
d = seq(321, 671, length.out = n_dens)
plot(masses, zbar_p0_dens[7, 1, ], type = "l", ylim = c(0, 6), las = 1,
    xlab = "", ylab = "", xaxt = "n", cex.axis = 1.2)
lines(masses, zbar_p1_dens[7, 1, ], col = teal)
text(7.75, 5.9, paste("N = ", d[1], sep = ""), cex = 1.2)
mtext(side = 3, line = 0.5, expression(bold("(a)")), at = 6.5)
plot(masses, zbar_p0_dens[7, 2, ], type = "l", ylim = c(0, 6), las = 1,
    xlab = "", ylab = "", xaxt = "n", yaxt = "n", cex.axis = 1.2)
lines(masses, zbar_p1_dens[7, 2, ], col = teal)
text(7.75, 5.9, paste("N = ", round(d[2], 0), sep = ""), cex = 1.2)
plot(masses, zbar_p0_dens[7, 3, ], type = "l", ylim = c(0, 6), las = 1,
    xlab = "", ylab = "", xaxt = "n", yaxt = "n", cex.axis = 1.2)
lines(masses, zbar_p1_dens[7, 3, ], col = teal)
text(7.75, 5.9, paste("N = ", round(d[3], 0), sep = ""), cex = 1.2)
plot(masses, zbar_p0_dens[7, 4, ], type = "l", ylim = c(0, 6), las = 1,
    xlab = "", ylab = "", xaxt = "n", cex.axis = 1.2)
lines(masses, zbar_p1_dens[7, 4, ], col = teal)
text(7.75, 5.9, paste("N = ", round(d[4], 0), sep = ""), cex = 1.2)
mtext(side = 2, "W", line = 3)
plot(masses, zbar_p0_dens[7, 5, ], type = "l", ylim = c(0, 6), las = 1,
    xlab = "", ylab = "", xaxt = "n", yaxt = "n", cex.axis = 1.2)
lines(masses, zbar_p1_dens[7, 5, ], col = teal)
text(7.75, 5.9, paste("N = ", round(d[5], 0), sep = ""), cex = 1.2)
plot(masses, zbar_p0_dens[7, 6, ], type = "l", ylim = c(0, 6), las = 1,
    xlab = "", ylab = "", xaxt = "n", yaxt = "n", cex.axis = 1.2)
lines(masses, zbar_p1_dens[7, 6, ], col = teal)
text(7.75, 5.9, paste("N = ", round(d[6], 0), sep = ""), cex = 1.2)
plot(masses, zbar_p0_dens[7, 7, ], type = "l", ylim = c(0, 6), las = 1,
    xlab = "", ylab = "", cex.axis = 1.2)
lines(masses, zbar_p1_dens[7, 7, ], col = teal)
text(7.75, 5.9, paste("N = ", round(d[7], 0), sep = ""), cex = 1.2)
plot(masses, zbar_p0_dens[7, 8, ], type = "l", ylim = c(0, 6), las = 1,
    xlab = "", ylab = "", yaxt = "n", cex.axis = 1.2)
lines(masses, zbar_p1_dens[7, 8, ], col = teal)
text(7.75, 5.9, paste("N = ", round(d[8], 0), sep = ""), cex = 1.2)
mtext(side = 1, "body mass (kg)", line = 3)
plot(masses, zbar_p0_dens[7, 9, ], type = "l", ylim = c(0, 6), las = 1,
    xlab = "", ylab = "", yaxt = "n", cex.axis = 1.2)
lines(masses, zbar_p1_dens[7, 9, ], col = teal)
text(7.75, 5.9, paste("N = ", round(d[9], 0), sep = ""), cex = 1.2)
# 12
plot.new()
legend("bottom", lty = 1, col = c(1, teal), c("not pregnant", "pregnant"),
    bty = "n", horiz = TRUE, cex = 1.3)
# 13
plot.new()
dev.off()
```

References

Bérénos, C., Ellis, P.A., Pilkington, J.G. & Pemberton, J.M. 2014. Estimating quantitative genetic parameters in wild populations: a comparison of pedigree and genomic approaches. *Molecular Ecology* **23**: 3434–3451.

Crawley, M.J., Albon, S.D., Bazely, D.R., Milner, J.M., Pilkington, J.G. & Tuke, A.L. 2004. Vegetation and sheep population dynamics. In: *Soay sheep: dynamics and selection in an island population* (T.H. Clutton-Brock & J.M. Pemberton, eds). Cambridge University Press.

Falconer, D. 1981. *Introduction to Quantitative Genetics*, 2nd edn. Longman, New York.

Hadfield, J.D. 2010. MCMC methods for multi-response generalized linear mixed models: the MCMCglmm R package. *Journal of Statistical Software* **33**: 1–22.

Hansen, T.F., Armbruster, W., Carlson, M. & Pelabon, C. 2003. Evolvability and genetic constraints in Dalechampia Blossoms: genetic correlations and conditional evolvability. *Journal of Experimental Zoology* **296B**: 23–39.

Henderson, C.R. 1975. Best linear unbiased estimation and prediction under a selection model. *Biometrics* **31**: 423–447.

Morrissey, M.B., Kruuk, L.E.B. & Wilson, A.J. 2010. The danger of applying the breeder's equation in observational studies of natural populations. *Journal of Evolutionary Biology* **23**: 2277–2288.

Rausher, M.D. 1992. The measurement of selection on quantitative traits: biases due to environmental covariances between traits and fitness. *Evolution* **46**: 616–626. doi:10.1111/j.1558-5646.1992.tb02070.x. URL `https://onlinelibrary.wiley.com/doi/abs/10.1111/j.1558-5646.1992.tb02070.x`.

Robert, C.P. & Casella, G. 2005. *Monte Carlo statistical methods*, 2nd edn. Springer texts in statistics. Springer, Berlin. URL `https://cds.cern.ch/record/1187871`.

Villemereuil, P.D., Schielzeth, H., Nakagawa, S. & Morrissey, M. 2016. General methods for evolutionary quantitative genetic inference from generalised mixed models. *Genetics* **204**: 1281–1294.
